# Compendium of 4,941 rumen metagenome-assembled genomes for rumen microbiome biology and enzyme discovery

**DOI:** 10.1038/s41587-019-0202-3

**Published:** 2019-08-02

**Authors:** Robert D. Stewart, Marc D. Auffret, Amanda Warr, Alan W. Walker, Rainer Roehe, Mick Watson

**Affiliations:** 1 The Roslin Institute and the Royal (Dick) School of Veterinary Studies, University of Edinburgh, Easter Bush, UK; 2 Scotland’s Rural College, Edinburgh, UK; 3 The Rowett Institute, University of Aberdeen, Aberdeen, UK

**Keywords:** Microbial communities, Environmental microbiology, Databases, Genome informatics, Applied microbiology

## Abstract

Ruminants provide essential nutrition for billions of people worldwide. The rumen is a specialized stomach that is adapted to the breakdown of plant-derived complex polysaccharides. The genomes of the rumen microbiota encode thousands of enzymes adapted to digestion of the plant matter that dominates the ruminant diet. We assembled 4,941 rumen microbial metagenome-assembled genomes (MAGs) using approximately 6.5 terabases of short- and long-read sequence data from 283 ruminant cattle. We present a genome-resolved metagenomics workflow that enabled assembly of bacterial and archaeal genomes that were at least 80% complete. Of note, we obtained three single-contig, whole-chromosome assemblies of rumen bacteria, two of which represent previously unknown rumen species, assembled from long-read data. Using our rumen genome collection we predicted and annotated a large set of rumen proteins. Our set of rumen MAGs increases the rate of mapping of rumen metagenomic sequencing reads from 15% to 50–70%. These genomic and protein resources will enable a better understanding of the structure and functions of the rumen microbiota.

## Abstract (web summary)

Comprehensive resource of cattle rumen genomes and a database of predicted proteins.

## Main

Ruminants convert human-inedible, low-value plant biomass into products of high nutritional value, such as meat and dairy products. The rumen, which is the first of four chambers of the stomach, contains a mixture of bacteria, archaea, fungi and protozoa that ferment complex carbohydrates, including lignocellulose and cellulose, to produce short-chain fatty acids (SCFAs) that the ruminant uses for homeostasis and growth. Rumen microbes are a rich source of enzymes for plant biomass degradation for use in biofuel production^1–3^, and manipulation of the rumen microbiome offers opportunities to reduce the cost of food production^4^.

Ruminants are important for both food security and climate change. For example, methane is a byproduct of ruminant fermentation, released by methanogenic archaea, and an estimated 14% of methane produced by humans has been attributed to ruminant livestock^5^. Methane production has been directly linked to the abundance of methanogenic archaea in the rumen^6^, offering possibilities for mitigating this issue through selection^7^ or manipulation of the microbiome. Two studies have reported large collections of rumen microbial genomes. Stewart et al. assembled 913 draft MAGs (named rumen-uncultured genomes (RUGs)) from the rumens of 43 cattle raised in Scotland^8^, and Seshadri et al. reported 410 reference archaeal and bacterial genomes from the Hungate collection^9^. As isolate genomes, the Hungate genomes are generally higher quality and, crucially, the corresponding organisms exist in culture and so can be grown and studied in the lab. However, we found that addition of the Hungate genomes increased read classification by only 10%, as compared to an increase of 50–70% when the RUGs were used, indicating large numbers of undiscovered microbes in the rumen.

We present a comprehensive analysis of more than 6.5 terabases of sequence data from the rumens of 283 cattle. Our catalog of rumen genomes (named RUG2) includes 4,056 genomes that were not present in Stewart et al.^8^, and brings the number of rumen genomes assembled to date to 5,845. We also present a metagenomic assembly of nanopore (MinION) sequencing data (from one rumen sample) that contains at least three whole bacterial chromosomes as single contigs. These genomic and protein resources will underpin future studies on the structure and function of the rumen microbiome.

## Results

### Metagenome-assembled genomes from the cattle rumen

We sequenced DNA extracted from the rumen contents of 283 beef cattle (characteristics of the animals sequenced are in Supplementary Data 1), producing over 6.5 terabytes of Illumina sequence data. We operated a continuous assembly-and-dereplication pipeline, which means that newer genomes of the same strain (>99% average nucleotide identity (ANI)) replaced older genomes if their completeness and contamination statistics were better. All 4,941 RUGs we present here have completeness ≥80% and contamination ≤10% (Supplementary Fig. 1).

**Fig. 1:**
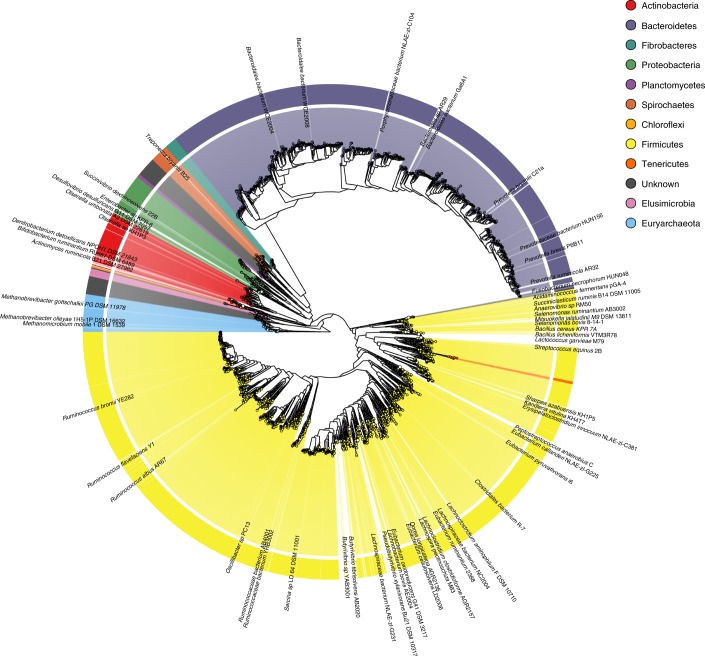
Phylogenetic tree of 4,941 RUGs from the cattle rumen, additionally incorporating rumen genomes from the Hungate collection. The tree was produced from concatenated protein sequences using PhyloPhlAn^13^, and subsequently drawn using GraPhlAn^45^. Labels show Hungate genome names, and were chosen to be informative but not overlap.

All the RUGs were analyzed using MAGpy^10^ and their assembly characteristics, putative names and taxonomic classifications are given in Supplementary Data 2. Sourmash^11^, DIAMOND^12^ and PhyloPhlAn^13^ outputs, which reveal genomic and proteomic similarity to existing public data, are given in Supplementary Data 3. A phylogenetic tree of the 4,941 RUGs, alongside 460 public genomes from the Hungate collection, is presented in Fig. 1 and Supplementary Data 4. The tree is dominated by large numbers of genomes from the Firmicutes and Bacteroidetes phyla (dominated by Clostridiales and Bacteroidales, respectively), but also contains many new genomes from the Actinobacteria, Fibrobacteres and Proteobacteria phyla. Clostridiales (2,079) and Bacteroidales (1,081) are the dominant orders, with Ruminoccocacae (1,111) and Lachnospiraceae (640) constituting the dominant families within Clostridiales and Prevotellaceae (521) consituting the dominant family within Bacteroidales.

The Genome Taxonomy Database (GTDB) proposed a new bacterial taxonomy based on conserved concatenated protein sequences^14^, and we include the GTDB-predicted taxa for all RUGs (Supplementary Data 3). A total of 4,763 RUGs had <99% ANI with existing genomes, and 3,535 had <95% ANI with existing genomes and therefore represent potential new species.

Of the 4,941 genomes, 144, were classified to the species level, 1,092 were classified to the genus level, 3,188 were classified to the family level, 4,084 were classified to the order level, 4,514 were classified to the class level, 4,801 were classified to the phylum level and 4,941 were classified to the kingdom level. Of the genomes classified at the species level, 43 represented genomes derived from uncultured strains of *Ruminococcus flavefaciens*, 42 represented genomes from uncultured strains of *Fibrobacter succinogenes*, 18 represented genomes from uncultured strains of *Sharpea azabuensis* and 10 represented genomes from uncultured strains of *Selenomonas ruminantium*. These species belong to genera known to play an important role in rumen homeostasis^15^.

We assembled 126 archaeal genomes, 111 of which were species of *Methanobrevibacter*. There are two other members of the Methanobacteriaceae family, which were both predicted to be members of the *Methanosphaera* genus by GTDB. Nine of the archaeal RUGs had sourmash hits to *Candidatus* Methanomethylophilus sp. 1R26; a further three had weak sourmash hits to Methanogenic archaeon ISO4-H5; and the remaining archaeal genome had no sourmash hits, and weaker DIAMOND hits to the same genome (Methanogenic archaeon ISO4-H5). All 13 were predicted to be members of the genus *Candidatus* Methanomethylophilus by GTDB, but this is based on similarity to only two genomes, both of which have uncertain phylogenetic lineages. If *Candidatus* Methanomethylophilus is a true genus, then our dataset increases the number of sequenced genomes from 2 to 15.

Genome quality statistics were measured by analyzing single-copy core genes (Supplementary Fig. 1). There are different standards for the definition of MAG quality. Bowers et al.^16^ describe high-quality drafts as having ≥90% completeness and ≤5% contamination; 2,417 of the RUGs met these criteria. Alternatively, Parks et al.^17^ define a quality score as completeness − (5 × contamination) and exclude any MAG with a score less than 50; 4,761 of the RUGs met this criterion, although, whilst the MAGs from Parks et al. could have completeness as low as 50%, the genomes presented here were all ≥80% complete. The RUGs ranged in size from 456 kilobases (kb) to 6.6 megabases (Mb), with N50 values (50% of assembled bases in contigs larger than the N50 value) ranging from 4.5 kb to 1.37 Mb. The average number of tRNA genes per RUG was 16.9, and 446 of the RUGs had all 20. As assemblies of Illumina metagenomes struggle to assemble repetitive regions, most of the RUGs did not contain a 16S rRNA gene—464 RUGs encoded a fragment of the 16S rRNA gene, and 154 encoded at least one full-length 16S rRNA gene.

The coverage of each RUG in each sample is provided in Supplementary Data 5. Using a cut-off of 1× coverage, most RUGs (4,863) were present in more than one animal, 3,937 RUGs were present in more than ten animals and 225 RUGs were present in more than 200 animals. One RUG was present in all animals, RUG11026, which was a member of the Prevotellaceae family.

### A near-complete single-contig Proteobacteria genome

Metagenomic assembly of Illumina data often results in highly fragmented assemblies, but RUG14498, an uncultured Proteobacteria species (genome completeness 87.91% and contamination 0%), had 136 of 147 single-copy genes present with no duplications in a single contig of just over 1 Mb in size. Proteobacteria with small genomes (<1.5 Mb in size) were relatively common (*n* = 67) in our dataset and have also been found in other large metagenome assembly projects^17^. The Proteobacteria genomes we present encode proteins with only 45–60% amino acid identity with proteins in UniProt TREMBL^18^. We compared our single-contig Proteobacteria assembly with nine Proteobacteria with similarly sized genomes assembled by Parks et al.^17^ (Supplementary Fig. 2). ANI, which is often used to delineate new strains and species, between the nine UBA genomes and RUG14498 was revealing. UBA2136, UBA1908, UBA3307, UBA3773 and UBA3768 had no detectable level of identity with any other genome in the set; UBA4623, UBA6376, UBA6864 and UBA6830 all had greater than 99.4% ANI with one another, indicating that they are highly similar strains of the same species. UBA4623, UBA6376, UBA6864 and UBA6830 also had around 77.8% ANI with RUG14498, suggesting that the single-contig RUG14498 is a high-quality, near-complete whole genome of a new Proteobacteria species. The single-contig RUG14498 was assembled by IDBA_ud from sample 10678_020. IDBA_ud exploits uneven depth in metagenomic samples to improve assemblies. RUG14498 was the tenth most abundant genome in 10678_020, and other genomes of similar depth in that sample were taxonomically unrelated, enabling IDBA_ud to assemble almost the entire genome in a single contig.

RUG14498 had a single full-length 16S rRNA gene (1,507 base pairs). The top hit in GenBank (97% identity across 99% of the length) was accession AB824499.1, a sequence from an uncultured bacterium from the rumen of Thai native cattle and swamp buffaloes. The top hit in SILVA^19^ was to the same sequence, only this time annotated as an uncultured *Rhodospirillales*. Together, these results support the conclusion that RUG14498 represents a new Proteobacteria species. Low amino acid identity to known proteins limited our ability to predict function and metabolic activity; nevertheless, RUG14498 encodes 73 predicted CAZymes, including 42 glycosyl transferases and 19 glycosyl hydrolases, suggesting a role in carbohydrate synthesis and metabolism.

### New microbial genomes from the rumen microbiome

**Fig. 2:**
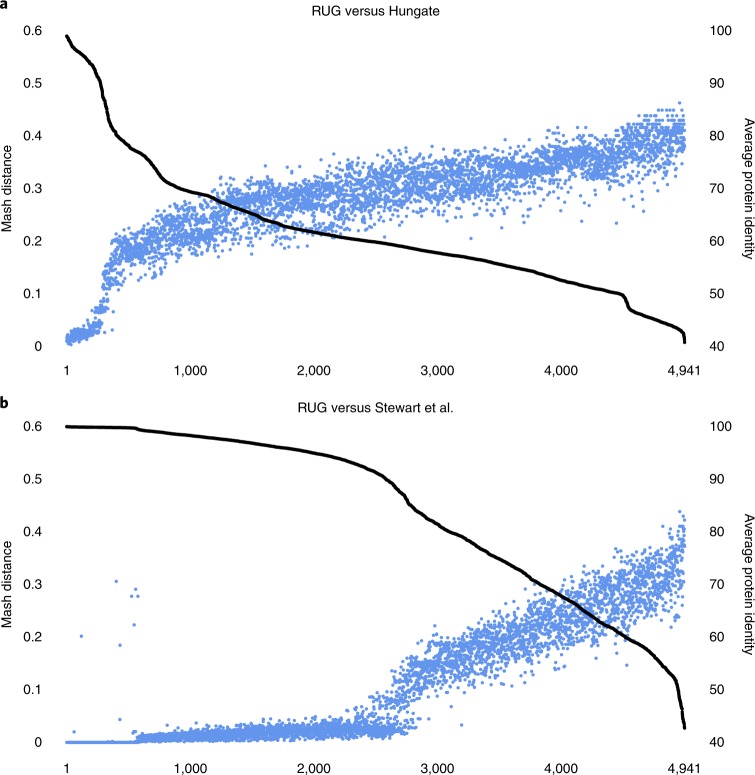
A comparison of the RUG dataset with the Hungate collection and previously published data. **a**,**b**, A comparison of the 4,941 RUGs with the Hungate collection (**a**) and our previously published data from Stewart et al.^8^ (**b**). The black line indicates the average percentage protein identity with the closest match (right-hand *y* axis), and blue dots indicate the mash distance (*k* = 100,000) between each RUG and the closest match in the comparison dataset (a measure of dissimilarity between two DNA sequences). As expected, a high protein identity relates to a low mash distance, and vice versa. The RUGs are sorted independently by average protein identity for **a** and **b**. There is a clear inflection point in Fig. 2b, roughly half way along the *x* axis, where the protein identity dips below 90% and the mash distance rises, neatly demonstrating the novelty represented by our new larger dataset.

We compared the 4,941 RUGs to the Hungate collection and to our previous dataset^8^ (Fig. 2). Of the 4,941 RUGs, 149 had >95% protein identity with Hungate members and 271 had >90%; this left 4,670 RUGs with <90% protein identity with Hungate members. Of the 4,941 RUGs, 2,387 had <90% protein identity with genomes in Stewart et al., and more than 1,100 RUGs had <70% protein identity with genomes in Stewart et al. Many of the RUGs with the lowest protein identity to publicly available genomes could not be classified beyond the phylum level, and some are classified as simply uncultured bacterium.

We compiled a database comprising all RUG genomes, the Hungate collection genomes^9^ and rumen MAGs from Hess et al.^1^, Parks et al.^17^, Solden et al.^20^ and Svartström et al.^21^ that we name the rumen superset. The rumen superset was dereplicated at both 99% (strain level) and 95% (species level) ANI. At 95% ANI, the rumen superset was reduced to 2,690 clusters, representing species-level bins. Of these clusters, 2,078 contained only RUG genomes, and therefore represent putative new rumen microbial species identified in this study. Fifty-eight clusters contained both Hungate and RUG genomes, and 268 clusters contained only Hungate genomes (Supplementary Data 6). At 99% ANI, the rumen superset was reduced to 5,574 clusters, representing strain-level bins. Of these clusters, 4,845 contained only RUG genomes, and may represent putative new rumen microbial strains (Supplementary Data 7). Supplementary Figure 3 shows how the various rumen MAG sets overlap at 95% ANI after dereplication.

We calculated an estimate of the completeness of the RUG2 dataset using the Chao 1 estimator^22^ (we were only able to do this for our own dataset, as the estimate was based on the number of times species were observed at different frequencies, and we did not have these values for other datasets). Dereplicating all RUG genomes at 95% ANI gave us 2,180 species-level bins. Of these, 948 were singletons (that is, were observed exactly once), and 410 were doublets (that is, were observed exactly twice). Using the Chao 1 formula, we predicted 3,276 species, we therefore estimate that we have discovered 66.54% of the species present in our samples.

We assessed the impact of using rumen genomic data on the read classification rates of several public datasets using three databases—the first, our custom rumen kraken database, consisted of RefSeq complete genomes and the Hungate collection^23,24^; the second was the same database plus only the RUGs; and the third was the same database plus the rumen superset (which includes the RUGs). We classified the following five datasets—our own (Stewart et al.^8^), a dataset we previously published (Wallace et al.^6^), data from 14 cattle from a study on niche specialization (Rubino et al.^25^), data from a methane emissions study of sheep (Shi et al.^26^) and data from a recent metagenomic study of moose (Svartström et al.^21^) (Supplementary Fig. 4).

The classification rate was increased by using either the RUG or rumen superset database, although using the rumen superset resulted in only a marginal increase in most cases. We improved read classification rates from 15% to 70%, with more than a quarter of our samples achieving a classification rate of 80% or higher. These rates were comparable with read classification rates for the human microbiome as reported by Pasolli et al.^27^.

### Strain-level analysis of methane emissions in sheep

Previously Shi et al.^26^ found no significant changes in microbiota community structure between low-methane-emitting (LME) and high-methane-emitting (HME) sheep, although there were differences in gene expression between the two groups. We reanalyzed the dataset from Shi et al*.* using our rumen metagenomic data; specifically, we used our custom kraken database consisting of RefSeq genomes and the rumen superset to classify reads at the level of kingdom, phylum, family, genus and species, and tested differences between LME and HME sheep. While we found no significant differences at the level of kingdom, we found significant and profound differences at every other taxonomic level tested (Supplementary Tables 1–5 and Supplementary Figs. 5–9). At the genus level, *Sharpea*, *Kandleria*, *Fibrobacter* and *Selenomonas* were associated with LME sheep and *Elusimicrobium* was associated with HME sheep (Supplementary Table 4). At the species level, we found that 340 species differed significantly between LME and HME sheep (Supplementary Table 5), including 11 species of *Bifidobacterium* and 6 species of *Olsenella* that were significantly more proportionally abundant in LME sheep and 9 species of *Desulfovibrio* that were significantly more proportionally abundant in HME sheep. *Fibrobacter succinogenes*, an important rumen microbe known to be heavily involved in the degradation of plant fibers, was also significantly different between the two groups, and was associated with LME sheep. Some of these microbes were previously identified as differentially proportionally abundant between LME and HME sheep^15,28^ using marker-gene sequencing, but our results provide greater resolution and reveal the genome sequences involved.

Kraken classifies data at different levels of the NCBI taxonomy; unfortunately, this does not give data on the RUGs that do not yet have specific NCBI taxonomy IDs. Therefore, to estimate the abundance of individual strains, we aligned reads directly to the rumen superset, and used the number of reads designated as primary alignments as a proxy for the relative abundance of each genome. At a false discovery rate ≤ 0.05, 1,709 genomes showed differentially proportional abundance between LME and HME sheep (Supplementary Data 8 and Supplementary Fig. 10). In Supplementary Fig. 10, LME and HME sheep are clearly separated along principal component 1, which explained 58% of the variance in the data. Supplementary Data 8 lists the differentially abundant genomes. Of note were the large numbers of previously uncharacterized Lachnospiraceae species associated with LME sheep and 22 strains of *S. azabuensis* that all had higher proportional abundance in LME sheep (all 18 *S. azabuensis* RUGs and 4 *S. azabuensis* strains from the Hungate collection). These results agree with previous studies based on marker-genes^15^, and our dataset increases the number of publicly available genomes for *S. azabuensis* from 4 to 22. Large numbers of uncharacterized Ruminococcaceae and Bacteroidia were also associated with HME sheep. Multiple strains of uncharacterized Proteobacteria, including RUG14498 described above, were more proportionally abundant in HME sheep, and *Fibrobacter* strains were almost all associated with LME sheep.

The relationship between proportional abundance of archaea and methane emissions is not simple. Most archaeal strains were present at similar abundance in LME and HME sheep (Supplementary Data 8). RUGs representing new strains of *Methanobrevibacter* were often more abundant in HME sheep. The RUG with the most striking proportional abundance was RUG12825, which is likely a member of the *Methanosphaera* genus, and was more abundant in LME sheep. The complex relationship between relative abundance of methanogens and methane emissions may underlie our inability to find significant differences in overall archaeal proportional abundance.

That notwithstanding, these data represent a strain-level view of methane emissions in sheep, and support the hypothesis that there are major fundamental changes in rumen metagenomic relative abundance associated with the extremes of low and high methane emissions.

### Global rumen census updated

The global rumen census attempted to determine the core rumen microbiome by using 16S rRNA sequencing of rumen samples from 742 individual animals from around the world, comprising eight ruminant species^29^. *Prevotella*, *Butyrivibrio* and *Ruminococcus*, as well as unclassified Lachnospiraceae, Ruminococcaceae, Bacteroidales and Clostridiales, were the dominant rumen bacteria and may represent a core bacterial rumen microbiome. The same species were abundant in our data (Supplementary Data 5). We also found that many Proteobacteria were highly abundant, including *Succinivibrio* (Supplementary Data 5). This is noteworthy because Proteobacteria were found to be highly abundant in many of the samples from the rumen census, but were not highlighted as being part of the core rumen microbiome.

To further characterize the proportional abundance of Proteobacteria, we used the rumen superset database to classify data from this study, Wallace et al.^6^, Rubino et al.^25^, Shi et al.^15^ and Svartström et al.^21^ (Supplementary Fig. 11). Proteobacteria were present in all datasets; they were abundant in cattle datasets, but less so in moose and sheep. Given the high proportional abundance of Proteobacteria in many samples, and their consistent presence in all of the samples we tested, we suggest adding Proteobacteria to the core bacterial rumen microbiome that was proposed by Henderson et al.^29^.

### Long-read assembly of complete bacterial chromosomes

**Fig. 3:**
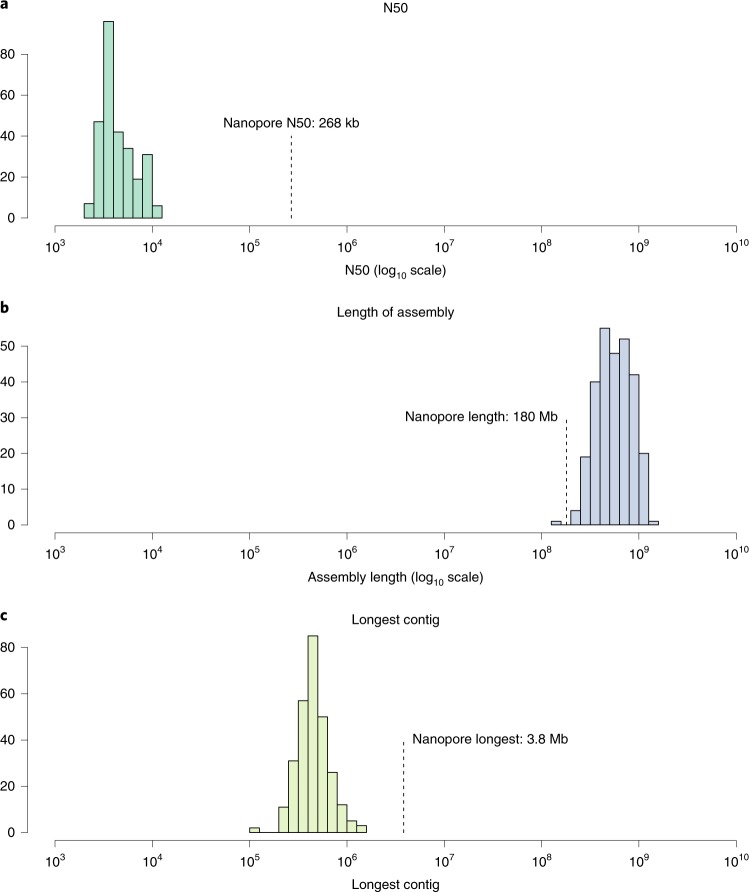
A comparison of Illumina and nanopore metagenomic assembly statistics. The colored histograms show the distribution of statistics for 282 Illumina assemblies, and the single nanopore assembly is highlighted. **a**, N50 values. **b**, Total length of the assembly. **c**, Length of the longest contig. The nanopore assembly N50 of 268 kb was over 56 times longer than that for the average Illumina assembly (4.7 kb), the Illumina assemblies were often longer (average of 600 Mb), the nanopore assembly (at 178 Mb in length) was not the shortest of the assemblies we produced and the nanopore assembly produced the longest contig at 3.8 Mb, seven times longer than the average for the Illumina assemblies (479 kb) and 2.74 times longer than the longest single Illumina contig (1.38 Mb; one of 13 contigs from the 99.19% complete uncultured *Bacteroidia* bacterium RUG14538). In terms of a direct comparison, the Illumina-only assembly of the same sample had an N50 of 12.2 kb, a total length of 247 Mb and a longest contig of 358 kb.

We analyzed a single sample (10572_0012) using a MinION sequencer and a comparison of Illumina and MinION assembly statistics is presented in Fig. 3. Three flow cells produced 11.4 gigabases of data with an N50 value of 11,585 base pairs. The mean read length was 6,144 base pairs, which is short in comparison to other reports^30,31^. We attribute this to short DNA fragments and nicks caused by the bead beating step during DNA extraction. We assembled long reads using Canu^32^, to form an assembly of 178 Mb in length with an N50 of 268 kb. Regardless of length, Canu predicted 31 of the contigs to be circular. These circular contigs might represent putative plasmids or other circular chromosomes.

One problem with single-molecule sequencing technologies is the presence of post-assembly insertions and deletions (indels)^33^. Canu can correct reads but not enough to remove all indels. Detecting sequencing errors without a ground truth dataset is difficult, so we hypothesized that most indels would create premature stop codons and that gene prediction tools (for example, Prodigal^34^) would produce truncated proteins. We examined the ratio between the lengths of predicted proteins and their top hits in UniProt to estimate indels (Supplementary Fig. 12). Although these data indicate multiple errors as compared to the Illumina short-read data, we corrected errors by polishing with one round of Nanopolish and two rounds of Racon. We set up a software pipeline to calculate statistics and produce similar plots for any input genome or metagenome called IDEEL.

Statistics for all contigs ≥500 kb and all contigs predicted to be circular are provided in Supplementary Data 9. The nanopore assembly contained several single contigs that we predict are complete, or near-complete, circular whole chromosomes.

*Prevotella copri* nRUG14950 (tig00000032) was a single contig of 3.8 Mb, which most closely resembled *Prevotella copri* DSM 18205, and which showed high similarity to RUG14032. *Prevotella copri* nRUG14950 was predicted to be 98.48% complete by CheckM^35^, with a contamination score of 2.03%, whereas RUG14032 was estimated to be 96.62% complete with a contamination score of 1.35%. Comparative alignments between *Prevotella copri* nRUG14950, RUG14032 and *Prevotella copri* DSM 18205 are shown in Supplementary Fig. 13. There was a clear and striking relationship between *Prevotella copri* nRUG14950 and RUG14032. These two genomes, both estimated to be nearly complete, were assembled from different samples using different techniques, and sequenced with different sequencing technologies. Our assembly of *Prevotella copri* nRUG14950 consisted of only one contig and was estimated to be 98.48% complete, representing the most continuous chromosomal assembly of *Prevotella copri*, despite having been assembled from a metagenome.

*Selenomonas* spp. nRUG14951 was a single contig of 3.1 Mb in length that was predicted to be circular, with completeness and contamination statistics of 98.13% and 0.16%, respectively. The most similar RUG was RUG10160, with a mean of 94% protein identity. RUG10160 was estimated to be 97.66% complete and 0% contaminated. However, the closest public reference genome was *Selenomonas ruminantium* GACV-9, part of the Hungate collection, which had only ~64% protein identity with *Selenomonas* spp. nRUG14951. There was a good whole-genome alignment between *Selenomonas* spp. nRUG14951 and RUG10160 (Supplementary Fig. 14), albeit with some evidence of rearrangements and some small sections of the genome that were only captured by the nanopore assembly.

We also identified Lachnospiraceae bacterium nRUG14952, which had a 2.5-Mb circular, near-complete genome (95.46%), a second RUG13141 (which had 96% protein identity to nRUG14952) and a more distantly related public reference genome (Lachnospiraceae bacterium KHCPX20, which has 63% protein identity to nRUG14952). The nanopore-assembled genome Lachnospiraceae bacterium nRUG14952 contained several genome regions that were absent from RUG13141 (Supplementary Fig. 15).

nRUG14951 and nRUG14952 represent entire bacterial chromosomes assembled as single contigs and are the first genome assemblies for these species. The remainder of the nanopore assembly contained highly continuous contigs that represent large portions of previously unsequenced bacterial chromosomes. These results taken together demonstrate the power of long reads for assembling complete chromosomes from complex metagenomes.

To assess the advantage of having complete chromosomal assemblies, we annotated the three nanopore whole genomes and the three genomes of their closely related RUGs (Supplementary Data 10). The three complete nanopore genomes contained five, seven and three full-length 16S gene sequences, whereas all three RUGs contained none. In addition, the three nanopore genomes were massively enriched for IS family transposase proteins as compared to their RUG counterparts. Transposases are associated with insertion sequences in bacterial genomes, and catalyze the transposition of mobile elements^36^. Finally, in all cases, the nanopore assemblies had more annotated clusters of orthologous genes, suggesting that they have more complete functional annotation than their short-read counterparts.

### A protein database for rumen microbial proteomics

We put together a non-redundant dataset of rumen proteins from the 4,941 RUGs and 460 publicly available genomes from the Hungate collection (10.69 million proteins), following the model of UniRef^37^ and clustering the protein set at 100% (9.45 million clusters), 90% (5.69 million clusters) and 50% (2.45 million clusters) identity to form RumiRef100, RumiRef90 and RumiRef50, respectively.

To assess the protein-level difference between our dataset and other rumen MAG datasets, we took RumiRef100 and added over 900,000 predicted proteins from the rumen superset. We clustered these at 90% identity, which resulted in 6.24 million protein clusters. Of these, 5 million clusters contained at least one RUG protein, 4.74 million contained only RUG proteins and 3.67 million were singletons that contained only RUG proteins.

All 10.69 million predicted proteins from the RUGs were compared to KEGG^38^, 460 public genomes from the Hungate collection, UniRef100, UniRef90 and UniRef50. The mean protein identities of the top hit for these databases were 55.88%, 63.58%, 67.52%, 67.25% and 59.97%, respectively. These data provide a comprehensive and richly annotated protein dataset from the rumen.

The RUG proteins were compared to the CAZy^39^ database (31 July 2018) using dbCAN2 (ref. ^40^). A total of 442,917 were predicted to be involved in carbohydrate metabolism, including 235,001 glycoside hydrolases, 120,494 glycosyl transferases, 55,523 carbohydrate esterases, 23,928 proteins with carbohydrate-binding modules, 6,834 polysaccharide lyases, 907 proteins with predicted auxiliary activities, 80 proteins with a predicted cohesin domain and 150 proteins with an S-layer homology module (SLH).

**Fig. 4:**
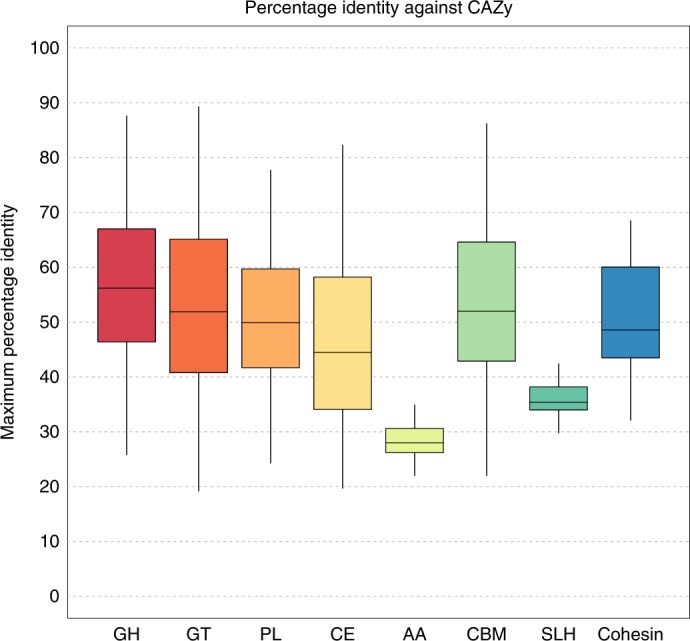
Maximum percentage identity between CAZyme-predicted proteins from the RUGs and the CAZy database. GH, glycoside hydrolase (*n* = 235,001); GT, glycosyl transferase (*n* = 120,494); PL, polysaccharide lyase (*n* = 6,834); CE, carbohydrate esterase (*n* = 55,523); AA, auxiliary activities; CBM, carbohydrate-binding module (*n* = 23,928); SLH, S-layer homology domain (*n* = 150); cohesin, cohesin domain (*n* = 80). Center lines indicate the median value; boxes show the interquartile range; and whiskers extend to the most extreme data point that is no more than 1.5 times the interquartile range from the box.

The similarity of the predicted CAZymes to the current CAZy database can be seen in Fig. 4. None of the eight classes of carbohydrate-active enzymes displayed an average protein identity greater than 60% indicating that CAZy poorly represents the diversity of CAZymes encoded in the genomes of ruminant microbes. Of particular note is the class AA ‘auxiliary activities’, with an average protein identity of less than 30% between CAZy and the RUG CAZymes. AA was created by CAZy to classify ligninolytic enzymes and lytic polysaccharide monooxygenases (LPMOs).

**Fig. 5:**
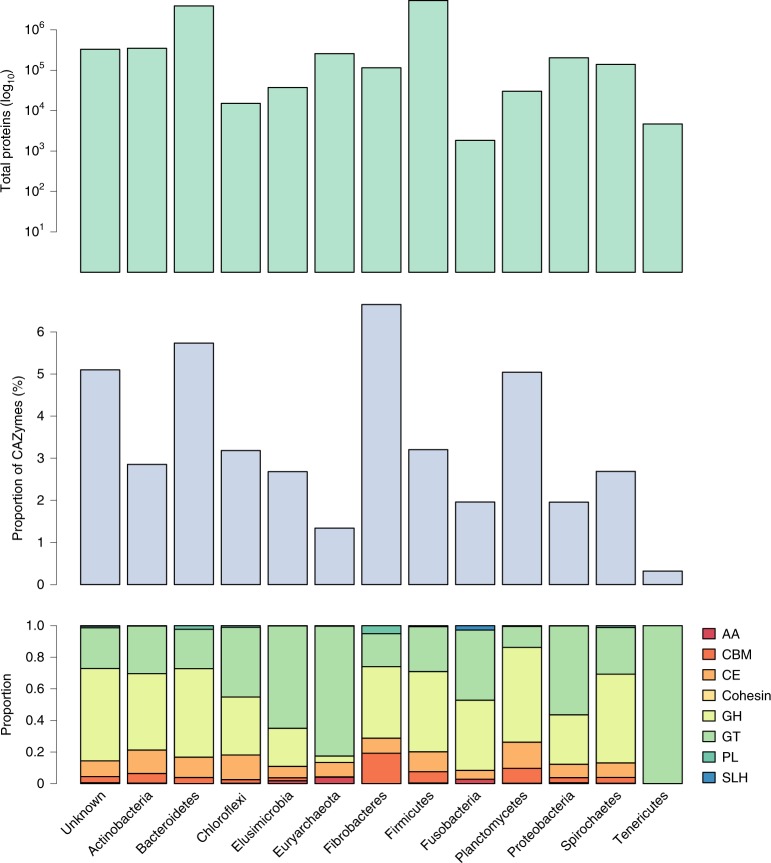
Taxonomic and functional distribution of proteins. Top, total number of proteins for 12 phyla and the group of unknown bacteria. Middle, percentage of the proteome predicted to be CAZymes. Bottom, distribution of eight CAZyme classes as a proportion of the total number of predicted CAZymes.

The distribution of CAZymes across 12 different phyla and the group of unknown bacteria can be seen in Fig. 5. The Bacteroidetes (3.9 million) and Firmicutes (5.3 million) together contributed the largest number of proteins to our dataset; however, whereas 5.7% of the proteome of Bacteroidetes was devoted to CAZyme activity, in Firmicutes the figure was 3.2%. Fibrobacteres devoted the highest percentage of their proteome to carbohydrate metabolism (over 6.6%), as was expected owing to their fiber-attached, high-cellulolytic activity. Only a few studies exist on the role of Planctomycetes in the rumen^24,41,42^; however, while they contributed a relatively low number of proteins in our dataset (30,172), just over 5% of those proteins were predicted to be CAZymes, suggesting a role in, and adaptation to, carbohydrate metabolism. Of the 80 cohesin-containing proteins, 79 were encoded by the Firmicutes (the remaining one was encoded by an unknown bacterium), as were 101 of 149 SLH-domain-containing proteins. Both are components of cellulosomes, multienzyme complexes that are involved in fiber degradation, which are encoded by some members of the Clostridiales family.

There were 1,707 Bacteroidetes genomes in the RUGs, and additionally we had a whole genome of *Prevotella copri* from the nanopore assembly. These 1,708 genomes were subjected to prediction of polysaccharide utilization loci (PULz) using our pipeline PULpy^43^. Of the 1,708 genomes, 1,469 were predicted to have at least one PUL, and in total there were 15,629 separate loci involving 88,260 proteins. The highest numbers of PULs per genome were 52 for RUG13980 and 50 for RUG10279; both these were labeled as uncultured Prevotellaceae and both of these genomes are closely related to *Prevotella multisaccharivorax*, which is known to be able to utilize multiple carbohydrate substrates^44^.

## Discussion

The rumen microbiome has a crucial role in food security and climate change. Recent studies have released more than 1,300 draft and complete rumen genomes. We add 4,941 near-complete, dereplicated metagenome-assembled genomes to these 1,300 existing rumen genomes^9,20,21^. By combining our dataset with publicly available genomes, we assembled a rumen superset of 5,845 publicly available bacterial and archaeal genomes. This set contains 2,690 unique species-level bins (95% ANI), and 2,078 of these 2,690 putative species are RUG2 genomes discovered in this study. The RUG2 dataset and the rumen superset bring read classification rates up to 70% for our own data and 45–55% for other rumen metagenome datasets (some from non-cattle ruminants). The remaining reads are likely to derive from low-abundance bacterial and archaeal species, difficult-to-assemble genomes, and the fungal, protozoan and viral genomes that are not part of this study.

We estimate that we have discovered 65% of rumen species in our samples, representing four important beef cattle breeds, which suggests that there are over 1,000 species yet to be sequenced and assembled. Given that average read classification rates dip from 70% in our own data to 50% in the cattle data of Rubino et al. (Limousin × Friesian cross)^25^ and the sheep data of Shi et al. sheep data^26^ and to 45% in the moose data^21^, there are many species yet to be discovered, and there are likely to be species- and breed-specific rumen microbiomes. We note the high abundance of unclassified Proteobacteria in our data, as well as in the rumen census data, and suggest that these may form part of a core rumen microbiome. Our dataset contains 74 proteobacterial genomes, and we present one near-complete genome in a single contig.

We apply our dataset to reanalyze data on methane emissions in sheep that were published in 2014^26^. Using a combined database of rumen microbial genomes, we reveal fundamental and large-scale differences in rumen metagenomic abundance between LME and HME sheep. These differences occur at almost every taxonomic level tested, and the rumen superset database enabled us to analyze these data at high resolution. While species- and strain-level metagenomic data must always be interpreted with care, there remains a possibility that strains that are not present in the database are driving the observed differences. Nonetheless, we observed consistent patterns suggesting large changes in abundance for numerous species. Our analysis supports subsequent studies of methane emissions in sheep^15,28^ by identifying specific strains of bacteria and archaea involved, and revealing their genome sequence. Our analysis confirmed that there was a complex relationship between archaeal abundance and methane emissions, with archaeal species and strains both positively and negatively associated with methane emissions. These insights into metagenomic species- and strain-level aspects of methane emissions will form the basis of future studies.

The main rumen functions rely on the activity of proteins encoded in rumen microbe genomes, and as researchers produce more proteomic data, it is vital that protein reference datasets be available. We present a dataset of large redundant and non-redundant rumen microbial protein predictions, and provide rich annotation using public protein, pathway and enzyme databases. This resource will enable researchers to predict the function of each protein, and better assess the functional consequences of changes in the rumen proteome.

Going forward, it is vital that more rumen bacteria and archaea be brought into culture, to better enable studying the functions of the rumen microbiome. In particular, if we are to design rational interventions to manipulate rumen feed conversion or methane emissions, we will need to understand microbiome structure, the substrates that are utilized by microbiota and how the microbiota interacts with one another and the ruminant host. Sequencing and assembling rumen microbial genomes is an important step toward improved culture collections and future manipulation of the rumen microbiome for human benefit.

## Methods

### Metagenomic samples

Animal experiments were conducted at the Beef and Sheep Research Centre of Scotland’s Rural College (SRUC). The experiment was approved by the Animal Experiment Committee of SRUC and was conducted in accordance with the requirements of the UK Animals (Scientific Procedures) Act 1986.

The data were obtained from three cross breeds (Aberdeen Angus, Limousin and Charolais) and one pure breed (Luing) (Supplementary Data 4). As previously described, the animals were slaughtered in a commercial abattoir where two post-mortem digesta samples (approximately 50 ml) were taken immediately after the rumen was opened to be drained^46,47^. DNA extraction was carried out following the protocol from Yu and Morrison^48^ and was based on repeated bead beating with column filtration. Illumina TruSeq libraries were prepared from genomic DNA and sequenced on an Illumina HiSeq 4000 by EdinburghGenomics.

We experienced severe problems when using MinION for rumen microbiome DNA while following standard recommended protocols, and we hope our adapted methods will be of assistance to others. We found that the DNA did not meet the recommended purity for nanopore library preparation following extraction, according to Nanodrop optical density ratios. RNase treatment using Riboshredder and clean-up with methods such as AMPure XP beads were sufficient to obtain optical density ratios within the recommended range, but DNA from these methods typically led to poor or failed sequencing runs. Successful clean-up reaching recommended optical density ratios that led to successful sequencing runs was carried out using RNase treatment with Riboshredder and a phenol–chloroform purification. One-dimensional libraries were prepared starting with 2 µg of DNA per library following Oxford Nanopore’s SQK-LSK108 one-dimensional ligation protocol with modifications. The incubation in the end preparation stage of the protocol was extended to 30 min at 20 °C and 30 min at 65 °C, and the incubation in the ligation stage was extended to 15 min at room temperature. The optional repair step for formalin-fixed, paraffin-embedded tissues was also carried out. Three sequencing runs were carried out using FLOMIN-106 flow cells on a MinION MK1b housed in the Watson laboratory at the University of Edinburgh.

### Bioinformatics

#### Metagenomic assembly and binning

In total, 282 samples were sequenced for this study generating between 24 and 140 million 150-base-pair paired-end reads per sample. The samples were sequenced in five batches of 48 samples and one batch of 42 samples (this 42-sample batch was the sole basis of Stewart et al.). An additional sample was used for Hi-C sequencing in Stewart et al.^8^, and the metagenome-assembled genomes from that sample are included in the dereplicated set.

Unless otherwise stated, all parameters used were the default. Each sample was assembled and binned individually using coverage and content as previously described^8^. In brief, each sample was assembled using idba_ud^49^ (v.1.1.3) with the options ‘--num_threads 16 --pre_correction --min_contig 300’. BWA MEM^50^ (v.0.7.15) was used to map reads back to the filtered assembly and Samtools^51^ (v.1.3.1) was used to convert to BAM format. Script jgi_summarize_bam_contig_depths from the MetaBAT2^52^ (v.2.11.1) package was used to calculate coverage from the resulting BAM files. A co-assembly was also produced for each of the six batches of samples using MEGAHIT^53^ (v.1.1.1) with options ‘--kmin-1pass -m 60e+10 --k-list 27,37,47,57,67,77,87 --min-contig-len 1000 -t 16’.

Metagenomic binning was applied to both single-sample assemblies and the co-assemblies using MetaBAT252 with options ‘--minContigLength 2000 --minContigDepth 2’. Single-sample binning produced a total of 37,153 bins, and co-assembly binning produced a further 23,335. All 60,743 bins were aggregated and then dereplicated using dRep^54^ (v.1.1.2). The dRep dereplication workflow was used with options ‘dereplicate_wf -p 16 -comp 80 -con 10 -str 100 -strW 0’. Thus, in prefiltering, only bins assessed by CheckM (v.1.0.5) as having both completeness ≥80% and contamination ≤10% were retained for pairwise dereplication comparison (*n* = 10,586). Bin scores were given as completeness − 5 × contamination + 0.5 × log(N50), and only the highest scoring RUG from each secondary cluster was retained in the dereplicated set. For our dataset, 4,941 dereplicated RUGs were obtained.

Note that we operated a continuous dereplication workflow. Therefore all 913 of the RUGs (both MetaBAT2 and Hi-C) we previously published have been merged with the newer RUGs and dereplicated. As a result, while some of the previously published RUGs exist in the newer dataset published here, many have been replaced by newer RUGs of higher quality.

Supplementary Data 5 gives the average depth for each genome in each sample as calculated by script jgi_summarize_bam_contig_depths from the MetaBAT2 (ref. ^52^) (v.2.11.1) package.

#### Metagenomic assignment

The output of metagenomic binning is simply a set of DNA FASTA files containing putative genomes. These were all assessed for completeness and contamination using CheckM^35^ (v.1.0.5). The 4,941 best bins were analyzed using MAGpy^10^, a Snakemake^55^ pipeline that runs a series of analyses on the bins, including CheckM (v.1.0.5); prodigal^34^ (v2.6.3) protein prediction; Pfam_Scan^56^ (v.1.6); a DIAMOND^12^ (v.0.9.22.123) search against UniProt TrEMBL; PhyloPhlAn^13^ (v.0.99); and a sourmash (v.2.0.0) search against all public bacterial genomes. The MAGpy results were used to produce a putative taxonomic assignment for each bin as follows:

- If the proportion of proteins assigned to a species is ≥0.9 and the average amino acid identity is ≥0.95, assign to species on the basis of DIAMOND results; else
- If sourmash score is ≥0.8, assign to species on the basis of sourmash results; else
- If PhyloPhlAn probability is high and the level is genus or species, then assign taxonomy on the basis of PhyloPhlAn results; else
- If the proportion of proteins assigned to a genus is ≥0.9 and the average amino acid identity is ≥0.9, assign to genus on the basis of DIAMOND results; else
- If PhyloPhlAn probability is high or medium and the level is genus, then assign to genus on the basis of PhyloPhlAn results; else
- If PhyloPhlAn probability is high or medium and the level is family, then assign to family on the basis of PhyloPhlAn results; else
- If the proportion of proteins assigned to a family is ≥0.8 and the average amino acid identity is ≥0.6, assign to family on the basis of DIAMOND results; else
- If PhyloPhlAn probability is high or medium and the level is order, then assign to order on the basis of PhyloPhlAn results; else
- If the proportion of proteins assigned to a order is ≥0.6 and the average amino acid identity is ≥0.6, assign to order on the basis of DIAMOND results; else
- If PhyloPhlAn probability is high or medium and the level is class, then assign to class on the basis of PhyloPhlAn results; else
- If PhyloPhlAn probability is high or medium and the level is phylum, then assign to phylum on the basis of PhyloPhlAn results; else
- Assign taxonomy on the basis of CheckM lineage

Importantly, at this stage, these are only putative taxonomic assignments. Using these labels, a phylogenetic tree consisting of the RUGs and genomes from the Hungate collection, produced from concatenated protein subsequences using PhyloPhlAn^13^ (v.0.99), was visually inspected using FigTree (v.1.4.3), iTol^57^ (v.4.3.1) and GraPhlAn^45^ (v.0.9.7). Annotations were improved where they could be—for example, where MAGpy had only assigned a taxonomy at the genus level but the genome clustered closely with a Hungate 1,000 genome annotated at the species level, the annotation was updated. The tree was also rerooted manually at the Bacteria–Archaea branch using FigTree.

#### Genome quality and comparative genomics

Genome completeness and contamination was assessed using CheckM (v.1.0.5) (see above). tRNA genes were annotated using tRNAscan-SE (v.2.0.0) and 16S rRNA genes were predicted using barrnap (v.0.9). Whole-genome alignments were calculated with MUMmer^58^ (v.3.23) using promer to find matches between genomes. ANI was calculated using FastANI (v.1.1). The RUGs were compared to the Hungate collection and our previous dataset using DIAMOND blastp (v.0.9.22.123) and MASH^59^ (v.2.0) with parameters ‘-k 21 -s100000’.

The rumen superset was dereplicated using dRep as above, with ‘-sa 0.99’ for dereplication at 99% ANI and ‘-sa 0.95’ for dereplication at 95% ANI. Overlaps between sets were plotted with UpSetR^60^ (v.1.3.3). Read classification rates were calculated using kraken^61^ (v.0.10.5) with parameters ‘--fastq-input --gzip-compressed --preload --paired’.

#### Analysis of sheep methane data

Reads from the low and high methane samples from Shi et al. were assigned to different taxonomic levels of the rumen superset database using kraken, as described above. The resulting read counts data were used as input into DESeq2 (v.1.22.2) for differential analysis. Principal-component analysis plots were created using the plotPCA() function within DESeq2, and heat maps were created using the heatmap.2() function within the gplots package (v.3.0.1.1). For strain-level analysis, reads from the low- and high-methane samples from Shi et al. were aligned directly to the rumen superset database using BWA-MEM (v.0.7.15) and the number of primary alignments to each genome was used as input to DESeq2. *P* values for all comparisons were calculated by DESeq2 and adjusted for multiple testing^62^.

#### Rumen census analysis

The average and total depth for each genome in each dataset (Supplementary Data 5) was used as a proxy for abundance in the dataset(s). Kraken (as described above) was used with the rumen superset database to calculate the read abundance of Proteobacteria in all samples.

### Assembly and analysis of nanopore sequence data

The nanopore reads were extracted and quality controlled using poRe^63,64^ (v.0.24), and assembled using Canu^32^ (v.1.8) with default settings and genomeSize = 150 Mb. The resulting assembly was analyzed using MAGpy^10^. The raw assembly was corrected using both Nanopolish^65^ (v.0.10.2) and Racon^66^ (v.1.3.1) using Illumina data aligned to the nanopore assembly with Minimap2 (v.2.12) using short-read settings (-x sr). Query versus subject length data were extracted and plotted using IDEEL (https://github.com/mw55309/ideel). Whole-genome alignments were calculated using MUMmer79 (v.3.23) using promer to find matches between genomes. The three complete nanopore bacterial genomes and their Illumina counterparts were annotated using Prokka^67^ (v.1.13.3). The nanopore assembly was created with a minimum contig length of 1 kb; therefore, the Illumina assemblies were similarly limited before comparison.

#### Proteome analysis

Proteins were predicted using Prodigal (v.2.6.3) with option ‘-p meta’. Using DIAMOND, each protein was searched against KEGG (downloaded on 15 September 2018), UniRef100, UniRef90 and UniRef50 (downloaded 3 October 2018), and CAZy (dbCAN2 version, 31 July 2018). The protein predictions were clustered by CD-HIT^68^ (v.4.7) at 100%, 90% and 50% identity, mirroring similar methods at UniRef.

All protein predictions were searched against the CAZy database using dbCAN2 (ref. ^40^) and HMMER^69^ (v.3.1b2), and PULs were predicted for Bacteroidetes RUGs using PULpy^43^.

### Reporting Summary

Further information on research design is available in the Nature Research Reporting Summary linked to this article.

## Online content

Any methods, additional references, Nature Research reporting summaries, source data, statements of code and data availability and associated accession codes are available at 10.1038/s41587-019-0202-3.

## Supplementary Information

### Integrated supplementary information

Supplementary Figure 1 Quality of metagenome-assembled genomes. **a**) Completeness and contamination statistics for 4941 RUGs. Red points indicate the highest quality genomes with >=90% completeness and <=5% contamination. All other RUGs are >80% complete and <=10% contaminated. Those in blue have a quality score >=50 as defined by Parks *et al*, whereas those in grey have a quality score <= 50. **b**) Histogram of N50 for 4941 RUGs (log10 scale). **c**) Histogram of the number of contigs per genome for 4941 RUGs.

Supplementary Figure 2 *Proteobacteria* MAG whole-genome alignments. Whole genome alignments between the single-contig Illumina assembly RUG14498 (the x-axis on all plots) and nine similarly sized *Proteobacteria* MAGs from Parks *et al*. Clear, linear whole-genome alignments between RUG14498 and six of the Parks *et al* MAGs can be seen, with faint linear alignments distinguishable on a further two. UBA3307 and UBA1908 appear to include additional sequence with no orthologous matches in RUG14498.

Supplementary Figure 3 Comparison of rumen microbial genome datasets. A comparison of the various rumen MAG datasets after de-replication at 95% ANI. Members within each group are determined to be the same species as they share >= 95% ANI. Bottom left panel shows the size of each set; the bottom-middle panel shows the sets included in the intersection, and the top barplot shows the size of that intersection (note an intersection can include only one set). As can be seen, the sets largely represent independent species, with the first four largest intersections containing genomes from only one set. The largest overlap is between the Hungate collection and the RUGS, that is 58 species level bins contain both RUG and Hungate genomes. The RUG collection is the only set to contain overlaps with all other collections.

Supplementary Figure 4 Read classification rates. Classification rate for five datasets against various Kraken databases. BFAP bacterial, archaeal, fungal and protozoan genomes from RefSeq plus the Hungate collection; +RUG2 is BFAP plus the 4941 RUGs described in this manuscript; +RSS is BFAP plus the rumen superset (including the RUGs, UBA genomes and MAGs from Solden *et al* and Svartström *et al*) The classification rate is increased by using either the RUG or rumen superset databases, though the rumen superset achieves only a marginal increase in most cases; the exception is the Svartström *et al* moose data, where addition of their own MAGs increases classification rates considerably. Using the RUG database brings the average classification rate in our own data to 70.1%, and around 50% in the Shi et al and Rubino et al datasets. Sample sizes: Stewart (n=283 animals), Wallace (n=8 animals), Rubino (n=14 animals), Shi (n=16 animals), Svartström (n=6 animals). Centre line shows the median value; box shows the interquartile range; whiskers extend to the most extreme data point which is no more than 1.5 times the interquartile range from the box.

Supplementary Figure 5 Phylum-level PCA. Principal component analysis of phylum-level abundances comparing low (n=8 animals) and high (n=8 animals) methane emitting sheep.

Supplementary Figure 6 Family-level PCA. Principal component analysis of family-level abundances comparing low (n=8 animals) and high (n=8 animals) methane emitting sheep.

Supplementary Figure 7 Genus-level PCA. Principal component analysis of genus-level abundances comparing low (n=8 animals) and high (n=8 animals) methane emitting sheep.

Supplementary Figure 8 Species-level PCA. Principal component analysis of species-level proportional read-count data from Kraken, comparing low (n=8 animals) and high (n=8 animals) methane emitting sheep.

Supplementary Figure 9 Species-level heatmap. Heatmap of species-level abundances comparing low (n=8 animals) and high (n=8 animals) methane emitting sheep. The colour scheme transitions from navy (low values) through white (medium values) to dark red (high values).

Supplementary Figure 10 Strain-level PCA. Principal component analysis plot of the abundance of rumen superset genomes from low (n=8 animals) and high (n=8 animals) methane emitting sheep.

Supplementary Figure 11 Rumen *Proteobacteria* abundance. Boxplot of percentage abundance of *Proteobacteria* across 5 rumen metagenomic datasets. Y-axis is on a log10 scale. Sample sizes: Stewart (n=283 animals), Wallace (n=8 animals), Rubino (n=14 animals), Shi (n=16 animals), Svartström (n=6 animals). Centre line shows the median value; box shows the interquartile range; whiskers extend to the most extreme data point which is no more than 1.5 times the interquartile range from the box.

Supplementary Figure 12 Correcting errors in the MinION nanopore assembly. Histograms of predicted protein length vs length of the top hit in UniProt. Perfect predictions should show a tight distribution around 1. A) the raw assembly from Canu; B) after one round of Nanopolish; C) after one round of Nanopolish and one round of Racon; D) after one round of Nanopolish and two rounds of Racon; E) after one round of Nanopolish, two rounds of Racon, and a further round of Nanopolish; F) data from the 4941 RUGs; G) data from the 4941 RUGs with a limited y-axis, to highlight the long tail of short proteins remaining. the first round of Nanopolish produces a notable improvement, and the first round of Racon (with Illumina data) produces a drastic improvement. A second round of Racon (with Illumina data) produces a very slight improvement, and a final round of Nanopolish makes things slightly worse.

Supplementary Figure 13 *Prevotella copri* whole-genome alignments. Whole genome alignments between *Prevotella copri* nRUG14950 (tig00000032), RUG14032 and *Prevotella copri* DSM18205. RUG14032 and *Prevotella copri* DSM18205 exist as unordered contigs whereas *Prevotella copri* nRUG14950 (tig00000032) is a single contig assembled from Nanopore data.

Supplementary Figure 14 *Selenomonas ruminantium* whole-genome alignments. Whole genome alignments between *Selenomonas* spp. nRUG14951 (tig00000052), RUG110160 and *Selenomonas ruminantium* GACV-9. RUG110160 and *Selenomonas ruminantium* GACV-9 exist as unordered contigs whereas *Selenomonas* spp nRUG14951 (tig00000052) is a single contig assembled from Nanopore data.

Supplementary Figure 15 *Lachnospiraceae bacterium* whole-genome alignments. Whole genome alignments between *Lachnospiraceae bacterium* nRUG14952 (tig00000085), RUG13141 and *Lachnospiraceae bacterium* KHCPX20. RUG13141 and *Lachnospiraceae bacterium* KHCPX20 exist as unordered contigs whereas *Lachnospiraceae bacterium* nRUG14952 (tig00000085) is a single contig assembled from Nanopore data.

### Supplementary information

Supplementary Information Supplementary Figs. 1–15 and Supplementary Tables 1–5

Reporting Summary

Supplementary Data 1 Animal information. Breed, sex and age of the animals used in this study.

Supplementary Data 2 Assembly information. Completeness and contamination statistics for 4,941 RUGs with assembly statistics and basic taxonomic information.

Supplementary Data 3 Comparative genomic information. Comparative genomic information for the 4,941 RUGs, including sourmash comparison to GenBank genomes, DIAMOND results against UniProt, CheckM-predicted lineage, PhyloPhlAn-predicted lineage and GTDB-Tk-predicted lineage.

Supplementary Data 4 Phylogenetic tree. Phylogenetic tree for the 4,941 RUGs in Newick format, calculated using PhyloPhlAn.

Supplementary Data 5 RUG abundance data. The depth of each RUG in each sample, as calculated by MetaBAT2.

Supplementary Data 6 Species-level bins. Species-level bins for the rumen superset dereplicated at 95% ANI.

Supplementary Data 7 Strain-level bins. Strain-level bins for the rumen superset dereplicated at 99% ANI.

Supplementary Data 8 Differential abundance between LME and HME sheep. Differentially abundant genomes between LME (*n* = 8 animals) and HME (*n* = 8 animals) sheep, as calculated by DESeq2. baseMean is the average normalized counts across all samples; log2FoldChange is the log_2_ ratio of LME/HME; lfcSE gives the standard error of the log2FC; stat is the Wald statistic: the log2FC divided by lfcSE, which is compared to a standard normal distribution to generate a two-tailed *P* value; pvalue is the raw *P* value, and padj is the adjusted *P* value (Benjamini–Hochberg correction; also known as FDR).

Supplementary Data 9 Nanopore assembly statistics. Assembly statistics for the MinION nanopore assembly, for all contigs >500 kb or predicted to be circular. Includes DIAMOND protein comparisons to UniProt and to the Hungate collection.

Supplementary Data 10 Advantages of long-read assemblies. A comparison of each of the three long-read whole-chromosome assemblies with their most similar RUG, in terms of number of annotated full-length 16S sequences, number of annotated IS elements and number of annotates clusters of annotated genes.

## Data Availability

Raw sequence reads for all samples are available under European Nucleotide Archive (ENA) project PRJEB31266, except for 10572 that are available under PRJEB21624. All metagenomic assemblies and RUGs have been deposited in ENA under accession PRJEB31266. All protein predictions, clusters and annotation are available at 10.7488/ds/2470.

## Code availability

Comparative genomic analysis was carried out using MAGpy^10^ (https://github.com/WatsonLab/MAGpy); analysis of PULs was carried out using PULpy^43^ (https://github.com/WatsonLab/PULpy); and analysis of indels in nanopore data was carried out using IDEEL (https://github.com/mw55309/ideel).
