# Supplementary figures and images for: Compendium of 4,941 rumen metagenome-assembled genomes for rumen microbiome biology and enzyme discovery
Source: Nat Biotechnol. 2019 Aug 2;37(8):953–61. doi: 10.1038/s41587-019-0202-3 (PMC6785717; doi:10.1038/s41587-019-0202-3)

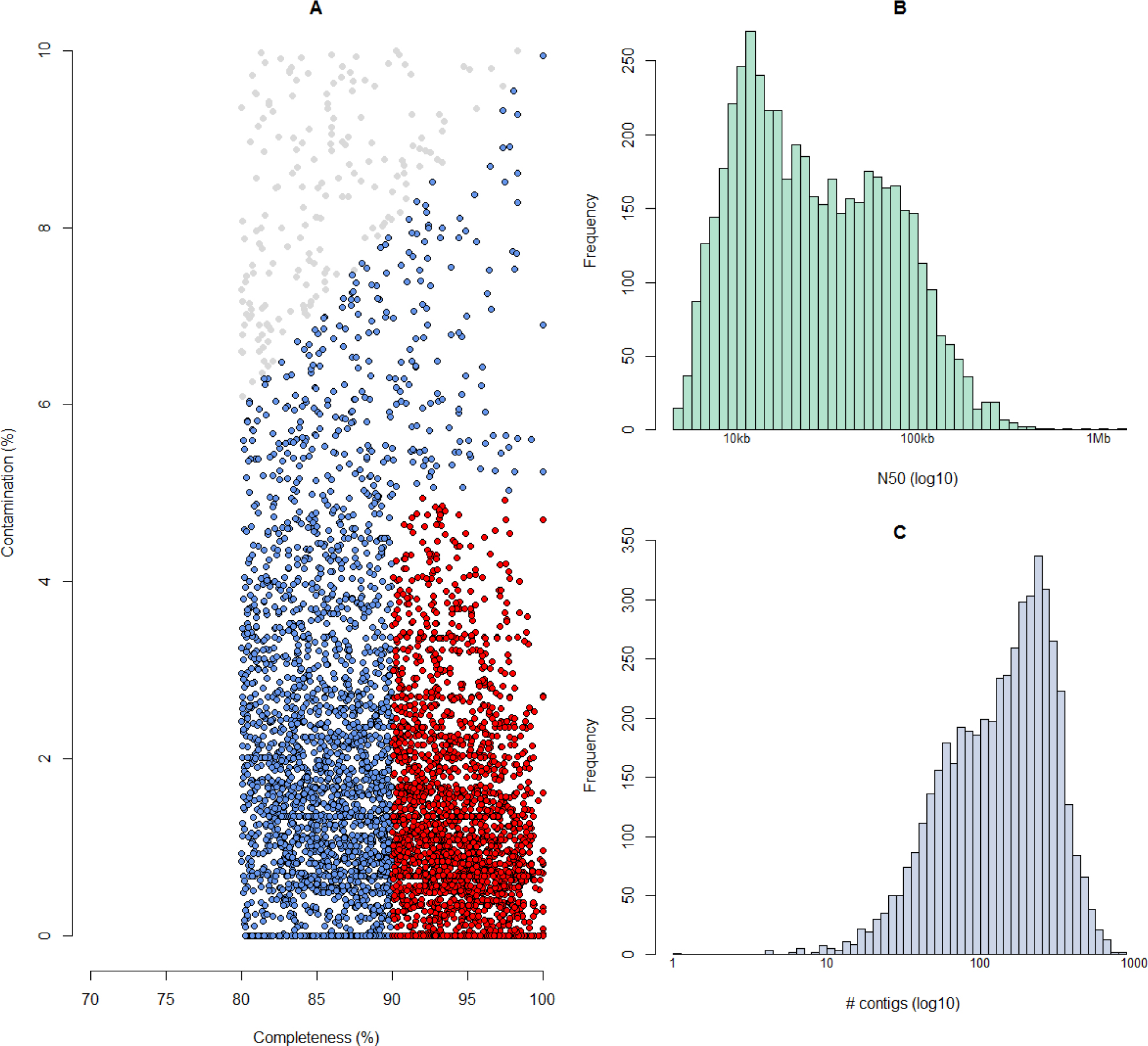

Supplement: Quality of metagenome-assembled genomes. — a) Completeness and contamination statistics for 4941 RUGs. Red points indicate the highest quality genomes with >=90% completeness and <=5% contamination. All other RUGs are >80% complete and <=10% contaminated. Those in blue have a quality score >=50 as defined by Parks et al, whereas those in grey have a quality score <= 50. b) Histogram of N50 for 4941 RUGs (log10 scale). c) Histogram of the number of contigs per genome for 4941 RUGs. [file 41587_2019_202_Fig6_ESM.jpg]

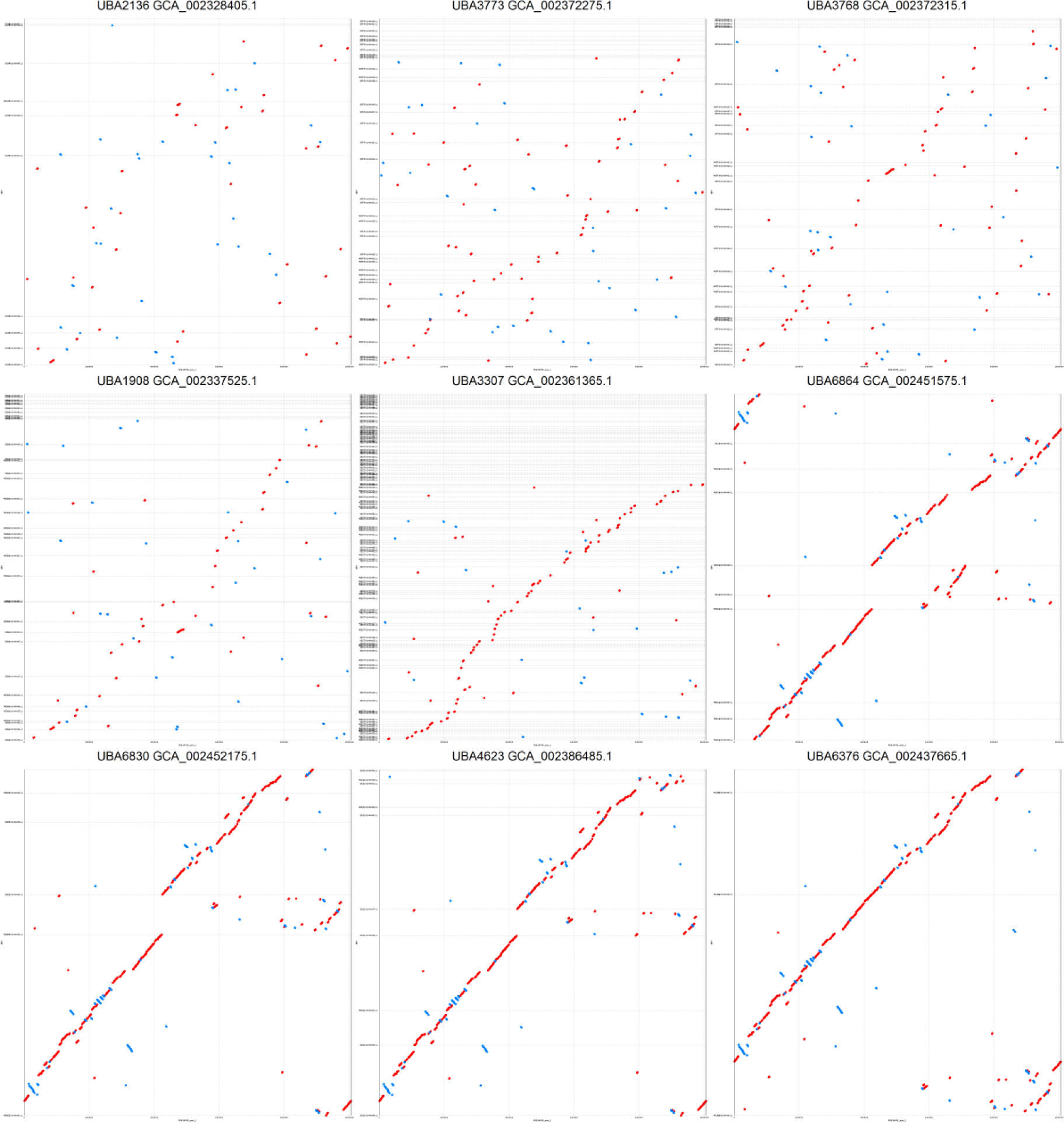

Supplement: Proteobacteria MAG whole-genome alignments. — Whole genome alignments between the single-contig Illumina assembly RUG14498 (the x-axis on all plots) and nine similarly sized Proteobacteria MAGs from Parks et al. Clear, linear whole-genome alignments between RUG14498 and six of the Parks et al MAGs can be seen, with faint linear alignments distinguishable on a further two. UBA3307 and UBA1908 appear to include additional sequence with no orthologous matches in RUG14498. [file 41587_2019_202_Fig7_ESM.jpg]

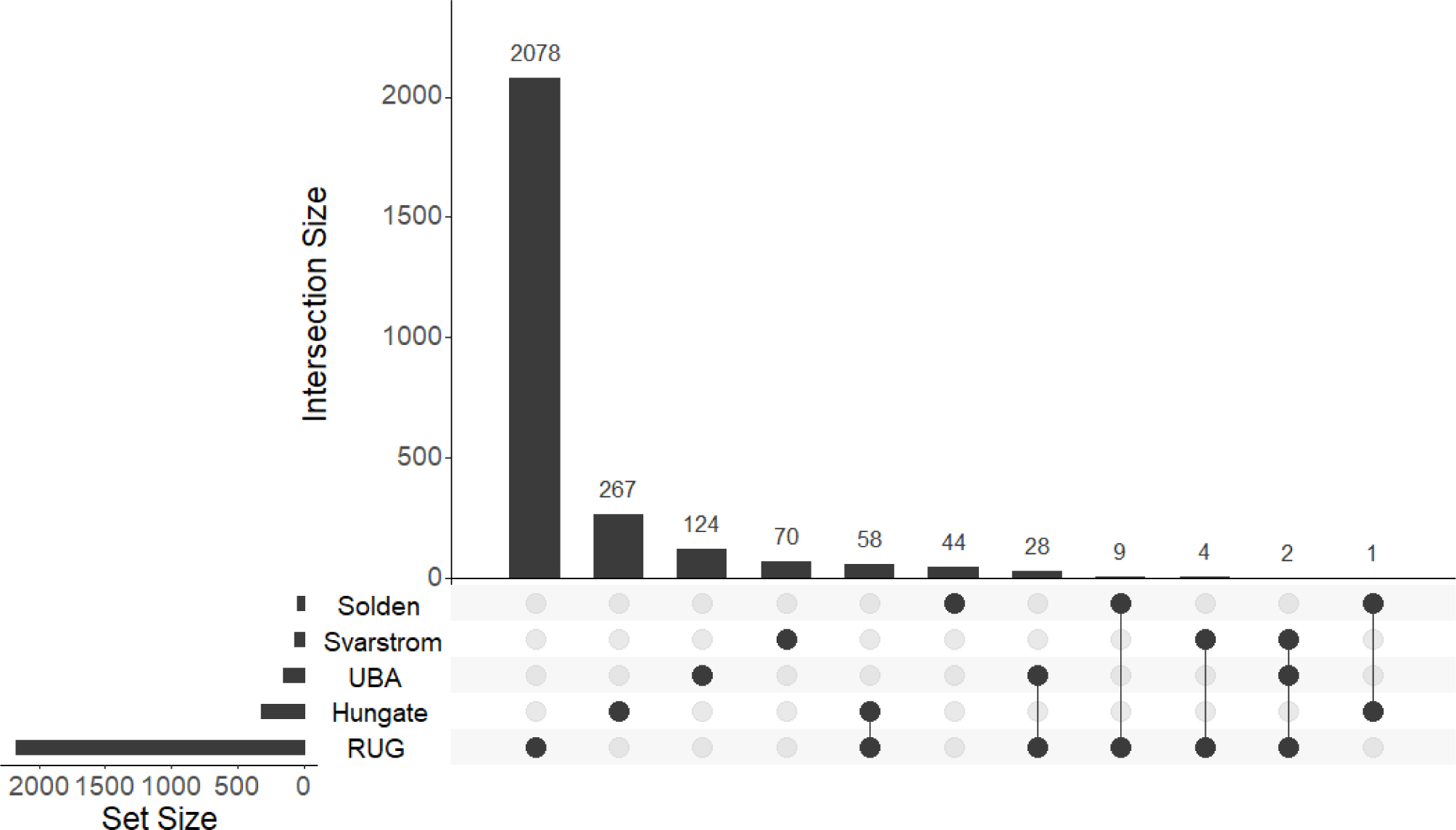

Supplement: Comparison of rumen microbial genome datasets. — A comparison of the various rumen MAG datasets after de-replication at 95% ANI. Members within each group are determined to be the same species as they share >= 95% ANI. Bottom left panel shows the size of each set; the bottom-middle panel shows the sets included in the intersection, and the top barplot shows the size of that intersection (note an intersection can include only one set). As can be seen, the sets largely represent independent species, with the first four largest intersections containing genomes from only one set. The largest overlap is between the Hungate collection and the RUGS, that is 58 species level bins contain both RUG and Hungate genomes. The RUG collection is the only set to contain overlaps with all other collections. [file 41587_2019_202_Fig8_ESM.jpg]

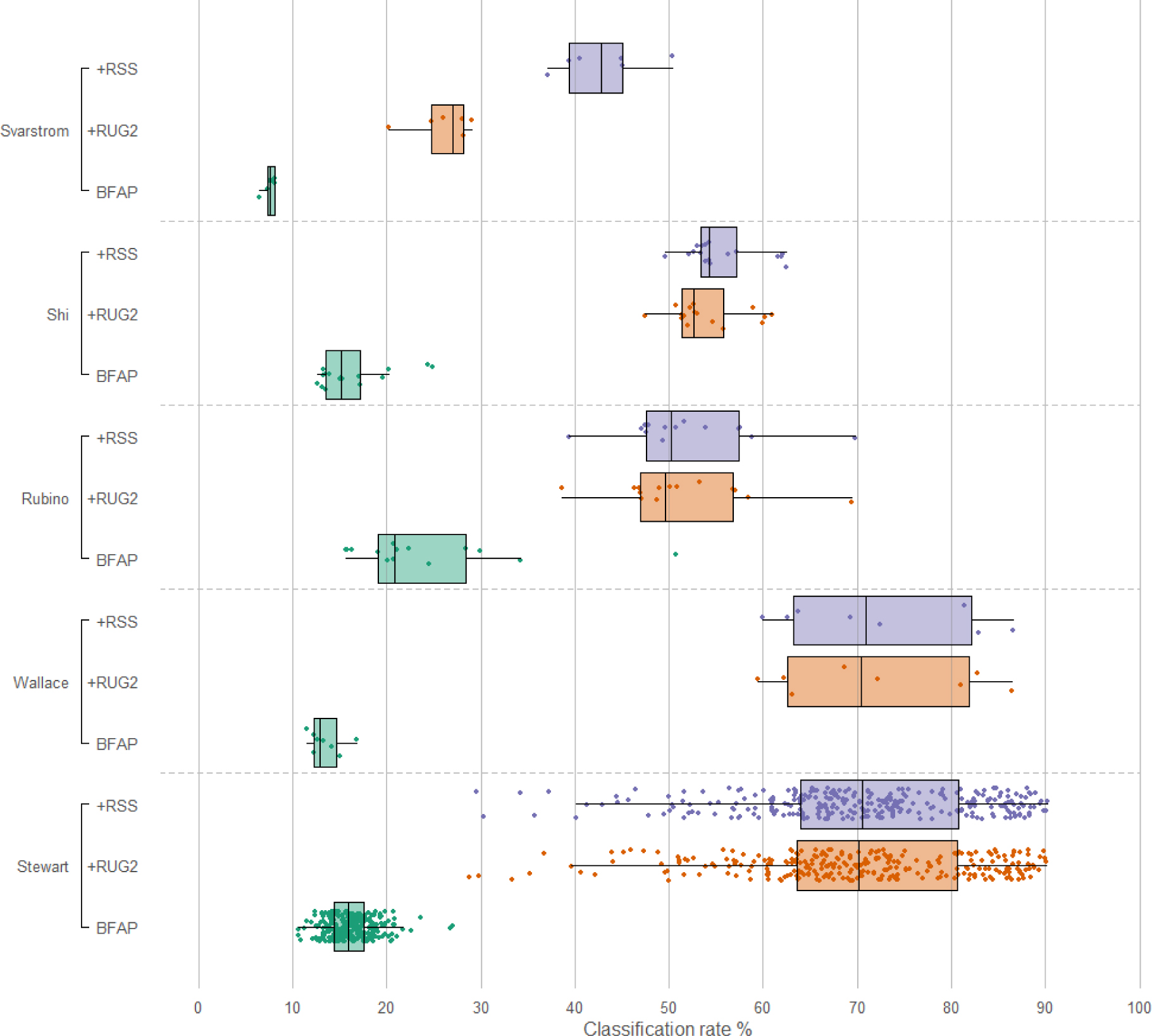

Supplement: Read classification rates. — Classification rate for five datasets against various Kraken databases. BFAP bacterial, archaeal, fungal and protozoan genomes from RefSeq plus the Hungate collection; +RUG2 is BFAP plus the 4941 RUGs described in this manuscript; +RSS is BFAP plus the rumen superset (including the RUGs, UBA genomes and MAGs from Solden et al and Svartström et al) The classification rate is increased by using either the RUG or rumen superset databases, though the rumen superset achieves only a marginal increase in most cases; the exception is the Svartström et al moose data, where addition of their own MAGs increases classification rates considerably. Using the RUG database brings the average classification rate in our own data to 70.1%, and around 50% in the Shi et al and Rubino et al datasets. Sample sizes: Stewart (n=283 animals), Wallace (n=8 animals), Rubino (n=14 animals), Shi (n=16 animals), Svartström (n=6 animals). Centre line shows the median value; box shows the interquartile range; whiskers extend to the most extreme data point which is no more than 1.5 times the interquartile range from the box. [file 41587_2019_202_Fig9_ESM.jpg]

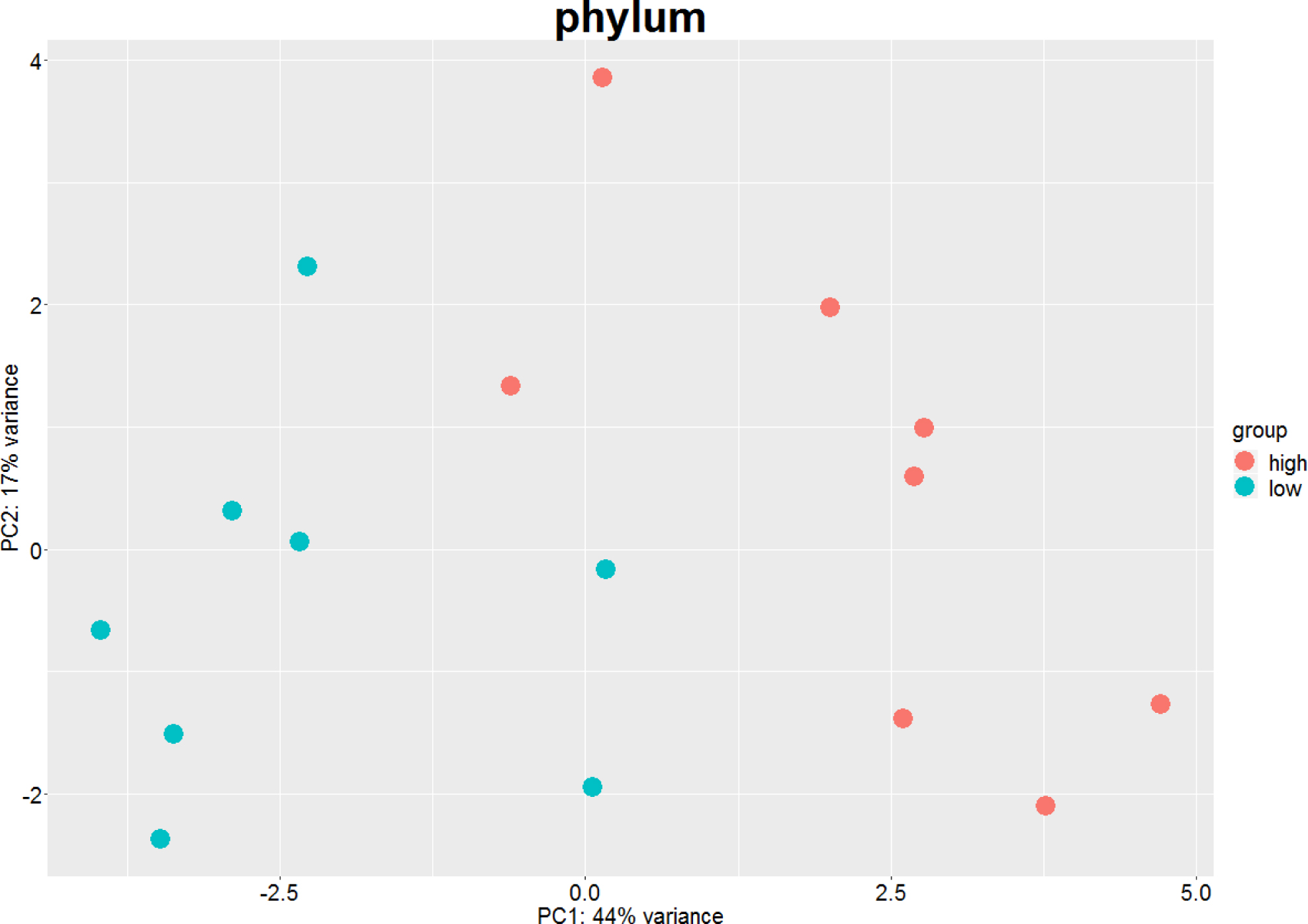

Supplement: Phylum-level PCA. — Principal component analysis of phylum-level abundances comparing low (n=8 animals) and high (n=8 animals) methane emitting sheep. [file 41587_2019_202_Fig10_ESM.jpg]

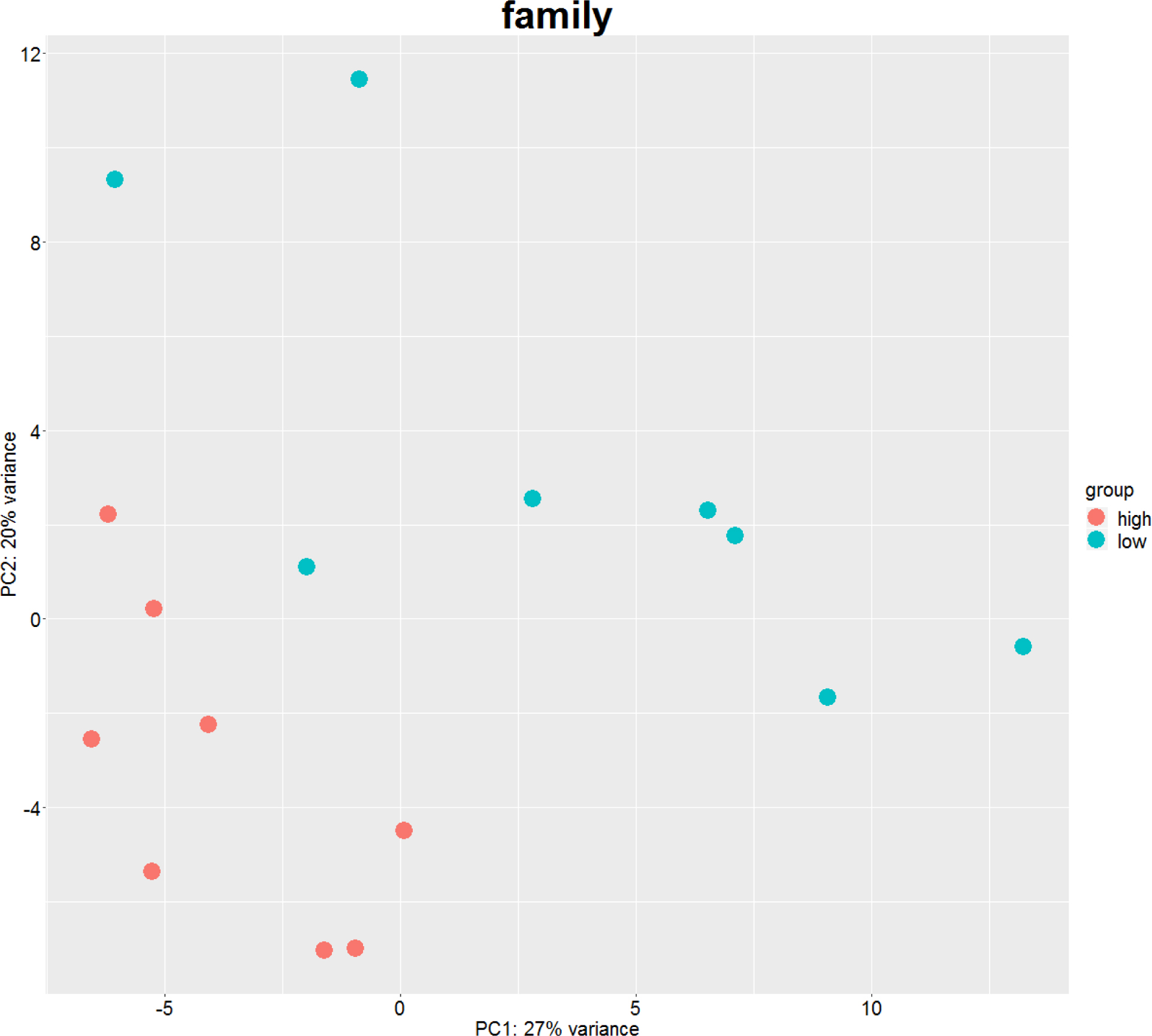

Supplement: Family-level PCA. — Principal component analysis of family-level abundances comparing low (n=8 animals) and high (n=8 animals) methane emitting sheep. [file 41587_2019_202_Fig11_ESM.jpg]

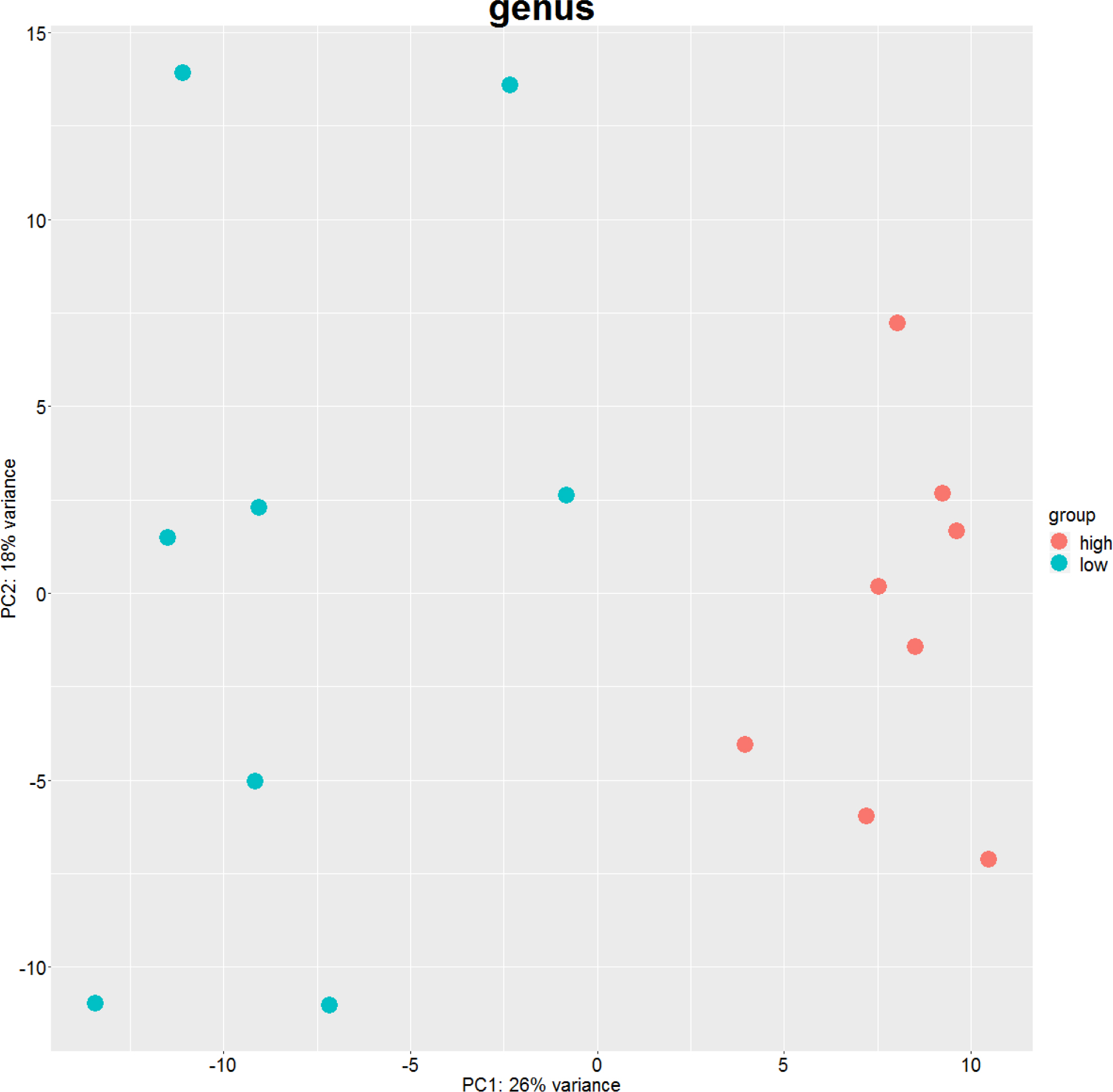

Supplement: Genus-level PCA. — Principal component analysis of genus-level abundances comparing low (n=8 animals) and high (n=8 animals) methane emitting sheep. [file 41587_2019_202_Fig12_ESM.jpg]

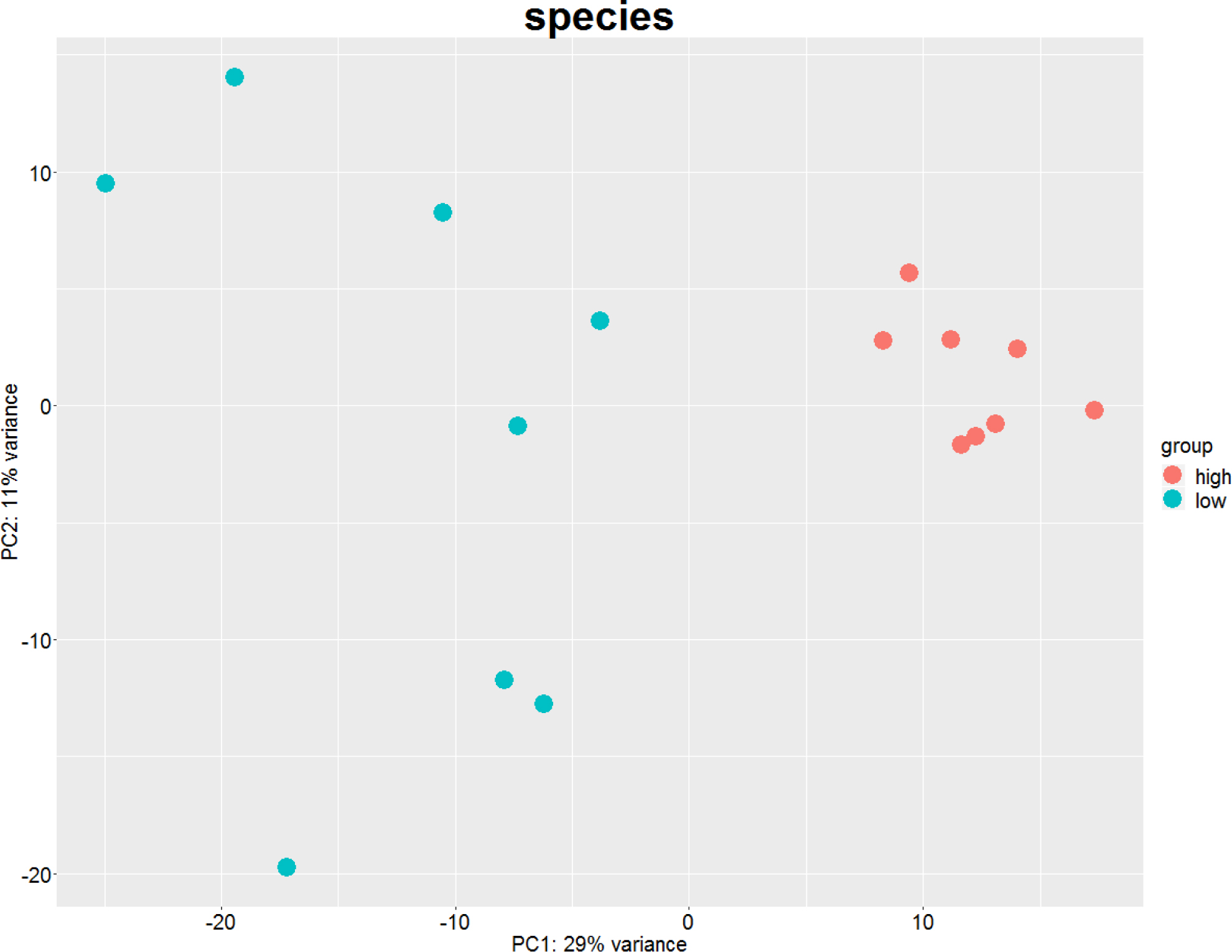

Supplement: Species-level PCA. — Principal component analysis of species-level proportional read-count data from Kraken, comparing low (n=8 animals) and high (n=8 animals) methane emitting sheep. [file 41587_2019_202_Fig13_ESM.jpg]

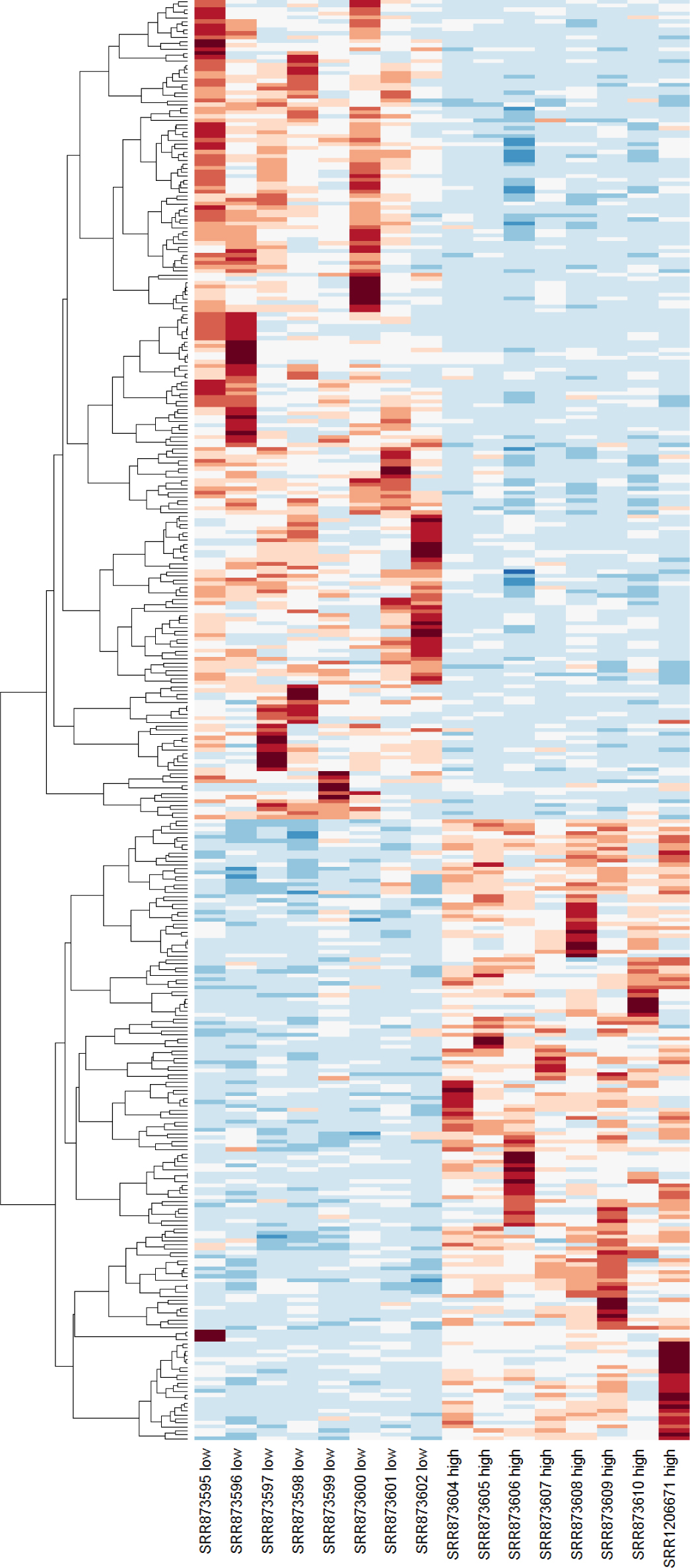

Supplement: Species-level heatmap. — Heatmap of species-level abundances comparing low (n=8 animals) and high (n=8 animals) methane emitting sheep. The colour scheme transitions from navy (low values) through white (medium values) to dark red (high values). [file 41587_2019_202_Fig14_ESM.jpg]

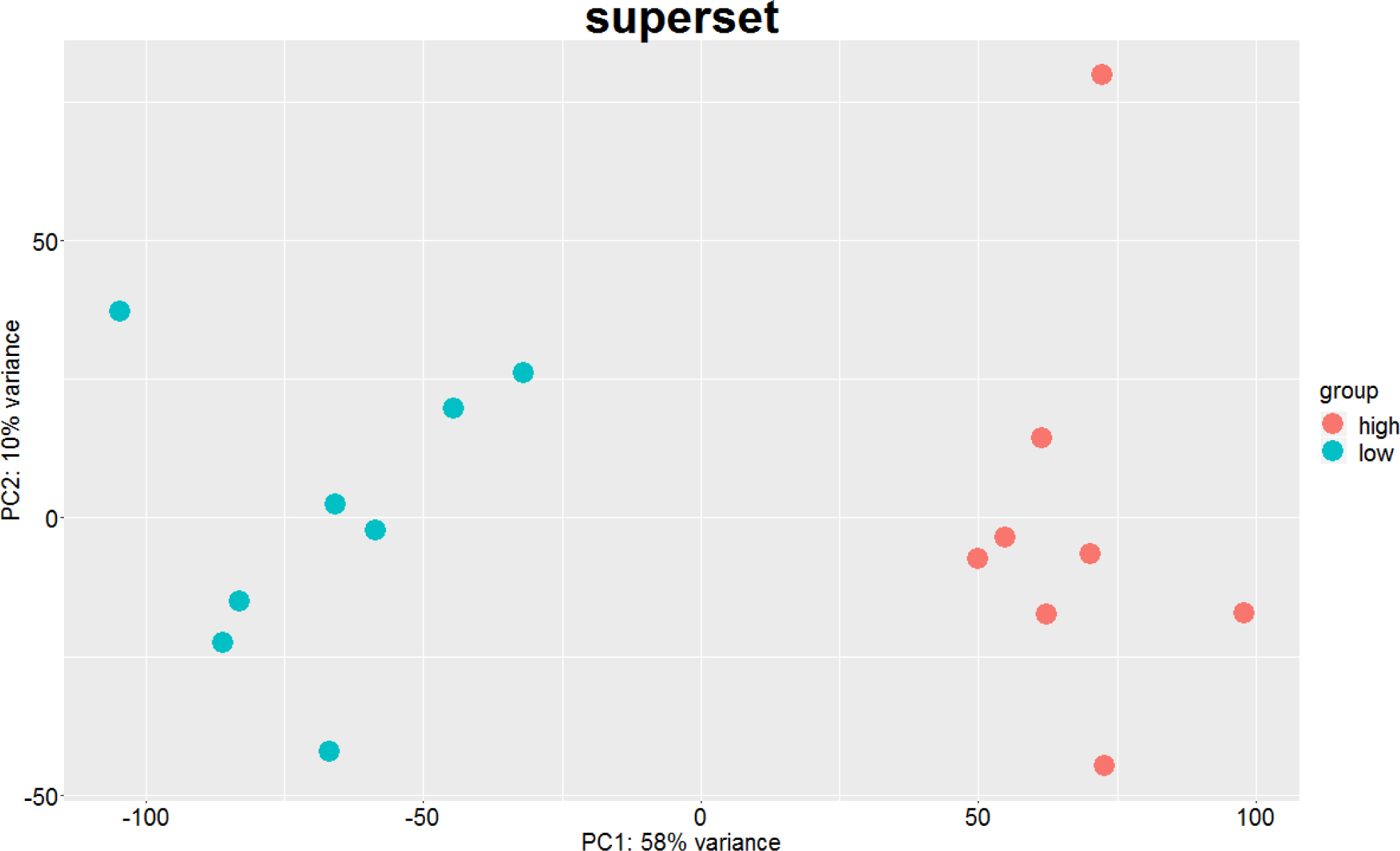

Supplement: Strain-level PCA. — Principal component analysis plot of the abundance of rumen superset genomes from low (n=8 animals) and high (n=8 animals) methane emitting sheep. [file 41587_2019_202_Fig15_ESM.jpg]

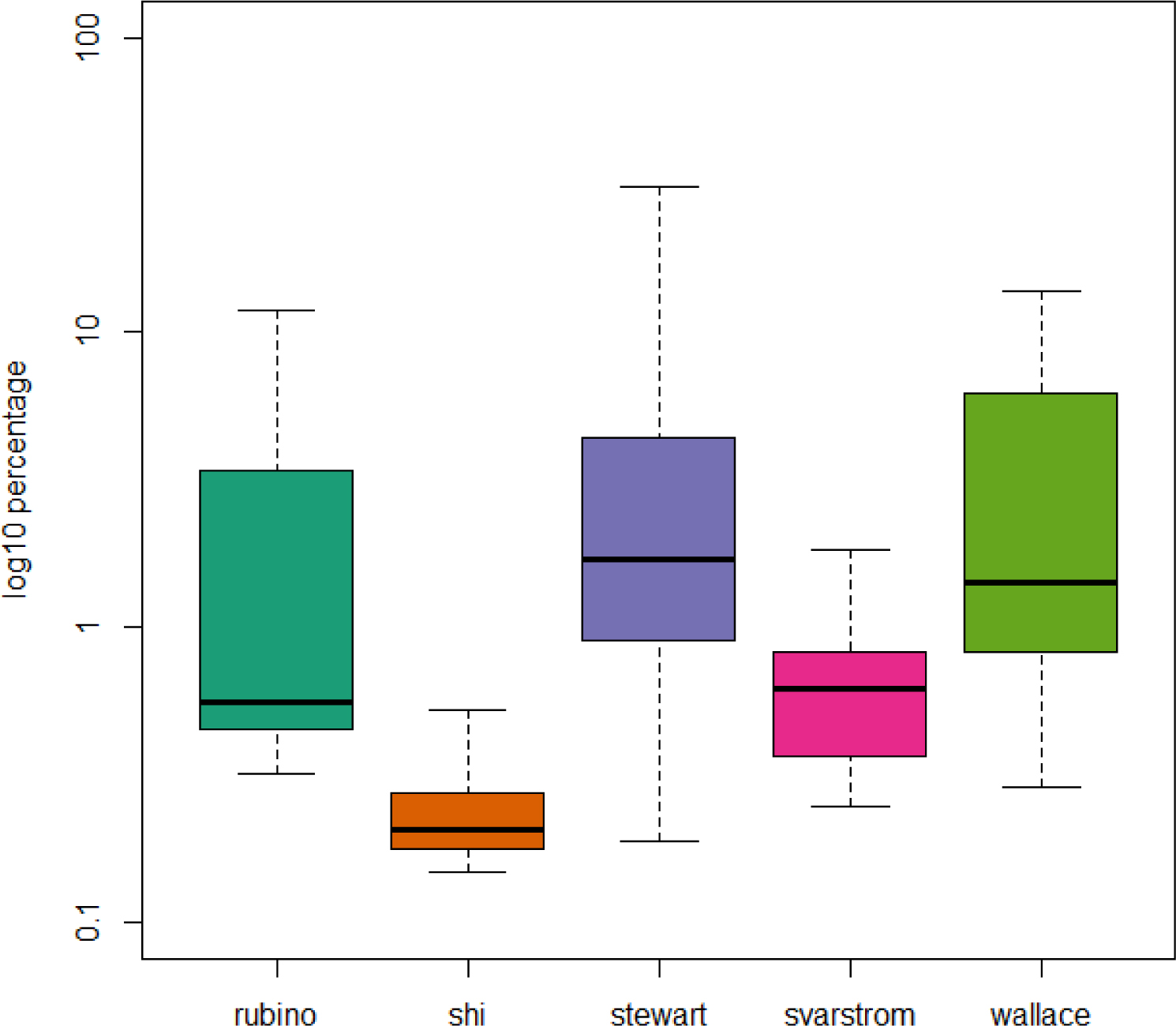

Supplement: Rumen Proteobacteria abundance. — Boxplot of percentage abundance of Proteobacteria across 5 rumen metagenomic datasets. Y-axis is on a log10 scale. Sample sizes: Stewart (n=283 animals), Wallace (n=8 animals), Rubino (n=14 animals), Shi (n=16 animals), Svartström (n=6 animals). Centre line shows the median value; box shows the interquartile range; whiskers extend to the most extreme data point which is no more than 1.5 times the interquartile range from the box. [file 41587_2019_202_Fig16_ESM.jpg]

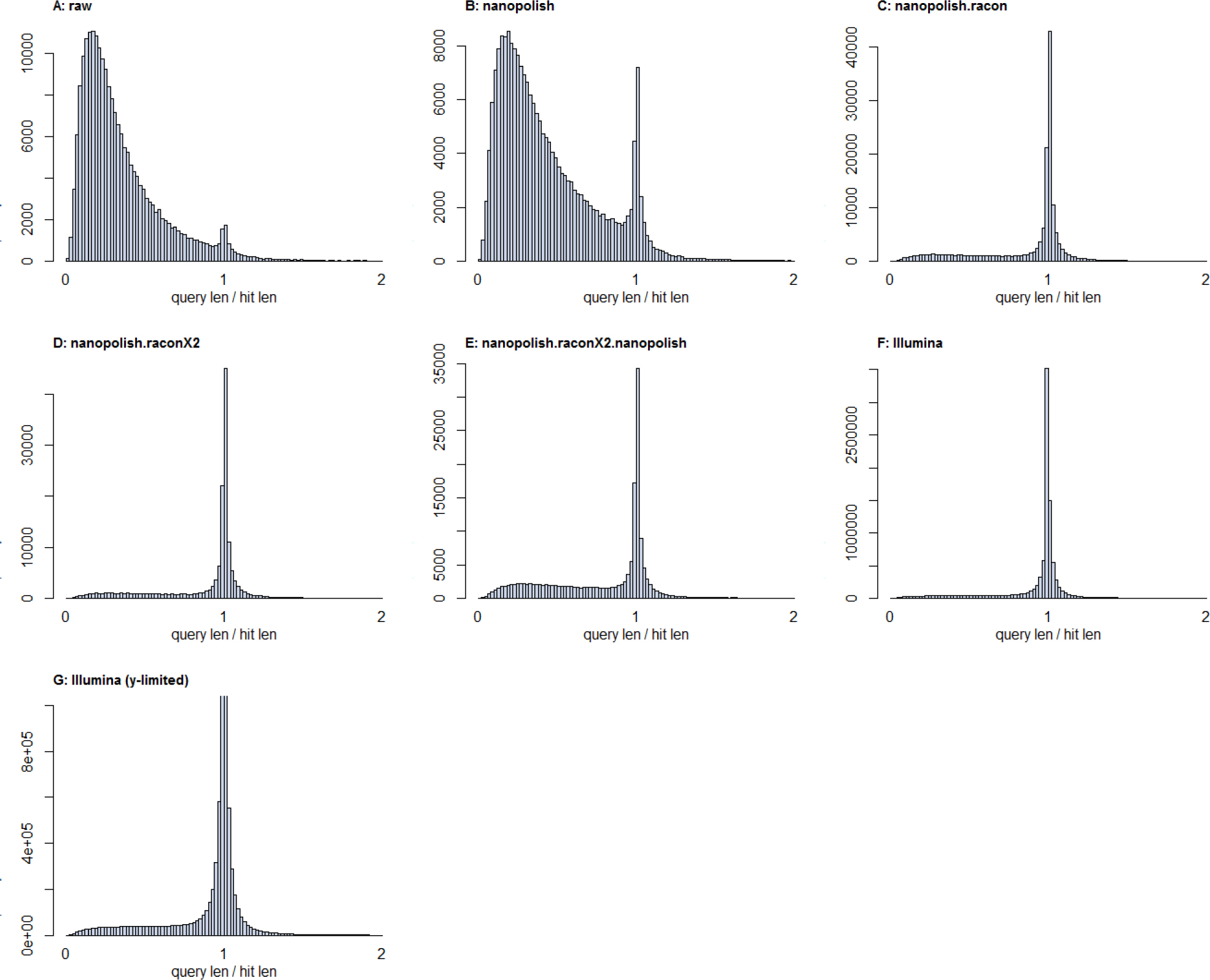

Supplement: Correcting errors in the MinION nanopore assembly. — Histograms of predicted protein length vs length of the top hit in UniProt. Perfect predictions should show a tight distribution around 1. A) the raw assembly from Canu; B) after one round of Nanopolish; C) after one round of Nanopolish and one round of Racon; D) after one round of Nanopolish and two rounds of Racon; E) after one round of Nanopolish, two rounds of Racon, and a further round of Nanopolish; F) data from the 4941 RUGs; G) data from the 4941 RUGs with a limited y-axis, to highlight the long tail of short proteins remaining. the first round of Nanopolish produces a notable improvement, and the first round of Racon (with Illumina data) produces a drastic improvement. A second round of Racon (with Illumina data) produces a very slight improvement, and a final round of Nanopolish makes things slightly worse. [file 41587_2019_202_Fig17_ESM.jpg]

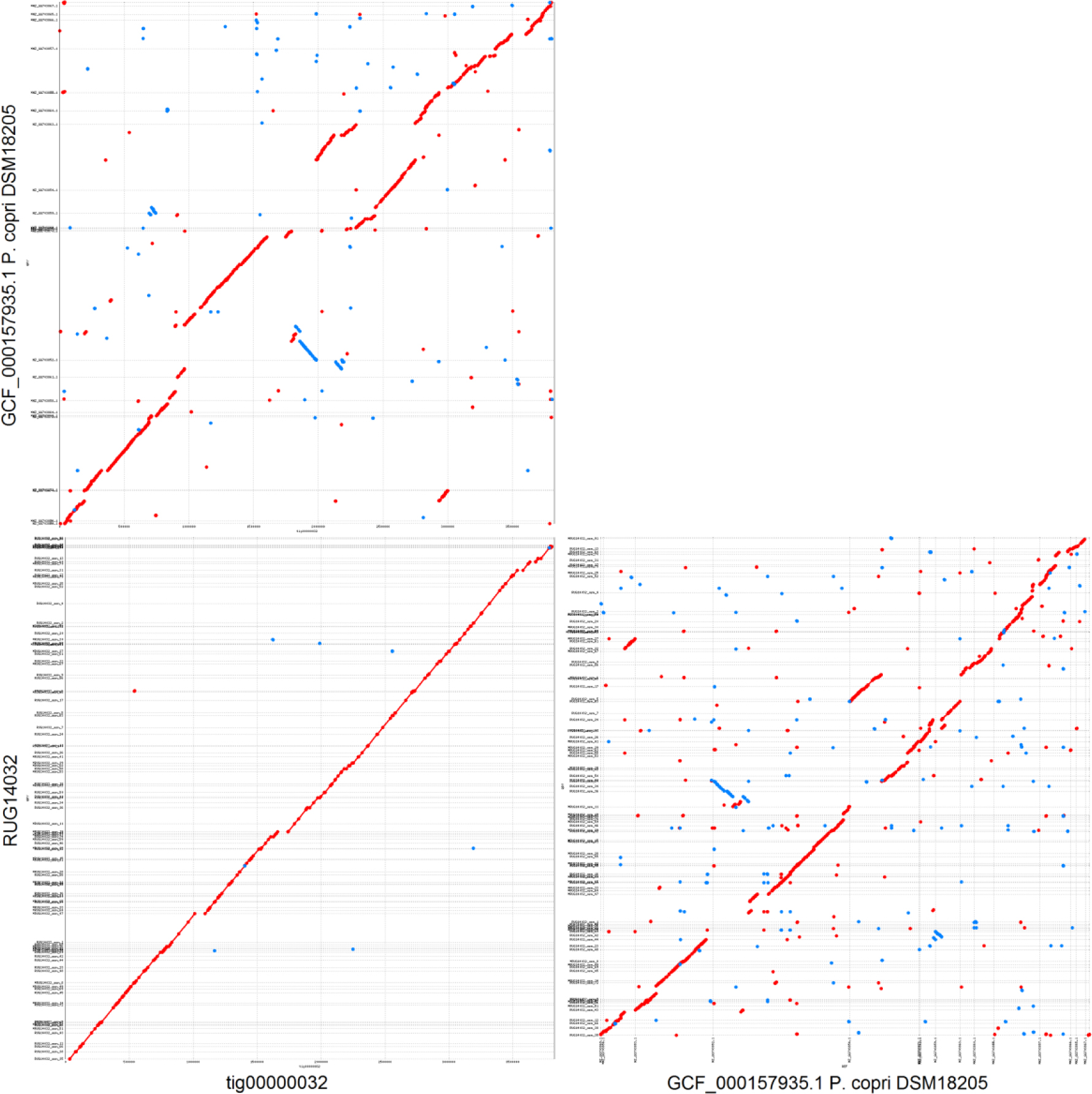

Supplement: Prevotella copri whole-genome alignments. — Whole genome alignments between Prevotella copri nRUG14950 (tig00000032), RUG14032 and Prevotella copri DSM18205. RUG14032 and Prevotella copri DSM18205 exist as unordered contigs whereas Prevotella copri nRUG14950 (tig00000032) is a single contig assembled from Nanopore data. [file 41587_2019_202_Fig18_ESM.jpg]

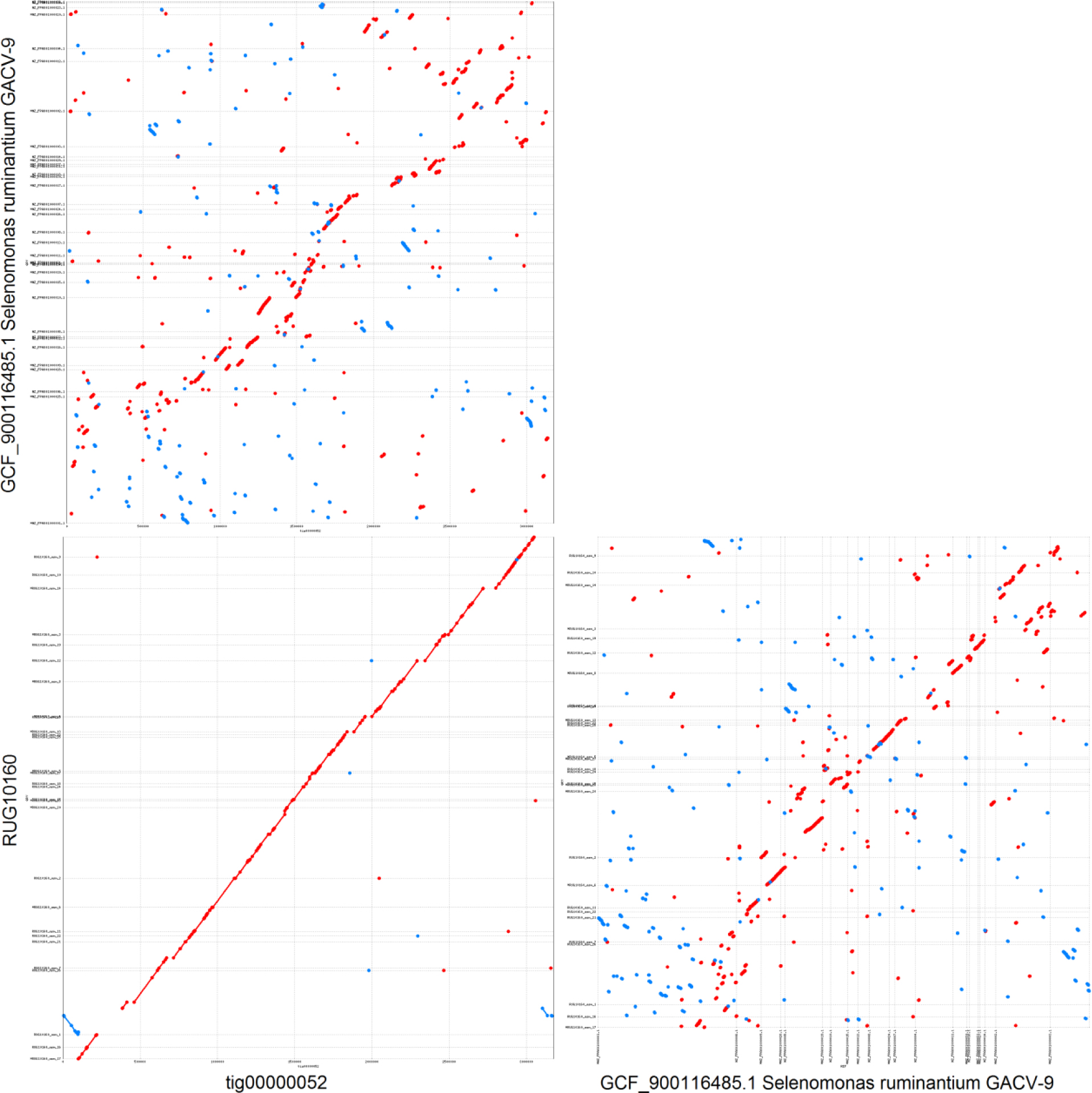

Supplement: Selenomonas ruminantium whole-genome alignments. — Whole genome alignments between Selenomonas spp. nRUG14951 (tig00000052), RUG110160 and Selenomonas ruminantium GACV-9. RUG110160 and Selenomonas ruminantium GACV-9 exist as unordered contigs whereas Selenomonas spp nRUG14951 (tig00000052) is a single contig assembled from Nanopore data. [file 41587_2019_202_Fig19_ESM.jpg]

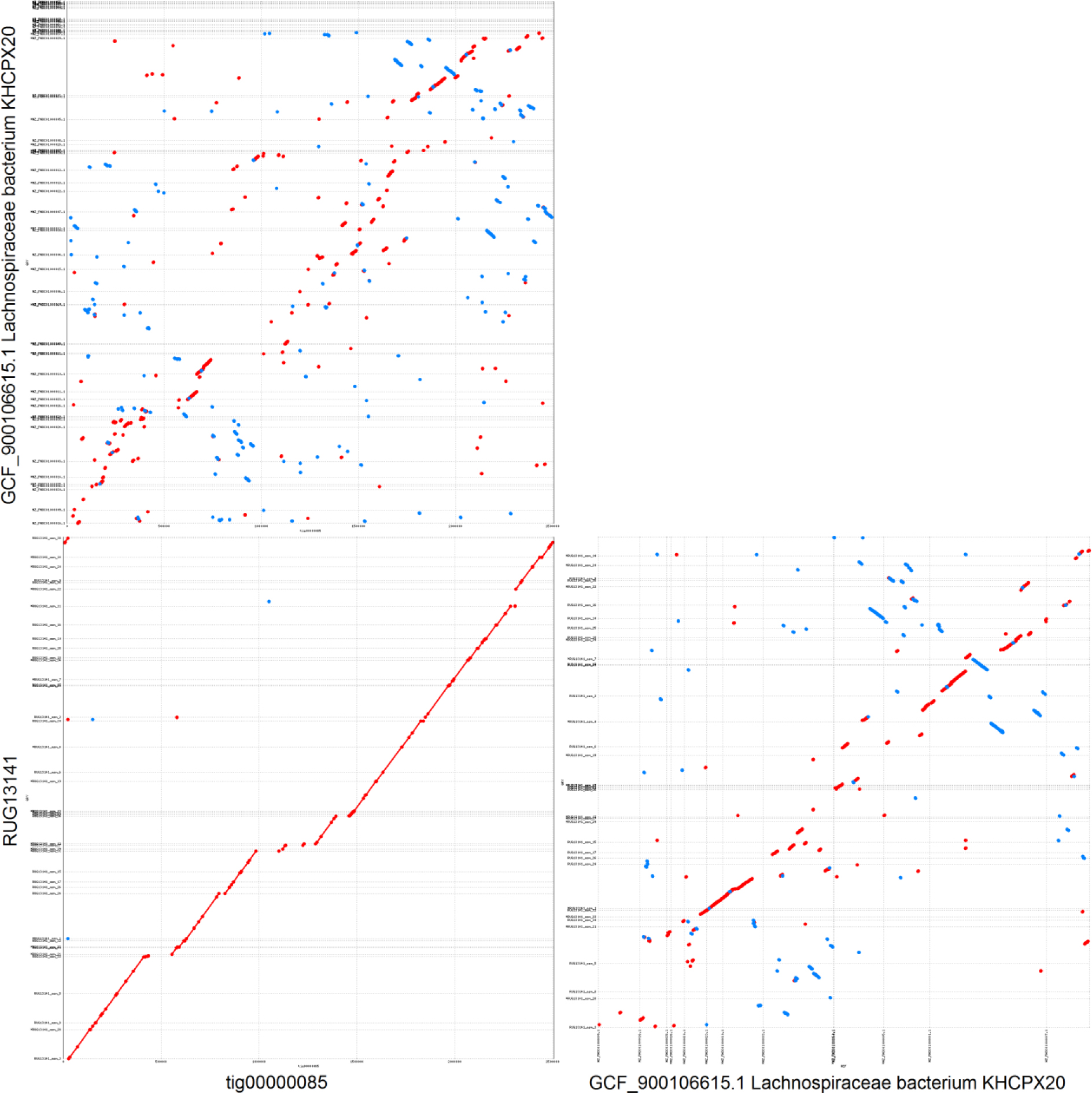

Supplement: Lachnospiraceae bacterium whole-genome alignments. — Whole genome alignments between Lachnospiraceae bacterium nRUG14952 (tig00000085), RUG13141 and Lachnospiraceae bacterium KHCPX20. RUG13141 and Lachnospiraceae bacterium KHCPX20 exist as unordered contigs whereas Lachnospiraceae bacterium nRUG14952 (tig00000085) is a single contig assembled from Nanopore data. [file 41587_2019_202_Fig20_ESM.jpg]
