# Supplementary material for: Compendium of 4,941 rumen metagenome-assembled genomes for rumen microbiome biology and enzyme discovery
Source: Nat Biotechnol. 2019 Aug 2;37(8):953–61. doi: 10.1038/s41587-019-0202-3 (PMC6785717; doi:10.1038/s41587-019-0202-3)
Supplement: Supplementary file 16 — Supplementary Figs. 1–15 and Supplementary Tables 1–5 [file 41587_2019_202_MOESM1_ESM.pdf]

nature biotechnology

SUPPLEMENTARY INFORMATION
https://doi.org/10.1038/s41587-019-0202-3

In the format provided by the authors and unedited.

Compendium of 4,941 rumen metagenome-assembled genomes for rumen microbiome biology and enzyme discovery

Robert D. Stewart[1], Marc D. Auffret[2], Amanda Warr[1], Alan W. Walker[3], Rainer Roehe[2] and Mick Watson[1*]

[1]The Roslin Institute and the Royal (Dick) School of Veterinary Studies, University of Edinburgh, Easter Bush, UK. [2]Scotland's Rural College, Edinburgh, UK. [3]The Rowett Institute, University of Aberdeen, Aberdeen, UK. *e-mail: mick.watson@roslin.ed.ac.uk

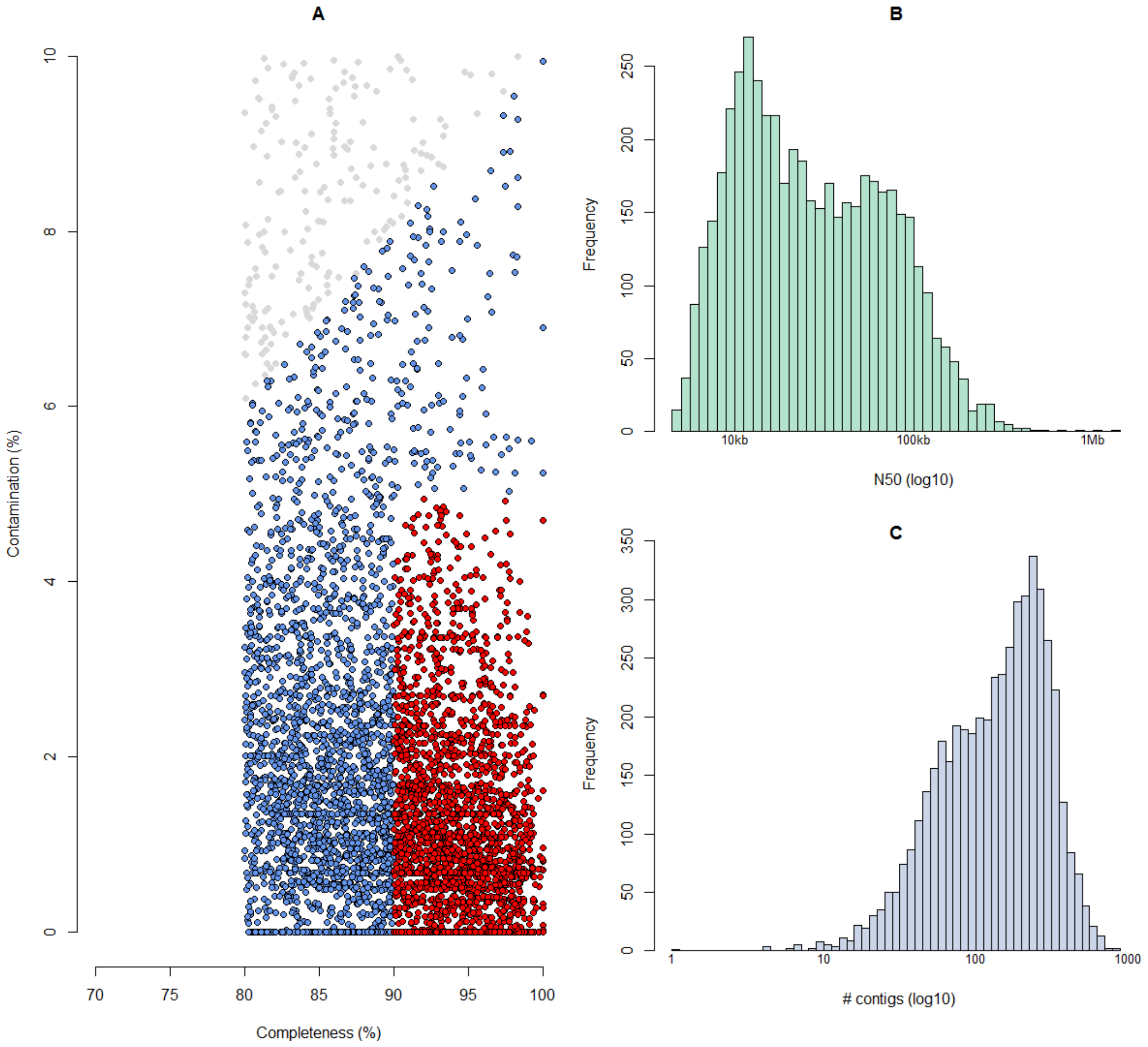

Supplementary Figure 1

Quality of metagenome-assembled genomes

A) Completeness and contamination statistics for 4941 RUGs. Red points indicate the highest quality genomes with >=90% completeness and <=5% contamination. All other RUGs are >80% complete and <=10% contaminated. Those in blue have a quality score >=50 as defined by Parks *et al*, whereas those in grey have a quality score <= 50. B) Histogram of N50 for 4941 RUGs (log10 scale). C) Histogram of the number of contigs per genome for 4941 RUGs.

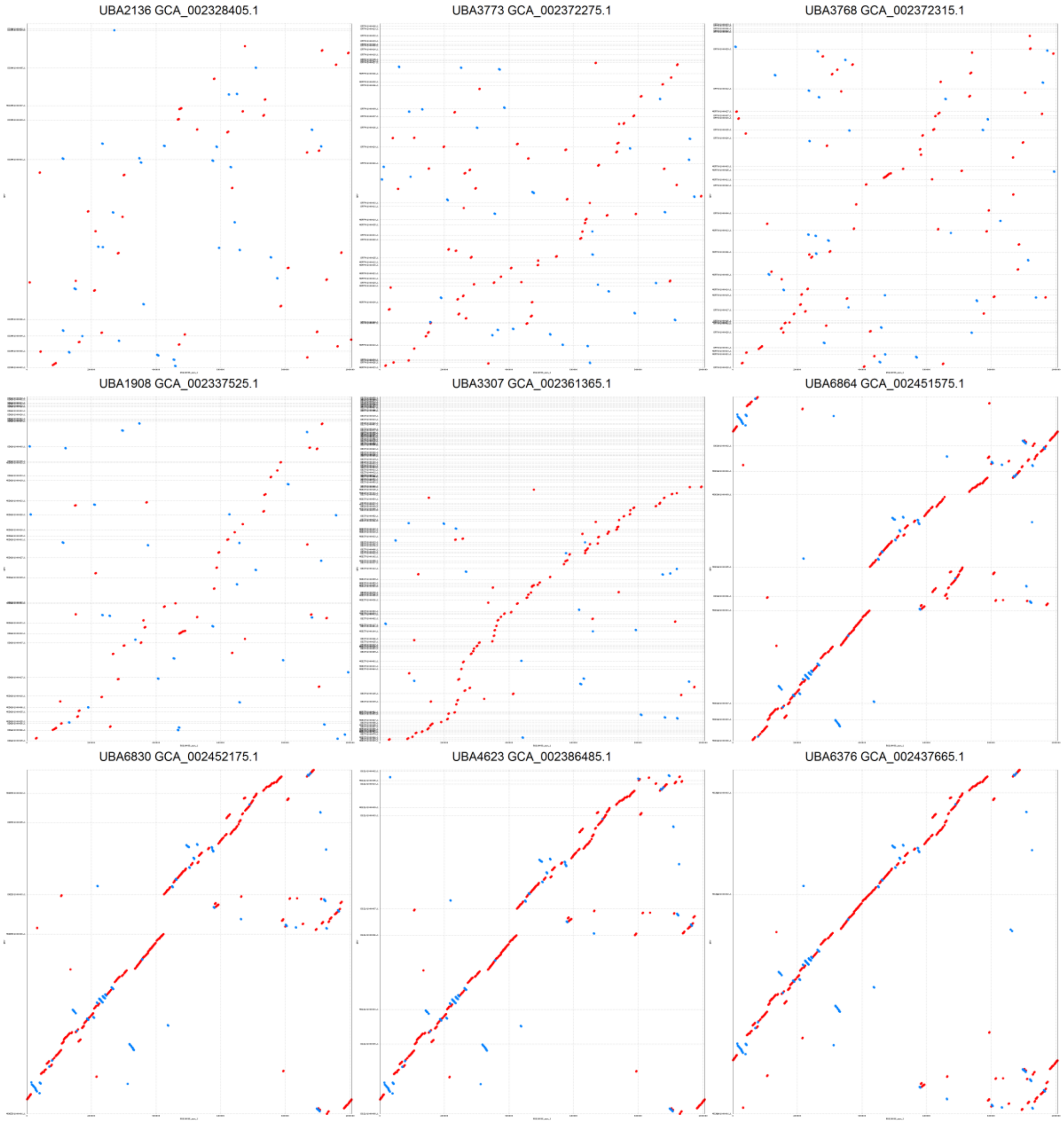

Supplementary Figure 2

Proteobacteria MAG whole-genome alignments

Whole genome alignments between the single-contig Illumina assembly RUG14498 (the x-axis on all plots) and nine similarly sized *Proteobacteria* MAGs from Parks *et al*. Clear, linear whole-genome alignments between RUG14498 and six of the Parks *et al* MAGs can be seen, with faint linear alignments distinguishable on a further two. UBA3307 and UBA1908 appear to include additional sequence with no orthologous matches in RUG14498.

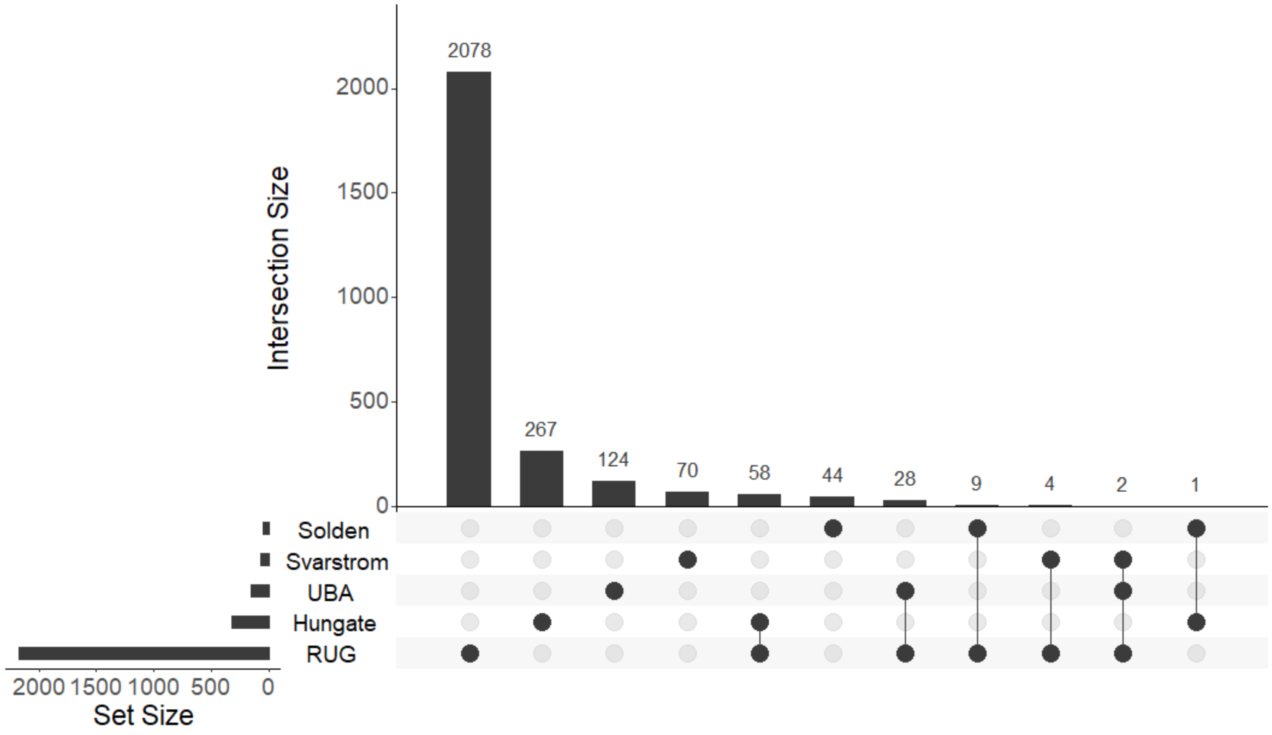

Supplementary Figure 3

Comparison of rumen microbial genome datasets

A comparison of the various rumen MAG datasets after de-replication at 95% ANI. Members within each group are determined to be the same species as they share >= 95% ANI. Bottom left panel shows the size of each set; the bottom-middle panel shows the sets included in the intersection, and the top barplot shows the size of that intersection (note an intersection can include only one set). As can be seen, the sets largely represent independent species, with the first four largest intersections containing genomes from only one set. The largest overlap is between the Hungate collection and the RUGS, i.e. 58 species level bins contain both RUG and Hungate genomes. The RUG collection is the only set to contain overlaps with all other collections.

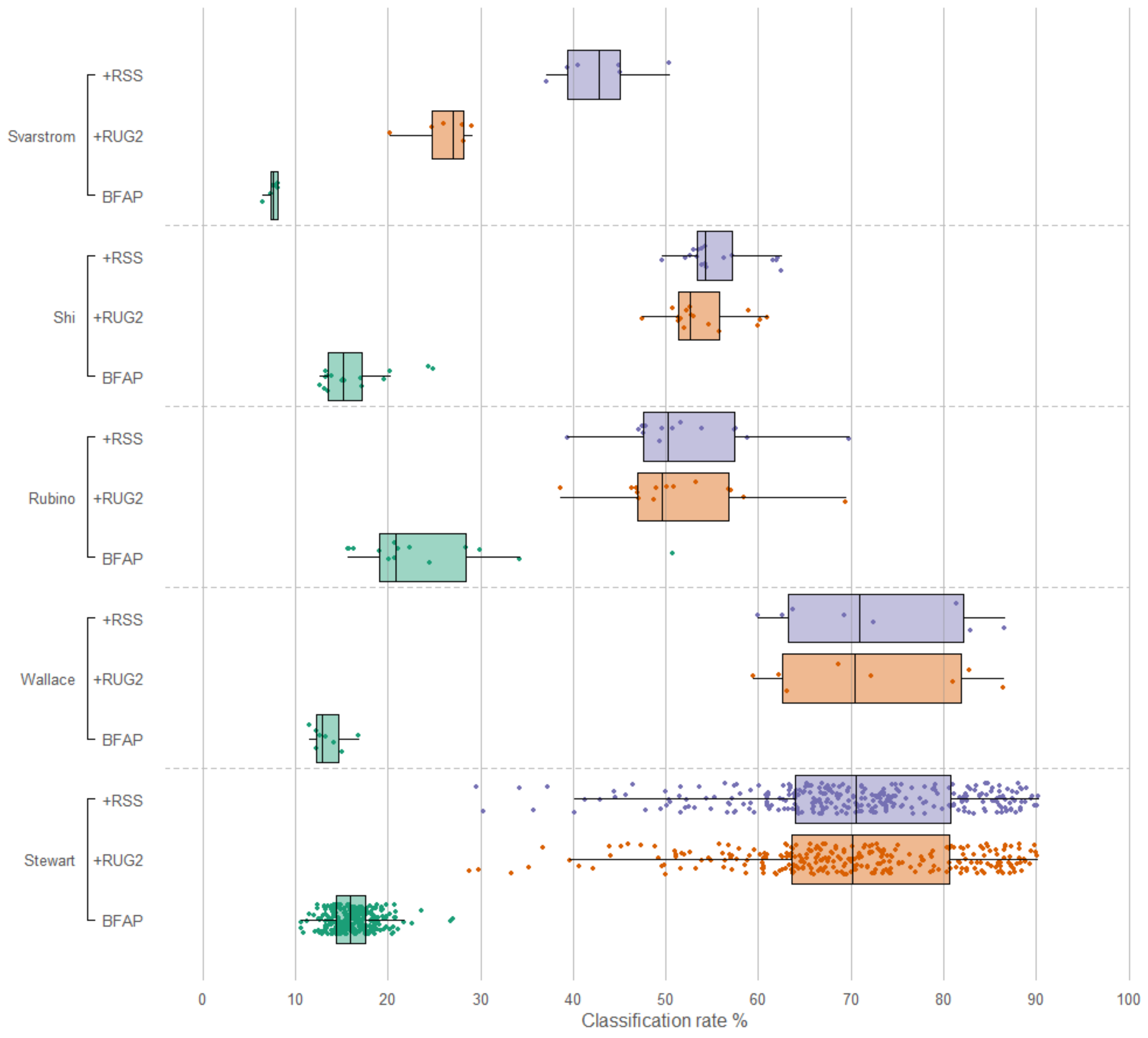

Supplementary Figure 4

Read classification rates

Classification rate for five datasets against various Kraken databases. BFAP bacterial, archaeal, fungal and protozoan genomes from RefSeq plus the Hungate collection; +RUG2 is BFAP plus the 4941 RUGs described in this manuscript; +RSS is BFAP plus the rumen superset (including the RUGs, UBA genomes and MAGs from Solden *et al* and Svartström *et al*) The classification rate is increased by using either the RUG or rumen superset databases, though the rumen superset achieves only a marginal increase in most cases; the exception is the Svartström *et al* moose data, where addition of their own MAGs increases classification rates considerably. Using the RUG database brings the average classification rate in our own data to 70.1%, and around 50% in the Shi *et al* and Rubino *et al* datasets. Sample sizes: Stewart (n=283 animals), Wallace (n=8 animals), Rubino (n=14 animals), Shi (n=16 animals), Svartström (n=6 animals). Centre line shows the median value; box shows the interquartile range; whiskers extend to the most extreme data point which is no more than 1.5 times the interquartile range from the box

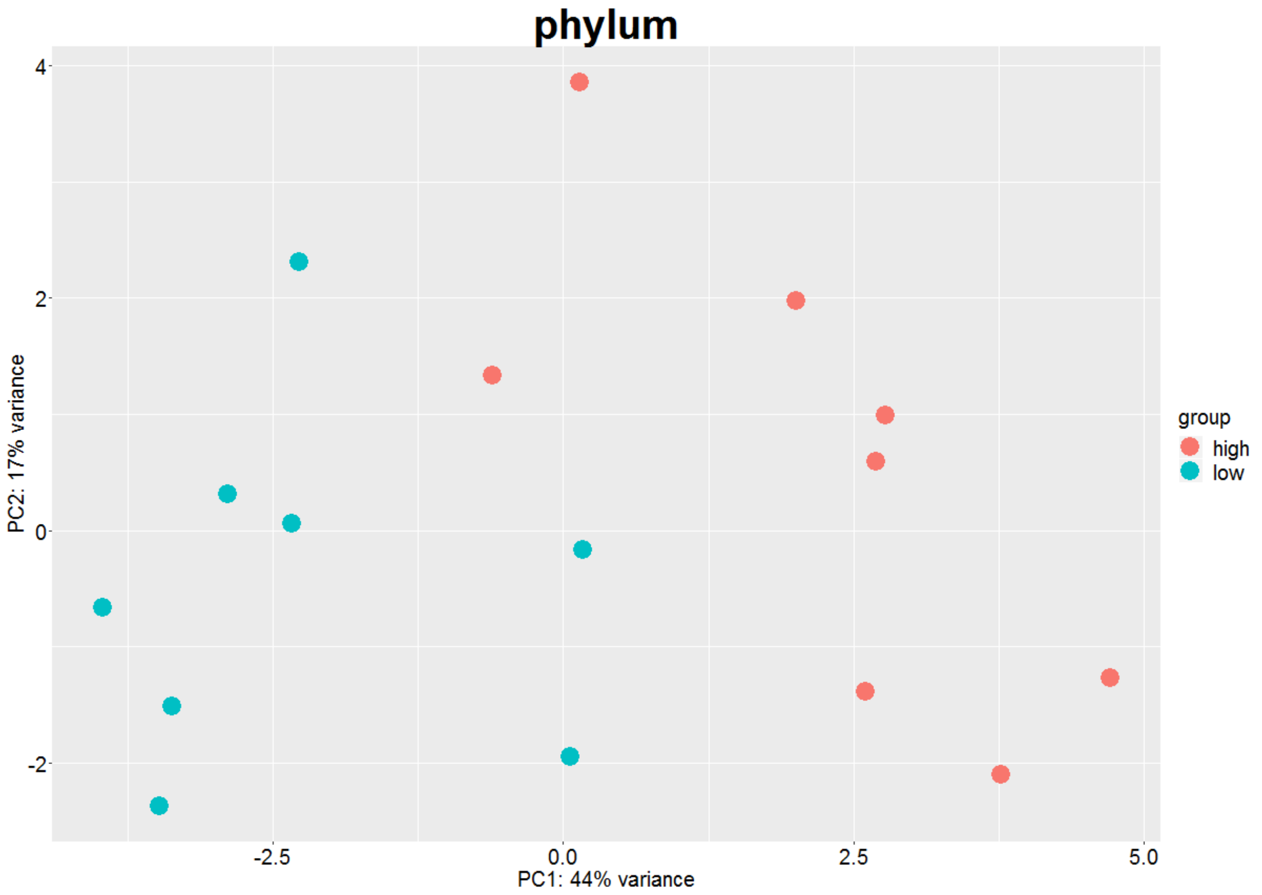

Supplementary Figure 5

Phylum-level PCA

Principal component analysis of phylum-level abundances comparing low (n=8 animals) and high (n=8 animals) methane emitting sheep

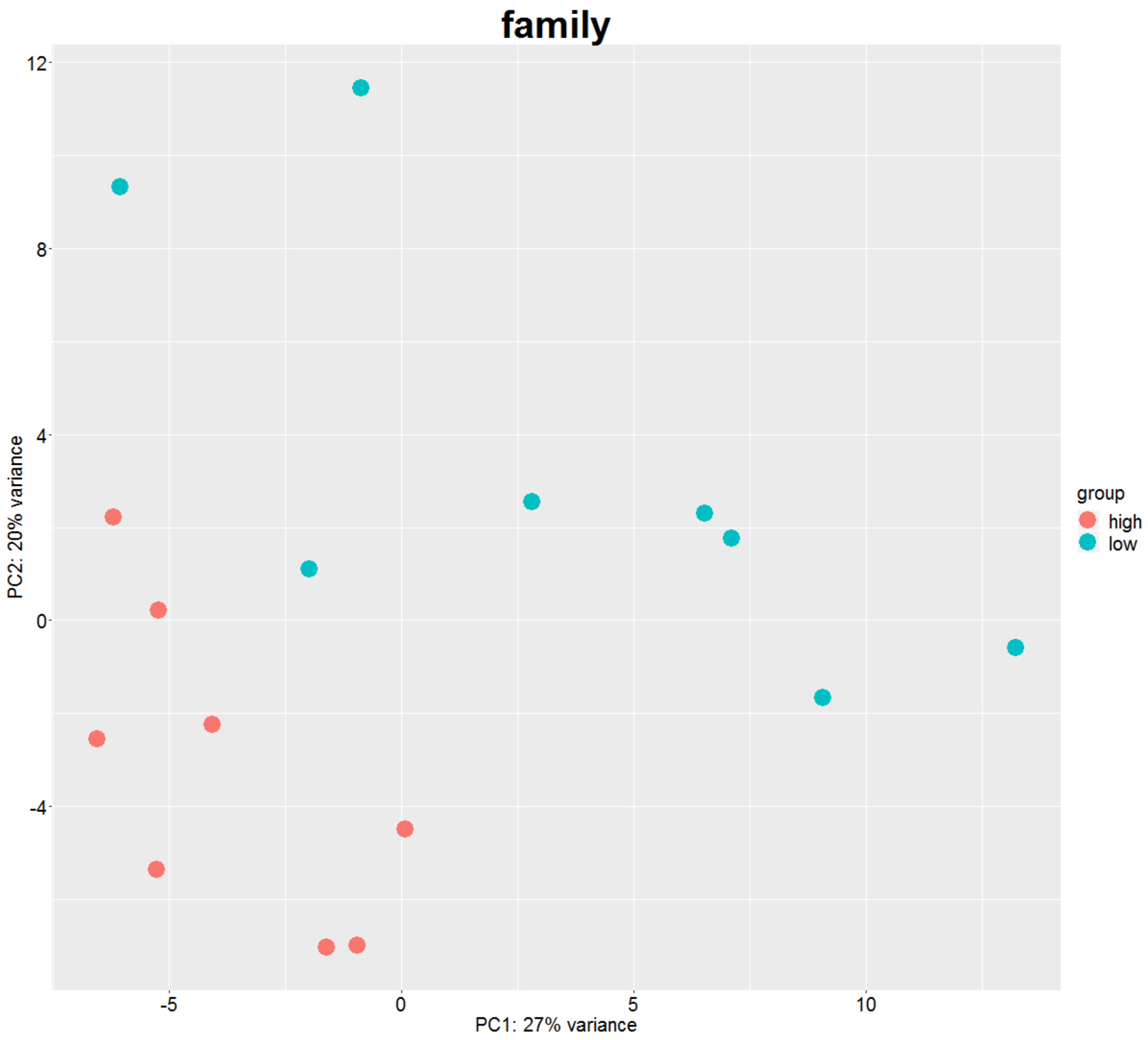

Supplementary Figure 6

Family-level PCA

Principal component analysis of family-level abundances comparing low (n=8 animals) and high (n=8 animals) methane emitting sheep

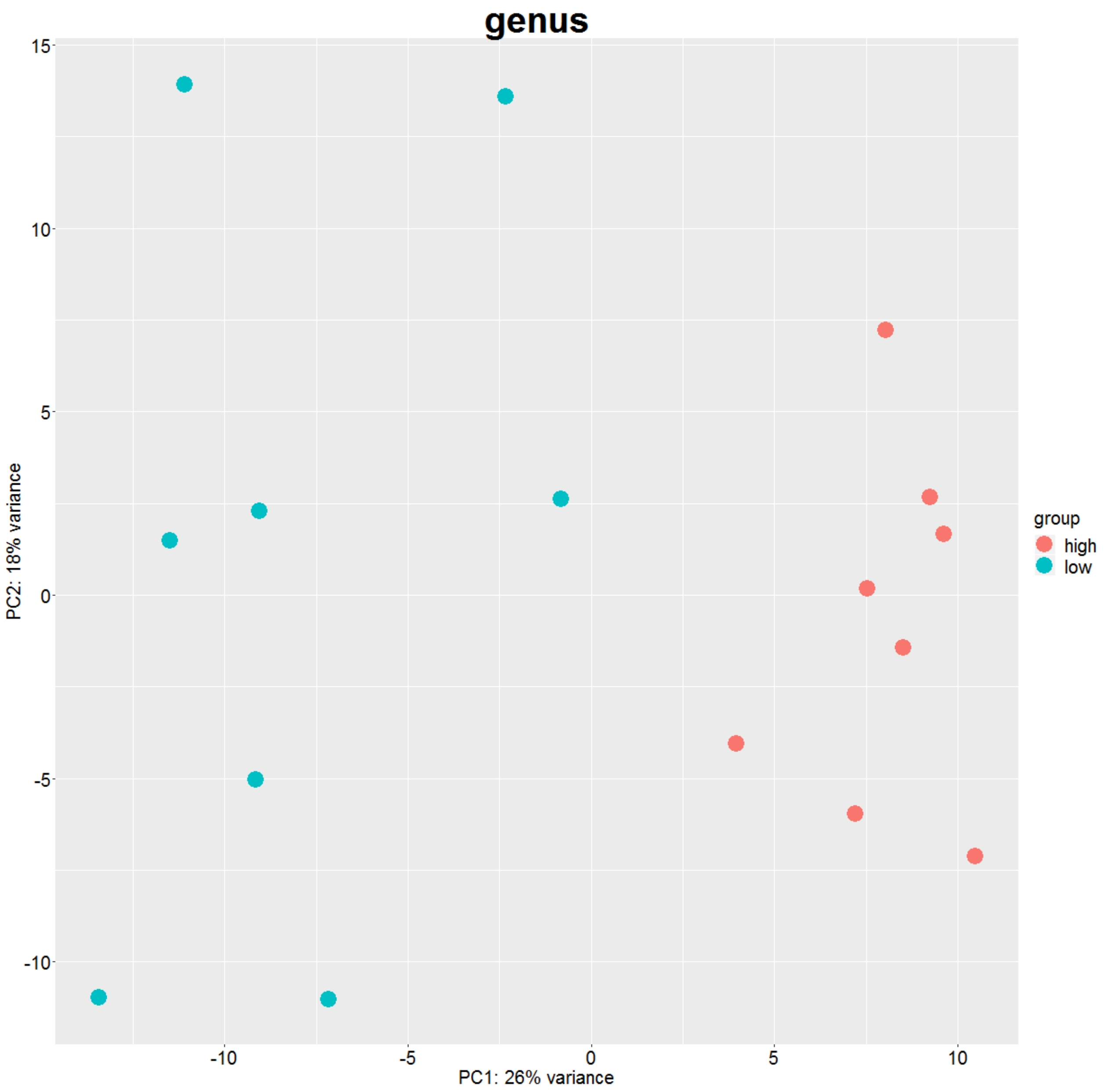

Supplementary Figure 7

Genus-level PCA

Principal component analysis of genus-level abundances comparing low (n=8 animals) and high (n=8 animals) methane emitting sheep

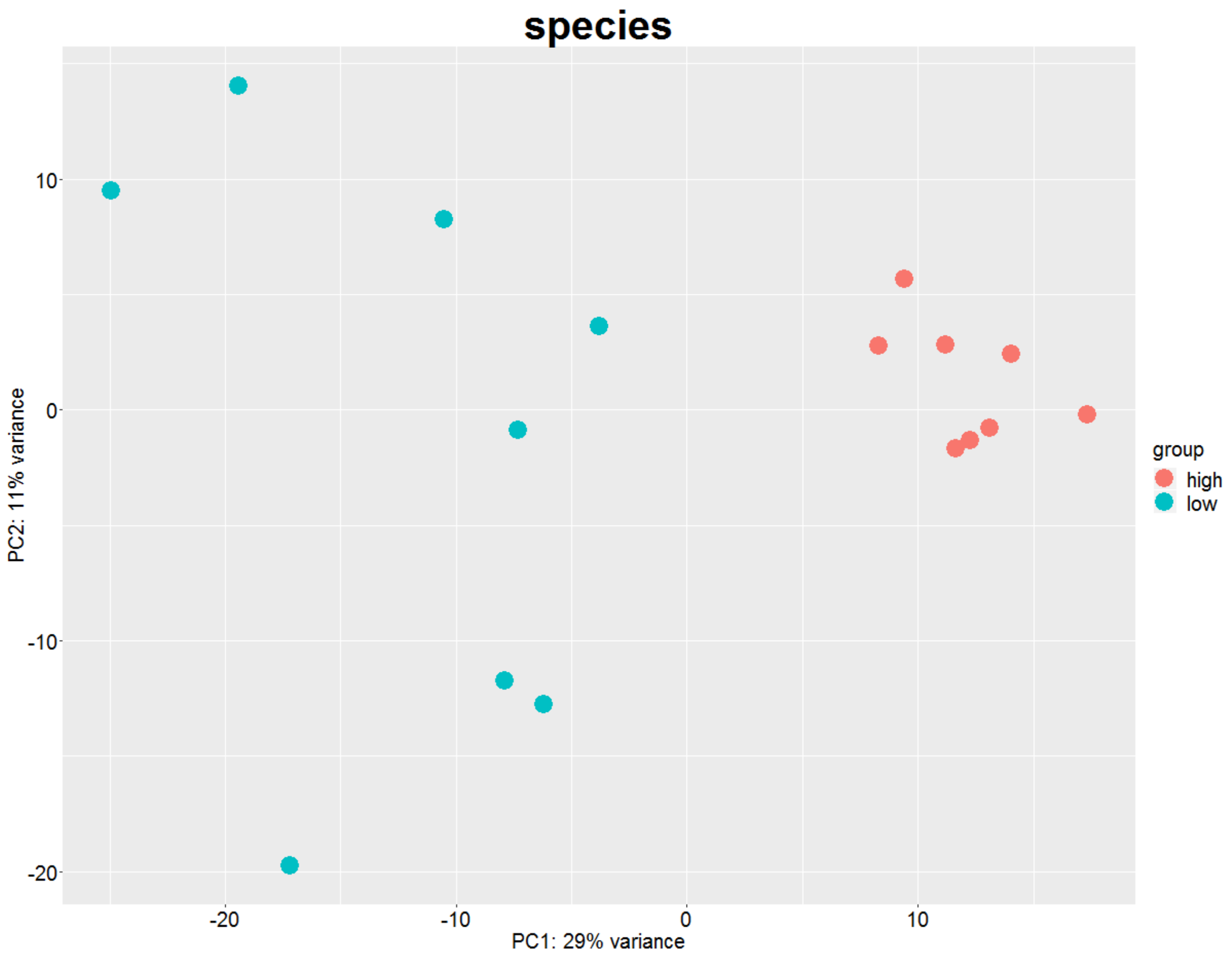

Supplementary Figure 8

Species-level PCA

Principal component analysis of species-level proportional read-count data from Kraken, comparing low (n=8 animals) and high (n=8 animals) methane emitting sheep

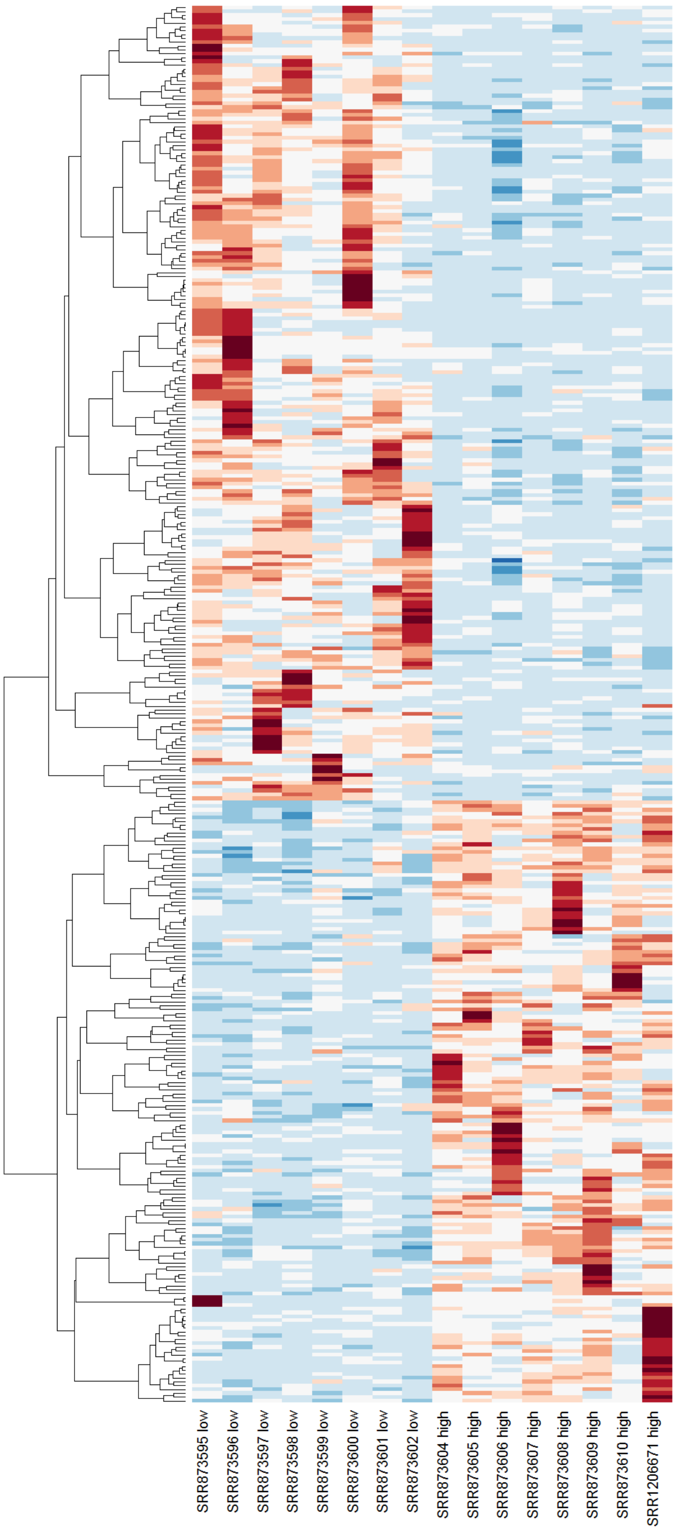
SRR873595 low
SRR873596 low
SRR873597 low
SRR873598 low
SRR873599 low
SRR873600 low
SRR873601 low
SRR873602 low
SRR873604 high
SRR873605 high
SRR873606 high
SRR873607 high
SRR873608 high
SRR873609 high
SRR873610 high
SRR1206671 high

Supplementary Figure 9

Species-level heatmap

Heatmap of species-level abundances comparing low (n=8 animals) and high (n=8 animals) methane emitting sheep. The colour scheme transitions from navy (low values) through white (medium values) to dark red (high values).

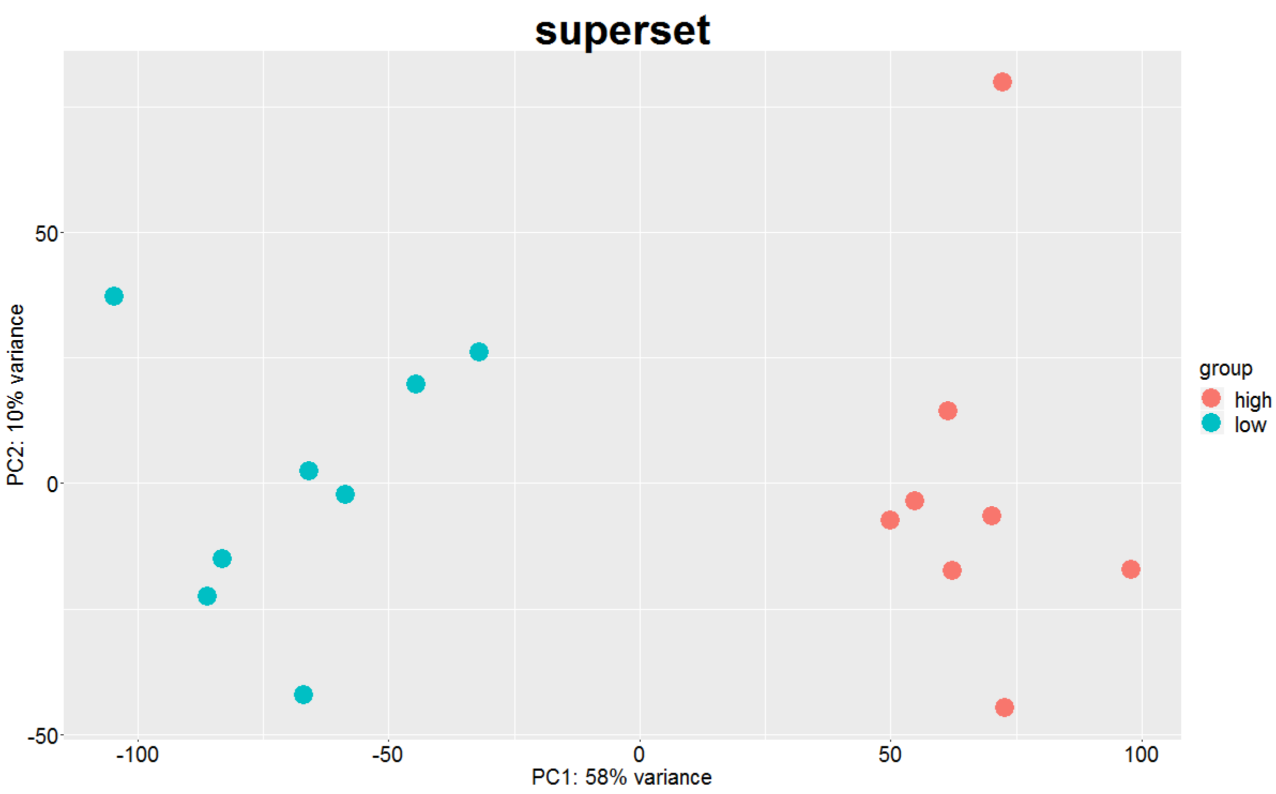

Supplementary Figure 10

Strain-level PCA

Principal component analysis plot of the abundance of rumen superset genomes from low (n=8 animals) and high (n=8 animals) methane emitting sheep

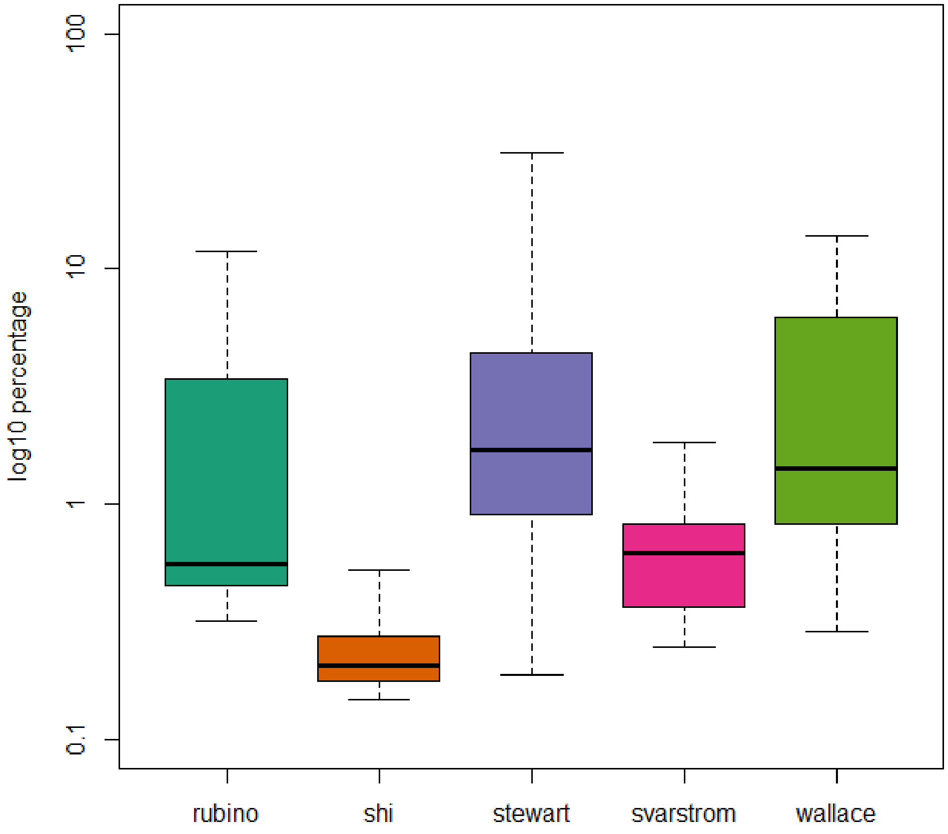

Supplementary Figure 11

Rumen *Proteobacteria* abundance

Boxplot of percentage abundance of *Proteobacteria* across 5 rumen metagenomic datasets. Y-axis is on a log10 scale. Sample sizes: Stewart (n=283 animals), Wallace (n=8 animals), Rubino (n=14 animals), Shi (n=16 animals), Svartström (n=6 animals). Centre line shows the median value; box shows the interquartile range; whiskers extend to the most extreme data point which is no more than 1.5 times the interquartile range from the box.

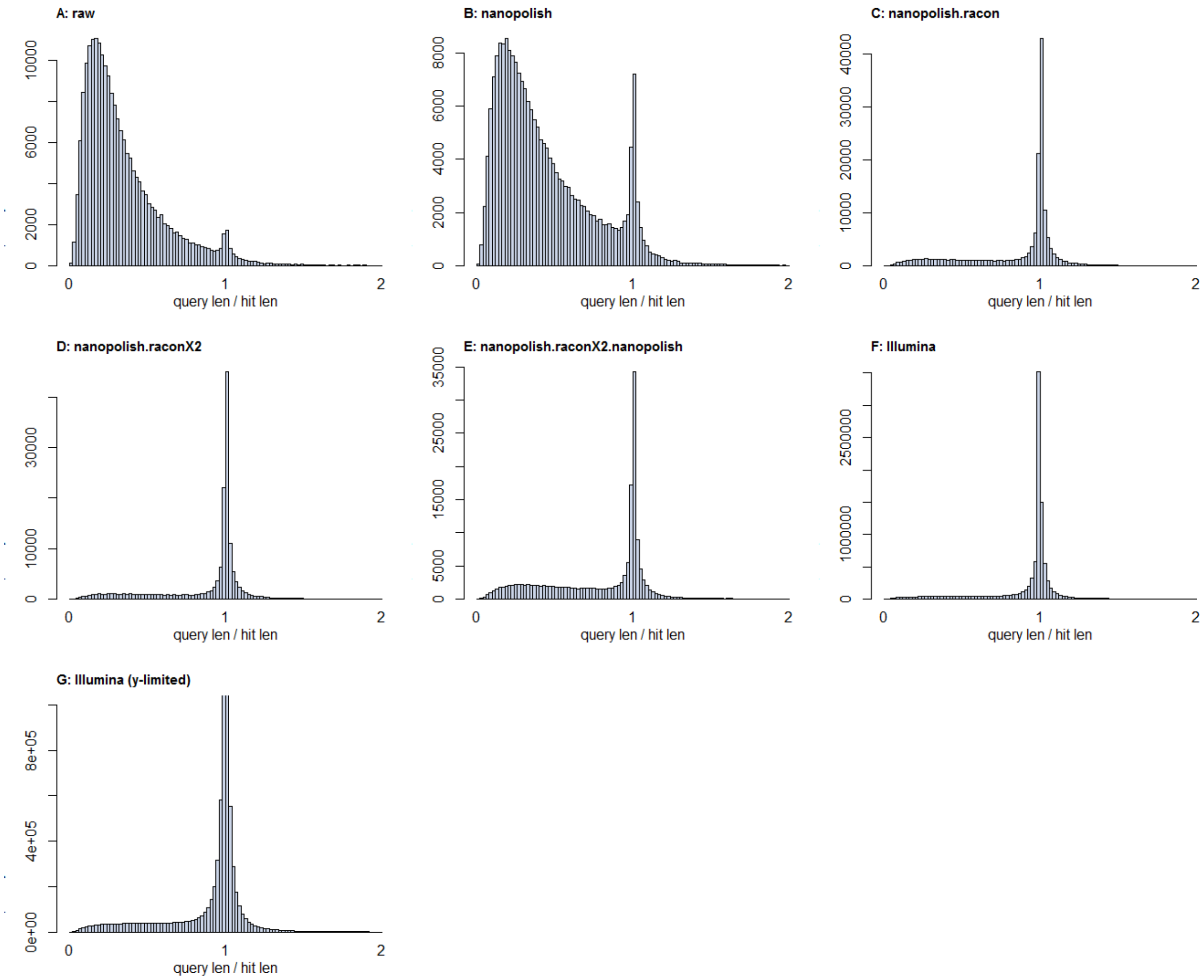

Supplementary Figure 12

Correcting errors in the MinION nanopore assembly

Histograms of predicted protein length vs length of the top hit in UniProt. Perfect predictions should show a tight distribution around 1. A) the raw assembly from Canu; B) after one round of Nanopolish; C) after one round of Nanopolish and one round of Racon; D) after one round of Nanopolish and two rounds of Racon; E) after one round of Nanopolish, two rounds of Racon, and a further round of Nanopolish; F) data from the 4941 RUGs; G) data from the 4941 RUGs with a limited y-axis, to highlight the long tail of short proteins remaining. the first round of Nanopolish produces a notable improvement, and the first round of Racon (with Illumina data) produces a drastic improvement. A second round of Racon (with Illumina data) produces a very slight improvement, and a final round of Nanopolish makes things slightly worse..

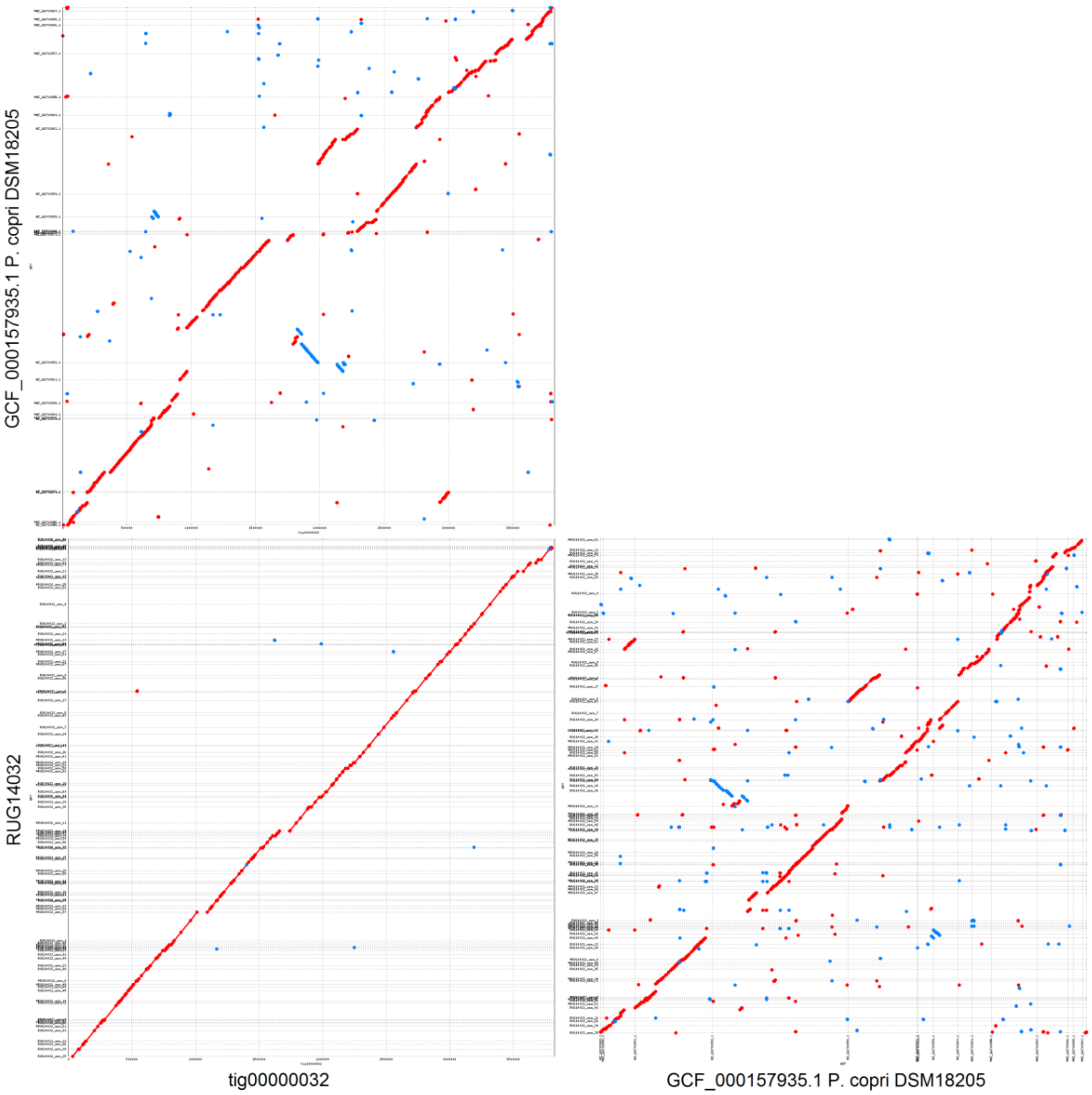

Supplementary Figure 13

Prevotella copri whole-genome alignments

Whole genome alignments between *Prevotella copri* nRUG14950 (tig00000032), RUG14032 and *Prevotella copri* DSM18205. RUG14032 and *Prevotella copri* DSM18205 exist as unordered contigs whereas *Prevotella copri* nRUG14950 (tig00000032) is a single contig assembled from Nanopore data.

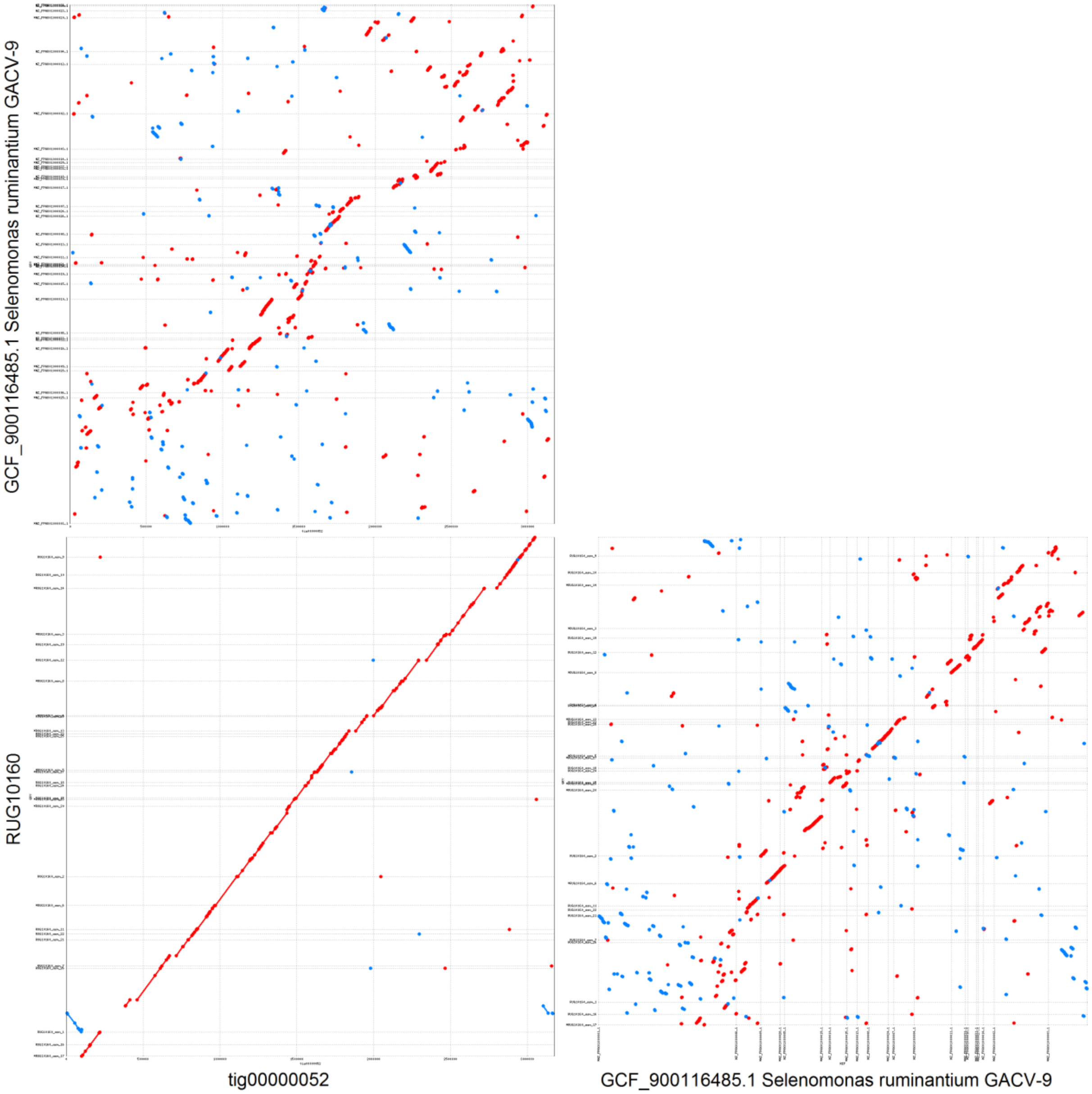

Supplementary Figure 14

Selenomonas ruminantium whole-genome alignments

Whole genome alignments between *Selenomonas* spp. nRUG14951 (tig00000052), RUG110160 and *Selenomonas ruminantium* GACV-9. RUG110160 and *Selenomonas ruminantium* GACV-9 exist as unordered contigs whereas *Selenomonas* spp nRUG14951 (tig00000052) is a single contig assembled from Nanopore data.

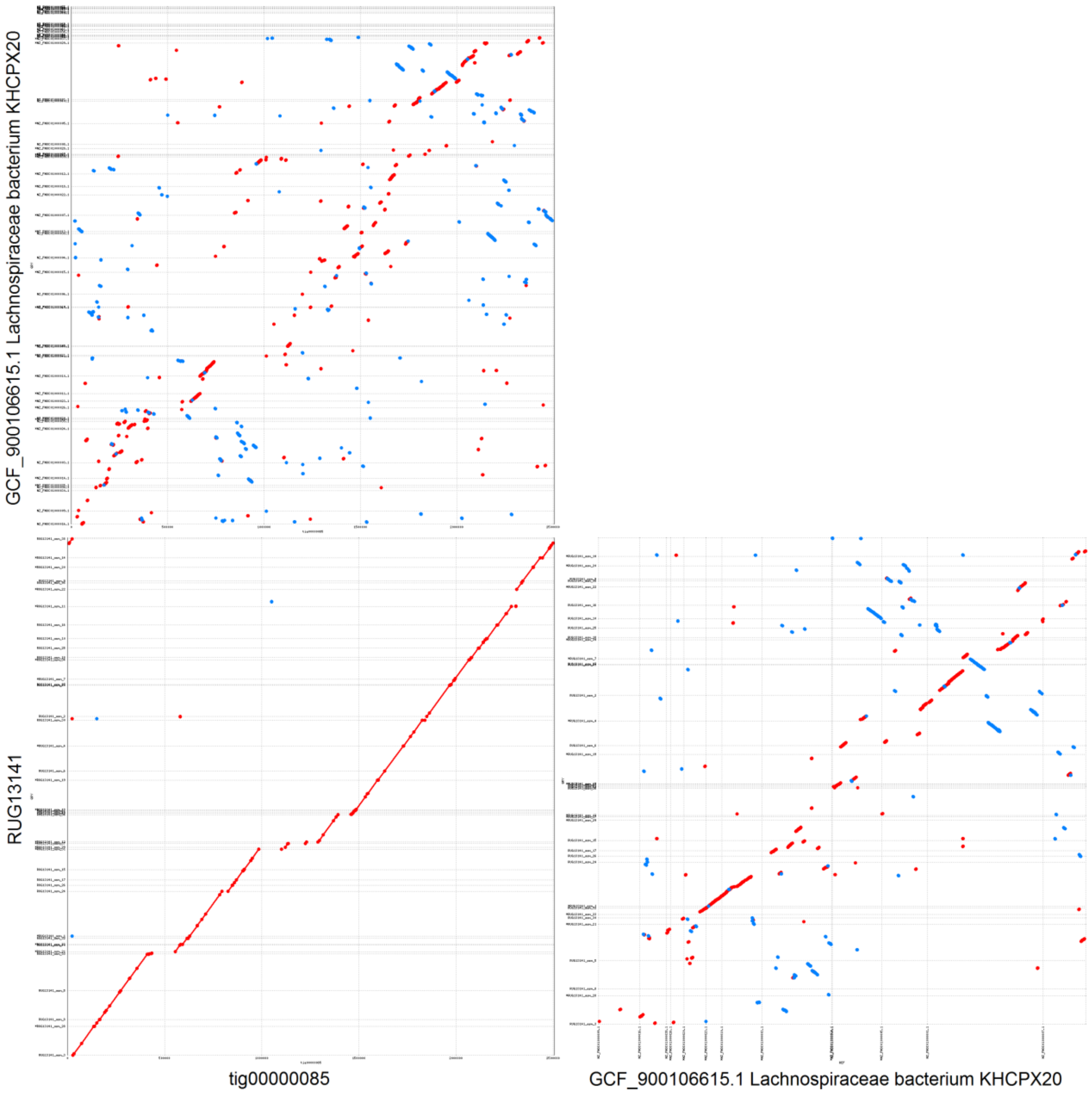

Supplementary Figure 15

Lachnospiraceae bacterium whole-genome alignments

Whole genome alignments between *Lachnospiraceae bacterium* nRUG14952 (tig00000085), RUG13141 and *Lachnospiraceae bacterium* KHCPX20. RUG13141 and *Lachnospiraceae bacterium* KHCPX20 exist as unordered contigs whereas *Lachnospiraceae bacterium* nRUG14952 (tig00000085) is a single contig assembled from Nanopore data.

Kingdom-level analysis of sheep methane data:

	Mean	log2FC	lfcSE	stat	pvalue	padj
Eukaryota	1431244.20	-0.03	0.20	-0.14	0.89	0.89
Bacteria	120475706.85	0.05	0.11	0.45	0.65	0.89
Archaea	2616380.24	-0.09	0.15	-0.56	0.58	0.89

Supplementary table 1 DESeq2 test results at the Kingdom level

DESeq2 test results at the Kingdom level between low (n=8 animals) and high (n=8 animals) emitting sheep. Mean is the average normalised counts across all samples; log2FC is the log2 ratio methane emissions low/high; lfcSE gives the standard error of the log2FC; stat is the Wald statistic: the log2FC divided by lfcSE, which is compared to a standard Normal distribution to generate a two-tailed pvalue; pvalue is the raw p-value, and padj is the adjusted p-value (Benjamini and Hochberg (1995) Journal of the Royal Statistical Society. Series B (Methodological), Vol. 57, No. 1 (1995), pp. 289300, also known as FDR)

Phylum-level analysis of sheep methane data:

	Mean	log2FC	lfcSE	stat	pvalue	padj
Elusimicrobia	189631.50	-3.82	0.86	-4.45	0.000	0.000
Planctomycetes	100098.54	1.12	0.26	4.35	0.000	0.000
Candidatus.Cloacimonetes	111.11	-0.91	0.24	-3.79	0.000	0.002
Caldiserica	134.03	-1.09	0.28	-3.85	0.000	0.002
Fibrobacteres	2968793.81	1.33	0.38	3.53	0.000	0.003
Actinobacteria	1870711.60	1.22	0.35	3.49	0.000	0.003
Aquificae	1352.04	0.53	0.15	3.58	0.000	0.003

Supplementary table 2 DESeq2 test results at the Phylum level

Significantly different phyla between low (n=8 animals) and high (n=8 animals) emitting sheep. Table and statistics calculated using DESeq2. Mean is the average normalised counts across all samples; log2FC is the log2 ratio methane emissions low/high; lfcSE gives the standard error of the log2FC; stat is the Wald statistic: the log2FC divided by lfcSE, which is compared to a standard Normal distribution to generate a two-tailed pvalue; pvalue is the raw p-value, and padj is the adjusted pvalue (Benjamini and Hochberg (1995) Journal of the Royal Statistical Society. Series B (Methodological), Vol. 57, No. 1 (1995), pp. 289-300, also known as FDR)

Family-level analysis of sheep methane data:

	Mean	log2FC	lfcSE	stat	pvalue	padj
Lactobacillaceae	3014821.58	4.09	0.38	10.87	0.000	0.000
Atopobiaceae	331021.69	3.39	0.56	6.02	0.000	0.000
Erysipelotrichaceae	5408568.71	1.39	0.26	5.41	0.000	0.000
Veillonellaceae	664223.19	3.39	0.73	4.64	0.000	0.000
Elusimicrobiaceae	186627.30	-3.82	0.85	-4.50	0.000	0.001
Endomicrobiaceae	858.00	-3.06	0.69	-4.44	0.000	0.001
Methanosaetaceae	182.59	-1.27	0.29	-4.41	0.000	0.001
Caldisphaeraceae	49.16	-1.31	0.31	-4.24	0.000	0.001
Bifidobacteriaceae	17318.10	1.44	0.35	4.15	0.000	0.002
Segniliparaceae	100.87	-0.84	0.21	-4.03	0.000	0.003
Gloeomargaritaceae	106.65	-1.20	0.30	-3.97	0.000	0.003

Fibrobacteraceae	3020004.05	1.41	0.38	3.72	0.000	0.007
Cyphellophoraceae	71.71	-0.76	0.20	-3.72	0.000	0.007
Roseiflexaceae	426.52	-0.59	0.16	-3.65	0.000	0.009
Dermocarpellaceae	561.91	0.64	0.18	3.61	0.000	0.009
Caldisericaceae	132.95	-0.99	0.28	-3.51	0.000	0.013
Thermoproteaceae	78.93	0.79	0.23	3.37	0.001	0.020
Hydrogenothermaceae	751.23	0.79	0.24	3.24	0.001	0.028
Candidatus.Paracaedibacteraceae	59.14	-1.11	0.34	-3.24	0.001	0.028
Selenomonadaceae	1864493.41	1.38	0.43	3.22	0.001	0.029
Thiobacillaceae	170.72	-0.54	0.17	-3.19	0.001	0.031
Streptococcaceae	29812.26	1.96	0.62	3.18	0.001	0.031
Kosmotogaceae	282.97	0.83	0.26	3.13	0.002	0.033
Hyphomonadaceae	336.20	-0.53	0.17	-3.14	0.002	0.033
Syntrophaceae	622.17	-0.63	0.20	-3.10	0.002	0.036
Phycisphaeraceae	227.06	-0.55	0.18	-3.06	0.002	0.039
Babesiidae	77979.48	1.31	0.43	3.02	0.003	0.042
Unikaryonidae	158.82	1.24	0.41	3.01	0.003	0.042

Supplementary table 3 DESeq2 test results at the Family level

Significantly different families between low (n=8 animals) and high (n=8 animals) emitting sheep. Table and statistics calculated using DESeq2. Mean is the average normalised counts across all samples; log2FC is the log2 ratio methane emissions low/high; lfcSE gives the standard error of the log2FC; stat is the Wald statistic: the log2FC divided by lfcSE, which is compared to a standard Normal distribution to generate a two-tailed pvalue; pvalue is the raw p-value, and padj is the adjusted p-value (Benjamini and Hochberg (1995) Journal of the Royal Statistical Society. Series B (Methodological), Vol. 57, No. 1 (1995), pp. 289-300, also known as FDR)

Genus-level analysis of sheep methane data:

	**Mean**	**log2FC**	**lfcSE**	**stat**	**pvalue**	**padj**
Sharpea	3039009.65	4.18	0.39	10.77	0.000	0.000
Coriobacterium	5514.43	3.29	0.36	9.21	0.000	0.000
Olsenella	326610.95	3.52	0.46	7.66	0.000	0.000
Libanicoccus	11000.35	2.24	0.33	6.86	0.000	0.000
Megasphaera	610076.74	4.77	0.73	6.53	0.000	0.000
Clostridium	100019.64	1.38	0.22	6.28	0.000	0.000
Pyrobaculum	17.88	3.20	0.54	5.94	0.000	0.000
Atopobium	1046.62	1.44	0.26	5.53	0.000	0.000
Sphingorhabdus	118.49	1.14	0.21	5.40	0.000	0.000
Kozakia	91.31	-1.47	0.28	-5.32	0.000	0.000
Ogataea	145.27	-1.38	0.26	-5.29	0.000	0.000
Kandleria	1548098.85	4.12	0.81	5.10	0.000	0.000
Parascardovia	440.57	1.10	0.22	5.08	0.000	0.000
Thermocrinis	176.72	1.45	0.29	5.05	0.000	0.000
Erysipelothrix	194715.13	2.00	0.40	5.00	0.000	0.000
Hydrogenovibrio	85.23	-1.17	0.24	-4.85	0.000	0.000
Halobacillus	315.91	1.82	0.38	4.77	0.000	0.000
Desulfobacca	122.86	-1.27	0.27	-4.72	0.000	0.000
Desulfomonile	202.93	-1.00	0.21	-4.67	0.000	0.000
Elusimicrobium	186584.23	-3.81	0.84	-4.51	0.000	0.000

Endomicrobium	857.56	-3.05	0.69	-4.45	0.000	0.000
Thioflavicoccus	258.49	-1.02	0.23	-4.44	0.000	0.001
Moraxella	554.08	1.03	0.23	4.42	0.000	0.001
Labrenzia	202.82	0.89	0.20	4.40	0.000	0.001
Xylella	74.47	-0.95	0.22	-4.38	0.000	0.001
Halostagnicola	56.85	-1.29	0.30	-4.31	0.000	0.001
Methanothrix	182.34	-1.23	0.29	-4.30	0.000	0.001
Bifidobacterium	15950.67	1.54	0.36	4.28	0.000	0.001
Cryptobacterium	388.20	0.82	0.20	4.20	0.000	0.001
Mitsuokella	10718.38	2.74	0.66	4.16	0.000	0.001
Caldisphaera	49.23	-1.27	0.31	-4.12	0.000	0.001
Thermosynechococcus	279.73	0.78	0.19	4.00	0.000	0.002
Kingella	433.59	1.92	0.49	3.92	0.000	0.003
Faecalibaculum	1209.32	1.22	0.31	3.91	0.000	0.003
Aciduliprofundum	33.49	-1.50	0.38	-3.89	0.000	0.003
Gloeomargarita	106.79	-1.17	0.30	-3.89	0.000	0.003
Arthrospira	708.32	0.65	0.17	3.89	0.000	0.003
Segniliparus	101.27	-0.81	0.21	-3.87	0.000	0.003
Aureococcus	848.19	-0.48	0.12	-3.87	0.000	0.003
Candidatus.Methanoplasma	830.16	-1.56	0.40	-3.88	0.000	0.003
Thermogymnomonas	21.57	-1.63	0.43	-3.84	0.000	0.004
Mannheimia	2109.20	1.51	0.39	3.84	0.000	0.004
Pseudodesulfovibrio	1265.16	-0.51	0.13	-3.83	0.000	0.004
Gemella	747.76	1.25	0.33	3.82	0.000	0.004
Aminobacterium	188.49	1.63	0.43	3.76	0.000	0.005
Cyphellophora	71.86	-0.73	0.20	-3.74	0.000	0.005
Fibrobacter	3025238.97	1.41	0.38	3.73	0.000	0.005
Stanieria	567.79	0.67	0.18	3.72	0.000	0.005
Trueperella	282.48	0.76	0.21	3.70	0.000	0.005
Candidatus.Symbiobacter	364.55	1.50	0.41	3.67	0.000	0.006
Roseiflexus	427.91	-0.57	0.16	-3.63	0.000	0.007
Turneriella	139.30	-1.08	0.30	-3.63	0.000	0.007
Candidatus.Xiphinematobacter	37.07	-1.18	0.33	-3.58	0.000	0.007
Methanomassiliicoccus	384.67	-1.05	0.29	-3.59	0.000	0.007
Dorea	63849.88	2.64	0.74	3.56	0.000	0.008
Zunongwangia	116.55	-0.88	0.25	-3.50	0.000	0.010
Acidaminococcus	18160.65	1.03	0.29	3.49	0.000	0.010
Ruegeria	204.77	-0.56	0.16	-3.44	0.001	0.011
Croceicoccus	152.84	0.75	0.22	3.45	0.001	0.011
Cedecea	336.71	-0.57	0.17	-3.45	0.001	0.011
Chitinophaga	577.21	-0.83	0.24	-3.46	0.001	0.011
Acetobacter	400.22	0.64	0.19	3.43	0.001	0.011
Caldisericum	133.26	-0.97	0.28	-3.41	0.001	0.012
Paludisphaera	246.02	-0.65	0.19	-3.41	0.001	0.012
Nitrosospira	174.52	-1.03	0.30	-3.41	0.001	0.012
Jeotgalicoccus	339.63	1.29	0.38	3.40	0.001	0.012
Methylotenera	125.08	-1.09	0.32	-3.39	0.001	0.012
Candidatus.Cloacimonas	111.10	-0.81	0.24	-3.39	0.001	0.012
Sphingopyxis	999.84	-0.51	0.15	-3.37	0.001	0.013
Methanotorris	283.74	1.05	0.31	3.36	0.001	0.013
Scardovia	126.35	0.92	0.28	3.31	0.001	0.016

Blautia	30222.90	0.76	0.23	3.29	0.001	0.016
Adlercreutzia	3823.54	0.77	0.24	3.27	0.001	0.017
Verminephrobacter	247.31	0.52	0.16	3.27	0.001	0.017
Pusillimonas	115.16	-0.68	0.21	-3.27	0.001	0.017
Lachnobacterium	13790.00	0.62	0.19	3.28	0.001	0.017
Halosimplex	50.39	-0.77	0.23	-3.28	0.001	0.017
Hafnia	123.88	-0.72	0.22	-3.27	0.001	0.017
Halioglobus	120.85	0.67	0.21	3.26	0.001	0.017
Echinicola	219.87	0.89	0.27	3.26	0.001	0.017
Hydrogenobacter	30.47	-1.08	0.34	-3.21	0.001	0.019
Candidatus.Paracaedibacter	59.34	-1.09	0.34	-3.19	0.001	0.020
Defluviitoga	180.30	0.74	0.24	3.11	0.002	0.027
Haladaptatus	154.83	-0.55	0.18	-3.10	0.002	0.027
Phycisphaera	227.29	-0.53	0.17	-3.11	0.002	0.027
Arcanobacterium	52.06	-0.80	0.26	-3.09	0.002	0.028
Kosmotoga	136.31	1.04	0.34	3.07	0.002	0.029
Methanomethylovorans	28.36	-0.79	0.26	-3.07	0.002	0.029
Haloarcula	196.95	-0.67	0.22	-3.06	0.002	0.029
Sulfurihydrogenibium	640.52	0.84	0.28	3.06	0.002	0.029
Haloferax	412.26	-0.69	0.22	-3.06	0.002	0.029
Actinotignum	131.08	0.71	0.23	3.04	0.002	0.031
Sorangium	718.69	-0.42	0.14	-3.03	0.002	0.031
Desulfovibrio	45823.70	1.06	0.35	3.04	0.002	0.031
Thermococcus	345.98	-0.49	0.16	-3.03	0.002	0.031
Babesia	78930.33	1.34	0.44	3.02	0.003	0.032
Hyphomonas	190.01	-0.51	0.17	-3.01	0.003	0.032
Thiobacillus	171.65	-0.51	0.17	-3.00	0.003	0.032
Alcaligenes	117.09	-0.60	0.20	-3.00	0.003	0.032
Halobellus	25.20	-0.87	0.29	-3.00	0.003	0.032
Encephalitozoon	162.61	1.28	0.43	3.00	0.003	0.032
Actinomyces	3969.70	0.58	0.19	2.98	0.003	0.034
Faecalitalea	2649.19	0.83	0.28	2.98	0.003	0.034
Methylocella	123.08	-0.56	0.19	-2.96	0.003	0.035
Haloprofundus	28.75	0.87	0.30	2.94	0.003	0.038
Methylocystis	226.03	-0.83	0.28	-2.92	0.003	0.039
Ignicoccus	4.78	-1.78	0.62	-2.89	0.004	0.043
Clostridioides	4003.61	0.64	0.22	2.89	0.004	0.043
Singulisphaera	218.59	-0.63	0.22	-2.88	0.004	0.043
Shinella	273.67	-0.56	0.19	-2.87	0.004	0.045
Halomicrobium	41.91	-0.84	0.29	-2.86	0.004	0.045
Saprolegnia	800.63	0.55	0.19	2.86	0.004	0.046

Supplementary table 4 DESeq2 test results at the Genus level

Significantly different genera between low (n=8 animals) and high (n=8 animals) emitting sheep. Table and statistics calculated using DESeq2. Mean is the average normalised counts across all samples; log2FC is the log2 ratio methane emissions low/high; lfcSE gives the standard error of the log2FC; stat is the Wald statistic: the log2FC divided by lfcSE, which is compared to a standard Normal distribution to generate a two-tailed pvalue; pvalue is the raw p-value, and padj is the adjusted p-value (Benjamini and Hochberg (1995) Journal of the Royal Statistical Society. Series B (Methodological), Vol. 57, No. 1 (1995), pp. 289-300, also known as FDR)

Species-level analysis of sheep methane data:

	Mean	log2FC	lfcSE	stat	pvalue	padj
Clostridium.sp..SY8519	43980.86	3.59	0.32	11.16	0.000	0.000
Sharpea.azabuensis	3046715.22	4.19	0.40	10.50	0.000	0.000
Olsenella.sp..KH3B4	18593.99	4.58	0.44	10.47	0.000	0.000
Coriobacterium.glomerans	5520.42	3.29	0.37	8.83	0.000	0.000
Eubacterium.pyruvativorans	38356.88	3.52	0.45	7.78	0.000	0.000
Geobacillus.sp..WCH70	531.04	2.41	0.32	7.46	0.000	0.000
Desulfovibrio.magneticus	539.02	-0.95	0.13	-7.45	0.000	0.000
Lactobacillus.ruminis	1066.96	1.62	0.22	7.36	0.000	0.000
Desulfovibrio.vulgaris	1818.29	-1.13	0.16	-6.87	0.000	0.000
Actinomyces.sp..oral.taxon.414	1084.53	2.13	0.31	6.87	0.000	0.000
Butyrivibrio.sp..XBB1001	20637.79	-2.24	0.33	-6.79	0.000	0.000
Libanicoccus.massiliensis	11035.14	2.25	0.34	6.64	0.000	0.000
Olsenella.sp..kh2p3	1333.23	2.37	0.37	6.42	0.000	0.000
Bifidobacterium.coryneforme	54.30	2.29	0.36	6.37	0.000	0.000
Megasphaera.elsdenii	158831.98	6.20	0.98	6.31	0.000	0.000
Natrinema.gari	20.68	2.85	0.46	6.25	0.000	0.000
Butyrivibrio.sp..INlla14	25919.64	-1.93	0.31	-6.23	0.000	0.000
Streptococcus.macedonicus	189.53	3.76	0.61	6.18	0.000	0.000
Bifidobacterium.merycicum	2084.62	1.57	0.26	6.14	0.000	0.000
Bifidobacterium.catenulatum	197.40	2.06	0.34	6.08	0.000	0.000
Bifidobacterium.breve	941.55	2.42	0.40	6.00	0.000	0.000
Streptococcus.gallolyticus	4210.18	5.29	0.88	5.98	0.000	0.000
Ogataea.parapolymorpha	98.41	-1.84	0.32	-5.80	0.000	0.000
Bifidobacterium.thermophilum	366.41	2.27	0.39	5.77	0.000	0.000
Thermocrinis.ruber	129.57	2.09	0.36	5.77	0.000	0.000
Pyrobaculum.islandicum	17.85	3.19	0.56	5.72	0.000	0.000
Pseudomonas.parafulva	140.65	-1.18	0.21	-5.73	0.000	0.000
Desulfovibrio.piger	2419.86	-1.19	0.21	-5.59	0.000	0.000
Lachnospiraceae.bacterium.MC2017	18389.45	2.91	0.52	5.55	0.000	0.000
Desulfovibrio.gigas	586.93	-1.19	0.22	-5.50	0.000	0.000
Atopobium.parvulum	1045.03	1.44	0.27	5.43	0.000	0.000
Desulfovibrio.sp..FW1012B	579.98	-0.95	0.18	-5.39	0.000	0.000
Mitsuokella.jalaludinii	1462.51	2.74	0.51	5.35	0.000	0.000
Mycobacterium.haemophilum	108.09	2.03	0.38	5.34	0.000	0.000
Sphingorhabdus.sp..M41	118.42	1.14	0.21	5.31	0.000	0.000
Kozakia.baliensis	91.56	-1.48	0.28	-5.22	0.000	0.000
Bifidobacterium.boum	704.90	1.72	0.33	5.20	0.000	0.000
Kandleria.vitulina	1550272.93	4.15	0.81	5.09	0.000	0.000
Roseiflexus.sp..RS.1	207.86	-0.84	0.17	-5.06	0.000	0.000
Ruegeria.pomeroyi	132.30	-0.80	0.16	-5.03	0.000	0.000
Parascardovia.denticolens	440.36	1.10	0.22	5.00	0.000	0.000
Endomicrobium.proavitum	848.85	-3.04	0.61	-5.00	0.000	0.000
Bacillus.anthracis	363.27	0.96	0.20	4.90	0.000	0.000
Acidaminococcus.fermentans	2612.37	2.22	0.46	4.88	0.000	0.000
Erysipelothrix.rhusiopathiae	194528.49	2.00	0.42	4.83	0.000	0.000
Acetobacter.pasteurianus	175.82	1.28	0.27	4.79	0.000	0.000

Acinetobacter.venetianus	194.70	1.68	0.36	4.71	0.000	0.000
Azospirillum.humicireducens	64.32	-0.88	0.19	-4.71	0.000	0.000
Thermoplasma.acidophilum	7.10	-2.54	0.54	-4.72	0.000	0.000
Lachnospiraceae.bacterium.NK3A20	9949.69	1.34	0.29	4.70	0.000	0.000
Bifidobacterium.pseudolongum	377.82	2.14	0.46	4.69	0.000	0.000
Hydrogenovibrio.crunogenus	85.73	-1.18	0.25	-4.67	0.000	0.000
Halobacillus.halophilus	315.16	1.82	0.39	4.65	0.000	0.000
Butyrivibrio.hungatei	26630.43	-1.50	0.32	-4.66	0.000	0.000
Streptococcus.constellatus	148.30	0.97	0.21	4.65	0.000	0.000
Desulfobacca.acetoxidans	123.22	-1.29	0.28	-4.61	0.000	0.000
Desulfomonile.tiedjei	203.24	-1.01	0.22	-4.59	0.000	0.000
X.Eubacterium..eligens	4979.02	1.83	0.40	4.57	0.000	0.000
Myxococcus.xanthus	141.73	-0.79	0.17	-4.56	0.000	0.000
Erysipelotrichaceae.bacterium.I46	315.21	1.21	0.27	4.52	0.000	0.000
Bacillus.pseudomycoides	84.61	2.18	0.48	4.51	0.000	0.000
Halorubrum.sp..AJ67	31.39	-2.67	0.59	-4.51	0.000	0.000
Lachnospiraceae.bacterium.NK4A144	28194.58	1.75	0.39	4.49	0.000	0.000
Enterococcus.faecium	627.98	1.10	0.25	4.45	0.000	0.000
Streptomyces.pristinaespiralis	372.93	0.62	0.14	4.45	0.000	0.000
Oribacterium.sp..WCC10	23121.83	1.71	0.39	4.42	0.000	0.000
Olsenella.umbonata	115915.95	4.35	0.98	4.41	0.000	0.000
Thioflavicoccus.mobilis	258.74	-1.03	0.23	-4.41	0.000	0.000
Bifidobacterium.ruminantium	68.65	2.10	0.48	4.39	0.000	0.000
Labrenzia.sp..CP4	202.60	0.88	0.20	4.39	0.000	0.000
Caldicellulosiruptor.hydrothermalis	203.89	1.34	0.31	4.37	0.000	0.001
Methanothrix.soehngenii	90.55	-1.76	0.40	-4.36	0.000	0.001
Acidaminococcus.intestini	4230.63	1.55	0.36	4.34	0.000	0.001
Lachnospiraceae.bacterium.G41	2398.18	-1.16	0.27	-4.34	0.000	0.001
Dorea.sp..AGR2135	4770.45	1.55	0.36	4.34	0.000	0.001
Xylella.fastidiosa	74.61	-0.95	0.22	-4.29	0.000	0.001
Olsenella.sp..Marseille.P2300	11132.78	1.50	0.35	4.28	0.000	0.001
Pseudomonas.syringae.group.genomosp..3	121.67	-1.45	0.34	-4.28	0.000	0.001
Butyrivibrio.sp..AE3003	1842.04	-1.42	0.33	-4.24	0.000	0.001
Cupriavidus.necator	271.01	0.79	0.19	4.24	0.000	0.001
Exiguobacterium.sp..MH3	72.21	1.44	0.34	4.23	0.000	0.001
Cryptobacterium.curtum	387.73	0.82	0.20	4.20	0.000	0.001
Yersinia.rohdei	89.05	-2.39	0.57	-4.18	0.000	0.001
Streptococcus.dysgalactiae	247.13	1.17	0.28	4.18	0.000	0.001
Marinobacter.adhaerens	37.39	-1.17	0.28	-4.18	0.000	0.001
Halobacterium.sp..DL1	56.79	1.16	0.28	4.16	0.000	0.001
Aureococcus.anophagefferens	847.87	-0.49	0.12	-4.16	0.000	0.001
Geoglobus.ahangari	26.09	-1.65	0.40	-4.16	0.000	0.001
Planctomyces.sp..SH.PL62	291.27	-0.46	0.11	-4.14	0.000	0.001
Prevotella.bryantii	385947.24	3.81	0.92	4.13	0.000	0.001
Methanotorris.formicicus	137.44	1.58	0.38	4.12	0.000	0.001
Fusobacterium.necrophorum	1427.72	1.37	0.33	4.12	0.000	0.001
Staphylococcus.schleiferi	370.87	1.23	0.30	4.06	0.000	0.002
Pectobacterium.wasabiae	71.11	0.98	0.24	4.05	0.000	0.002
Mycobacterium.sp..YC.RL4	136.05	0.91	0.22	4.05	0.000	0.002
Massilia.putida	387.68	-0.87	0.22	-4.03	0.000	0.002
Desulfovibrio.fairfieldensis	1569.00	-0.92	0.23	-4.01	0.000	0.002

Mucilaginibacter.paludis	295.22	-0.97	0.24	-4.01	0.000	0.002
Methanosarcina.mazei	399.37	0.91	0.23	4.01	0.000	0.002
Actinobacillus.equuli	124.60	1.36	0.34	4.00	0.000	0.002
Corynebacterium.uterequi	158.72	0.91	0.23	3.96	0.000	0.002
Corynebacterium.glyciniphilum	363.86	2.48	0.63	3.95	0.000	0.002
Mannheimia.haemolytica	1610.28	1.71	0.43	3.95	0.000	0.002
Caldisphaera.lagunensis	49.45	-1.27	0.32	-3.94	0.000	0.002
Lactobacillus.paraplantarum	33.85	-1.40	0.36	-3.94	0.000	0.002
Bordetella.parapertussis	15.87	1.41	0.36	3.94	0.000	0.002
Streptococcus.anginosus	364.92	0.73	0.19	3.92	0.000	0.003
Methylomicrobium.album	214.14	-0.69	0.18	-3.93	0.000	0.003
Pseudodesulfovibrio.indicus	513.81	-0.72	0.18	-3.92	0.000	0.003
Methanosarcina.sp..MTP4	138.14	-1.00	0.25	-3.91	0.000	0.003
Sphaerochaeta.coccoides	405.13	0.94	0.24	3.90	0.000	0.003
Microbacterium.testaceum	338.01	0.65	0.17	3.90	0.000	0.003
Blautia.wexlerae	4424.26	1.03	0.27	3.89	0.000	0.003
Moraxella.ovis	247.67	1.42	0.37	3.89	0.000	0.003
Novosphingobium.sp..PP1Y	56.72	-0.97	0.25	-3.88	0.000	0.003
Halorhabdus.tiamatea	28.47	-1.91	0.49	-3.86	0.000	0.003
Brucella.sp..09RB8471	16.70	-1.24	0.32	-3.84	0.000	0.003
Bifidobacterium.longum	842.90	0.78	0.20	3.84	0.000	0.003
Sulfurospirillum.sp..UCH001	103.20	-1.82	0.47	-3.83	0.000	0.003
Candidatus.Methanomassiliicoccus.intestinalis	43.50	-1.15	0.30	-3.83	0.000	0.003
Gloeomargarita.lithophora	106.66	-1.17	0.31	-3.82	0.000	0.003
Candidatus.Methanoplasma.termitum	827.77	-1.57	0.41	-3.82	0.000	0.003
Segniliparus.rotundus	101.40	-0.82	0.21	-3.82	0.000	0.003
Methylotenera.versatilis	76.72	-1.76	0.46	-3.82	0.000	0.003
Alteromonas.sp..RW2A1	57.97	1.36	0.36	3.82	0.000	0.003
Streptococcus.mutans	275.21	0.90	0.24	3.81	0.000	0.004
Malassezia.pachydermatis	70.43	1.39	0.37	3.79	0.000	0.004
Faecalibaculum.rodentium	1209.90	1.21	0.32	3.78	0.000	0.004
Corynebacterium.deserti	64.06	0.80	0.21	3.78	0.000	0.004
Kingella.kingae	433.19	1.91	0.51	3.77	0.000	0.004
Cyphellophora.europaea	71.86	-0.74	0.20	-3.77	0.000	0.004
Thermogymnomonas.acidicola	21.48	-1.63	0.43	-3.76	0.000	0.004
Haloarcula.marismortui	8.06	-2.43	0.65	-3.75	0.000	0.004
Bifidobacterium.angulatum	528.07	1.56	0.42	3.75	0.000	0.004
Nitrobacter.winogradskyi	99.67	-0.89	0.24	-3.74	0.000	0.004
Cyclobacterium.marinum	145.54	-1.06	0.28	-3.72	0.000	0.005
Bradyrhizobiaceae.bacterium.SG.6C	154.28	-0.50	0.14	-3.72	0.000	0.005
Arthrospira.platensis	440.61	0.62	0.17	3.72	0.000	0.005
Geobacter.sulfurreducens	319.50	-0.55	0.15	-3.70	0.000	0.005
Gemella.sp..oral.taxon.928	748.20	1.25	0.34	3.69	0.000	0.005
Thauera.sp..MZ1T	238.95	-0.54	0.15	-3.67	0.000	0.005
Stanieria.cyanosphaera	567.76	0.67	0.18	3.67	0.000	0.006
Alcanivorax.pacificus	196.30	-0.62	0.17	-3.65	0.000	0.006
Aminobacterium.colombiense	188.98	1.63	0.45	3.64	0.000	0.006
Fibrobacter.succinogenes	3018760.67	1.41	0.39	3.64	0.000	0.006
Rickettsia.canadensis	81.14	1.47	0.41	3.62	0.000	0.006
Pseudomonas.sp..MRSN12121	15.83	-1.21	0.34	-3.61	0.000	0.007
Desulfovibrio.legallii	1426.74	-0.97	0.27	-3.58	0.000	0.007

Methanosphaera.sp..A6	6.96	2.89	0.81	3.58	0.000	0.007
Halostagnicola.larsenii	41.63	-1.45	0.41	-3.56	0.000	0.008
Haladaptatus.paucihalophilus	22.18	-1.35	0.38	-3.56	0.000	0.008
Streptomyces.avermitilis	181.13	0.60	0.17	3.56	0.000	0.008
Corynebacterium.ammoniagenes	74.38	0.94	0.26	3.55	0.000	0.008
Cedecea.neteri	336.49	-0.57	0.16	-3.55	0.000	0.008
Turneriella.parva	139.94	-1.09	0.31	-3.54	0.000	0.008
Candidatus.Symbiobacter.mobilis	361.97	1.48	0.42	3.54	0.000	0.008
Cyanothece.sp..PCC.8801	17.82	-2.49	0.71	-3.53	0.000	0.008
Trueperella.pyogenes	282.94	0.76	0.21	3.53	0.000	0.008
Sulfurihydrogenibium.sp..YO3AOP1	404.79	1.05	0.30	3.52	0.000	0.009
Methanolobus.psychrophilus	41.91	-0.74	0.21	-3.52	0.000	0.009
X.Eubacterium..cellulosolvens	22006.02	0.69	0.20	3.52	0.000	0.009
Pseudoalteromonas.rubra	60.06	-0.77	0.22	-3.51	0.000	0.009
Candidatus.Xiphinematobacter.sp..Idaho.Grape	37.10	-1.19	0.34	-3.51	0.000	0.009
Haloterrigena.daqingensis	21.17	-1.67	0.48	-3.51	0.000	0.009
Staphylococcus.condimenti	336.36	-1.19	0.34	-3.50	0.000	0.009
Pseudarthrobacter.chlorophenolicus	301.61	0.77	0.22	3.49	0.000	0.009
Streptomyces.davaonensis	159.60	0.79	0.23	3.49	0.000	0.009
Thermosynechococcus.sp..NK55a	71.02	1.12	0.32	3.48	0.000	0.009
Ruminococcus.sp..YE78	1908.25	1.10	0.31	3.48	0.001	0.010
Lactobacillus.delbrueckii	524.66	0.54	0.16	3.47	0.001	0.010
Haloarcula.salaria	3.62	-2.47	0.71	-3.47	0.001	0.010
Zunongwangia.profunda	116.64	-0.89	0.26	-3.47	0.001	0.010
Streptococcus.uberis	277.15	0.81	0.24	3.46	0.001	0.010
Borrelia.hermsii	251.04	0.62	0.18	3.45	0.001	0.010
Corynebacterium.simulans	230.36	0.94	0.27	3.45	0.001	0.010
Aciduliprofundum.boonei	21.74	-1.60	0.47	-3.44	0.001	0.011
Dorea.longicatena	58961.58	2.77	0.81	3.44	0.001	0.011
Halobacterium.salinarum	51.58	1.60	0.47	3.44	0.001	0.011
Faecalicatena.contorta	2626.79	1.09	0.32	3.43	0.001	0.011
Ruminobacter.sp..RM87	350.58	-1.69	0.49	-3.43	0.001	0.011
Chitinophaga.pinensis	577.96	-0.84	0.25	-3.42	0.001	0.011
Burkholderia.stagnalis	80.63	-0.94	0.28	-3.41	0.001	0.011
Dickeya.sp..NCPPB.3274	44.42	1.27	0.37	3.40	0.001	0.012
Croceicoccus.naphthovorans	152.80	0.75	0.22	3.39	0.001	0.012
Blautia.sp..SF.50	5235.87	1.02	0.30	3.39	0.001	0.012
Haloarcula.vallismortis	24.68	-2.08	0.62	-3.38	0.001	0.012
Fervidobacterium.nodosum	310.29	1.90	0.56	3.38	0.001	0.012
Paludisphaera.borealis	246.35	-0.66	0.20	-3.38	0.001	0.012
Haloferax.elongans	14.78	-1.33	0.39	-3.38	0.001	0.012
Streptococcus.suis	594.08	0.51	0.15	3.38	0.001	0.012
Synechococcus.sp..SynAce01	114.38	0.72	0.21	3.38	0.001	0.012
Olsenella.sp..oral.taxon.807	2777.44	0.98	0.29	3.37	0.001	0.012
Dehalobacter.sp..DCA	3.98	3.12	0.93	3.37	0.001	0.012
Limnohabitans.sp..103DPR2	58.61	-0.97	0.29	-3.37	0.001	0.012
Anaeromyxobacter.sp..Fw109.5	365.57	-0.51	0.15	-3.36	0.001	0.013
Phaeobacter.inhibens	111.74	-0.87	0.26	-3.36	0.001	0.013
Nitrosospira.multiformis	115.64	-1.29	0.39	-3.36	0.001	0.013
Streptococcus.thermophilus	499.95	0.86	0.26	3.35	0.001	0.013
Borrelia.parkeri	33.22	1.30	0.39	3.35	0.001	0.013

Bacteroides.vulgatus	2459.55	-0.82	0.25	-3.33	0.001	0.014
Helicobacter.typhlonius	58.65	-1.42	0.43	-3.33	0.001	0.014
Caldisericum.exile	133.64	-0.97	0.29	-3.33	0.001	0.014
Myxococcus.fulvus	425.79	-0.57	0.17	-3.33	0.001	0.014
Yersinia.intermedia	41.16	-1.08	0.33	-3.33	0.001	0.014
Pseudomonas.psychrotolerans	190.01	-0.72	0.22	-3.32	0.001	0.014
Corynebacterium.falsenii	127.85	0.77	0.23	3.31	0.001	0.015
Jeotgalicoccus.saudimassiliensis	339.58	1.29	0.39	3.30	0.001	0.015
Lachnobacterium.bovis	13776.63	0.62	0.19	3.30	0.001	0.015
Candidatus.Cloacimonas.acidaminovorans	111.31	-0.82	0.25	-3.30	0.001	0.015
Halosimplex.carlsbadense	50.37	-0.77	0.24	-3.29	0.001	0.015
Candidatus.Nitrosopumilus.adriaticus	108.31	1.33	0.41	3.29	0.001	0.015
Escherichia.albertii	52.01	0.67	0.21	3.28	0.001	0.016
Nocardioides.dokdonensis	243.10	0.68	0.21	3.28	0.001	0.016
Lachnospiraceae.bacterium.FD2005	2818.94	1.51	0.46	3.28	0.001	0.016
Arthrobacter.sp..ERGS1.01	293.23	0.71	0.22	3.27	0.001	0.016
Methanoculleus.thermophilus	90.33	1.37	0.42	3.26	0.001	0.016
Lachnospiraceae.bacterium.ND2006	3161.87	0.76	0.23	3.25	0.001	0.017
Brevibacillus.brevis	342.16	0.97	0.30	3.24	0.001	0.018
Pelosinus.fermentans	787.26	0.36	0.11	3.24	0.001	0.018
Scardovia.inopinata	126.25	0.92	0.29	3.24	0.001	0.018
Sphingopyxis.fribergensis	105.08	-1.01	0.31	-3.23	0.001	0.018
Pusillimonas.sp..T7.7	115.29	-0.68	0.21	-3.23	0.001	0.018
Dokdonia.sp..MED134	48.77	-1.12	0.35	-3.22	0.001	0.018
Acinetobacter.haemolyticus	139.30	1.37	0.42	3.22	0.001	0.018
Acetobacter.oryzifermentans	15.55	-1.10	0.34	-3.21	0.001	0.019
Adlercreutzia.equolifaciens	3821.08	0.77	0.24	3.21	0.001	0.019
Hyphomonas.neptunium	124.17	-0.67	0.21	-3.21	0.001	0.019
Streptomyces.anulatus	58.36	-0.56	0.18	-3.20	0.001	0.019
Brachyspira.pilosicoli	1197.87	1.00	0.31	3.20	0.001	0.019
Clostridioides.difficile	3418.46	0.70	0.22	3.20	0.001	0.019
Phycisphaera.mikurensis	227.15	-0.53	0.17	-3.20	0.001	0.019
Sorangium.cellulosum	718.48	-0.42	0.13	-3.20	0.001	0.019
Prosthecochloris.sp..CIB.2401	102.44	-0.59	0.19	-3.19	0.001	0.019
Eremothecium.gossypii	196.31	1.49	0.47	3.19	0.001	0.019
Desulfovibrio.africanus	528.20	-0.65	0.20	-3.19	0.001	0.020
Hafnia.alvei	124.18	-0.73	0.23	-3.19	0.001	0.020
Pseudomonas.sp..URMO17WK12.I11	275.44	0.96	0.30	3.18	0.001	0.020
Sulfolobus.acidocaldarius	53.61	-2.05	0.64	-3.18	0.001	0.020
Halioglobus.pacificus	120.80	0.66	0.21	3.17	0.002	0.020
Verminephrobacter.eiseniae	247.44	0.51	0.16	3.17	0.002	0.021
Acinetobacter.sp..DSM.11652	123.15	1.21	0.38	3.16	0.002	0.021
Rhodococcus.aetherivorans	59.13	1.41	0.45	3.16	0.002	0.021
Echinicola.vietnamensis	220.49	0.89	0.28	3.16	0.002	0.021
Bifidobacterium.sp..AGR2158	300.83	0.63	0.20	3.16	0.002	0.021
Natronorubrum.thiooxidans	10.68	-1.43	0.45	-3.14	0.002	0.022
Thermococcus.cleftensis	11.20	-1.13	0.36	-3.14	0.002	0.022
Kosmotoga.pacifica	81.99	1.68	0.54	3.14	0.002	0.022
Dyella.jiangningensis	240.29	0.61	0.19	3.14	0.002	0.022
Hydrogenobacter.thermophilus	30.49	-1.08	0.35	-3.14	0.002	0.022
Frankia.casuarinae	109.66	-0.50	0.16	-3.13	0.002	0.022

Campylobacter.hominis	319.52	0.75	0.24	3.13	0.002	0.023
Oscillatoria.acuminata	306.60	0.65	0.21	3.13	0.002	0.023
Candidatus.Paracaedibacter.acanthamoebae	59.28	-1.09	0.35	-3.12	0.002	0.023
Marinobacter.sp..CP1	57.25	0.85	0.27	3.12	0.002	0.023
Vibrio.campbellii	50.77	1.51	0.49	3.11	0.002	0.024
Bacillus.sp..JS	97.69	-1.41	0.45	-3.11	0.002	0.024
Defluviitoga.tunisiensis	179.97	0.74	0.24	3.10	0.002	0.024
Bacillus.sp..FJAT.18017	184.30	0.76	0.24	3.10	0.002	0.024
X.Eubacterium..rectale	4140.44	0.63	0.20	3.10	0.002	0.024
Klebsiella.michiganensis	123.91	-0.75	0.24	-3.09	0.002	0.024
Encephalitozoon.intestinalis	111.39	1.94	0.63	3.09	0.002	0.024
Brucella.sp..141012304	18.17	-1.49	0.48	-3.09	0.002	0.024
Glaciecola.sp..4H.3.7.YE.5	43.66	-1.10	0.36	-3.09	0.002	0.025
Shewanella.pealeana	40.18	-0.95	0.31	-3.08	0.002	0.026
Enterobacter.ludwigii	33.58	-1.74	0.57	-3.06	0.002	0.027
Clostridium.aceticum	507.48	0.80	0.26	3.06	0.002	0.027
Methanomethylovorans.hollandica	28.34	-0.79	0.26	-3.06	0.002	0.027
Peptostreptococcaceae.bacterium.pGA.8	1926.41	0.55	0.18	3.06	0.002	0.027
Lachnospiraceae.bacterium.KH1T2	2532.19	0.71	0.23	3.06	0.002	0.027
Corynebacterium.urealyticum	147.69	0.71	0.23	3.05	0.002	0.027
Ruminobacter.amylophilus	437.17	-2.42	0.80	-3.05	0.002	0.028
Halomonas.sp..A3H3	114.52	0.92	0.30	3.04	0.002	0.028
Dictyoglomus.turgidum	242.44	0.83	0.27	3.04	0.002	0.028
Pediococcus.claussenii	217.90	1.02	0.33	3.04	0.002	0.028
Ignicoccus.islandicus	1.93	-4.22	1.39	-3.04	0.002	0.028
Streptococcus.mitis	245.19	0.86	0.28	3.04	0.002	0.028
Porphyromonadaceae.bacterium.NLAE.zl.C104	82.19	-1.04	0.34	-3.03	0.002	0.028
Comamonas.testosteroni	633.06	0.56	0.19	3.03	0.002	0.029
Burkholderia.plantarii	17.04	-1.06	0.35	-3.02	0.003	0.029
Leptospira.borgpetersenii	105.30	-1.55	0.51	-3.01	0.003	0.030
Halomicrobium.mukohataei	15.99	-1.15	0.38	-3.01	0.003	0.030
Rhodococcus.sp..PBTS.1	289.12	0.78	0.26	3.01	0.003	0.030
Mycoplasma.canadense	359.32	0.65	0.22	3.01	0.003	0.030
Arcanobacterium.haemolyticum	52.17	-0.80	0.27	-3.00	0.003	0.031
Thiobacillus.denitrificans	171.76	-0.51	0.17	-3.00	0.003	0.031
Psychrobacter.sp..P2G3	12.84	1.52	0.51	3.00	0.003	0.031
Rickettsia.raoultii	5.18	2.46	0.82	2.99	0.003	0.031
X.Ruminococcus..gnavus	4320.43	0.73	0.24	2.99	0.003	0.031
Selenomonas.sp..ND2010	4374.71	1.47	0.49	2.99	0.003	0.031
Granulicella.mallensis	158.65	-0.49	0.16	-2.98	0.003	0.032
Synechococcus.sp..WH.7803	98.84	-1.36	0.45	-2.98	0.003	0.032
Anaeromyxobacter.sp..K	74.91	-0.63	0.21	-2.98	0.003	0.032
Halobellus.rufus	25.20	-0.87	0.29	-2.97	0.003	0.034
Alcaligenes.faecalis	117.25	-0.61	0.21	-2.96	0.003	0.034
Selenomonas.sp..FC4001	13427.85	1.73	0.59	2.96	0.003	0.034
Methanocaldococcus.vulcanius	201.55	1.12	0.38	2.95	0.003	0.035
Brevundimonas.subvibrioides	197.35	-0.67	0.23	-2.95	0.003	0.035
Polynucleobacter.asymbioticus	37.68	-0.87	0.29	-2.95	0.003	0.035
Acinetobacter.larvae	59.74	0.70	0.24	2.95	0.003	0.035
Actinotignum.schaalii	131.18	0.71	0.24	2.95	0.003	0.035
Mycolicibacterium.fortuitum	41.23	-1.07	0.36	-2.95	0.003	0.035

Methylomonas.denitrificans	153.27	-0.81	0.28	-2.94	0.003	0.035
Methylocella.silvestris	123.25	-0.57	0.19	-2.94	0.003	0.035
Sandaracinus.amylolyticus	307.14	-0.42	0.14	-2.94	0.003	0.035
Polynucleobacter.wuianus	75.41	0.98	0.33	2.94	0.003	0.036
Halomonas.sp..HL.93	115.72	0.71	0.24	2.91	0.004	0.038
Faecalitalea.cylindroides	2651.14	0.83	0.28	2.91	0.004	0.039
Corynebacterium.ulcerans	176.15	0.84	0.29	2.91	0.004	0.039
Haemophilus.influenzae	420.93	0.68	0.24	2.91	0.004	0.039
Eggerthella.sp..YY7918	1823.35	0.69	0.24	2.90	0.004	0.040
Babesia.bigemina	77371.32	1.35	0.47	2.90	0.004	0.040
Candidatus.Arthromitus.sp..SFB.rat.Yit	550.96	0.93	0.32	2.89	0.004	0.040
Shinella.sp..HZN7	273.84	-0.57	0.20	-2.89	0.004	0.040
Methanomassiliicoccus.luminyensis	296.87	-0.84	0.29	-2.88	0.004	0.041
Desulfovibrio.alaskensis	406.18	-0.43	0.15	-2.89	0.004	0.041
Methylocystis.sp..SC2	225.97	-0.84	0.29	-2.88	0.004	0.041
Olsenella.uli	4422.72	0.88	0.31	2.86	0.004	0.043
Cyanothece.sp..PCC.8802	17.44	-1.43	0.50	-2.86	0.004	0.043
Halorubrum.arcis	15.09	1.11	0.39	2.86	0.004	0.043
Singulisphaera.acidiphila	218.96	-0.64	0.22	-2.85	0.004	0.044
Burkholderia.stabilis	54.07	-0.62	0.22	-2.85	0.004	0.044
Borrelia.crocidurae	111.28	1.32	0.46	2.85	0.004	0.044
Rhodobacter.capsulatus	164.08	-0.69	0.24	-2.84	0.004	0.045
Sphingomonas.sp..JJ.A5	109.16	-0.69	0.24	-2.84	0.004	0.045
Streptomyces.reticuli	136.95	-0.58	0.20	-2.84	0.005	0.046
Haloprofundus.marisrubri	28.74	0.87	0.31	2.83	0.005	0.046
Mycoplasma.pneumoniae	38.19	1.03	0.37	2.82	0.005	0.048
Campylobacter.peloridis	205.39	1.05	0.37	2.82	0.005	0.048
Methylovorus.sp..MP688	39.45	-0.72	0.26	-2.81	0.005	0.049
Novosphingobium.pentaromativorans	84.65	-0.81	0.29	-2.81	0.005	0.049
Cryobacterium.arcticum	250.18	0.48	0.17	2.81	0.005	0.049
Lachnospiraceae.bacterium.P6A3	16145.22	0.82	0.29	2.81	0.005	0.049
Peptostreptococcus.sp..D1	521.73	0.54	0.19	2.81	0.005	0.049
Mannheimia.varigena	232.94	0.97	0.35	2.81	0.005	0.049
Lachnospiraceae.bacterium.G11	45598.23	0.98	0.35	2.81	0.005	0.049
Colwellia.psychrerythraea	111.71	-0.79	0.28	-2.80	0.005	0.049
Deinococcus.maricopensis	182.78	-0.46	0.17	-2.80	0.005	0.049
Haloferax.denitrificans	20.19	-2.41	0.86	-2.80	0.005	0.049
Bartonella.australis	27.73	-0.89	0.32	-2.80	0.005	0.049

Supplementary table 5 DESeq2 test results at the Species level

Significantly different species between low (n=8 animals) and high (n=8 animals) emitting sheep. Table and statistics calculated using DESeq2. Mean is the average normalised counts across all samples; log2FC is the log2 ratio methane emissions low/high; lfcSE gives the standard error of the log2FC; stat is the Wald statistic: the log2FC divided by lfcSE, which is compared to a standard Normal distribution to generate a two-tailed pvalue; pvalue is the raw p-value, and padj is the adjusted p-value (Benjamini and Hochberg (1995) Journal of the Royal Statistical Society. Series B (Methodological), Vol. 57, No. 1 (1995), pp. 289-300, also known as FDR)
