# Supplementary material for: Compendium of 4,941 rumen metagenome-assembled genomes for rumen microbiome biology and enzyme discovery
Source: Nat Biotechnol. 2019 Aug 2;37(8):953–61. doi: 10.1038/s41587-019-0202-3 (PMC6785717; doi:10.1038/s41587-019-0202-3)
Supplement: Supplementary file 17 — Reporting Summary [file 41587_2019_202_MOESM2_ESM.pdf]

nature research

Corresponding author(s): Mick Watson

Last updated by author(s): Jun 14, 2019

Reporting Summary

Nature Research wishes to improve the reproducibility of the work that we publish. This form provides structure for consistency and transparency in reporting. For further information on Nature Research policies, see Authors & Referees and the Editorial Policy Checklist.

Statistics

For all statistical analyses, confirm that the following items are present in the figure legend, table legend, main text, or Methods section.

n/a	Confirmed	
☐	☒	The exact sample size (*n*) for each experimental group/condition, given as a discrete number and unit of measurement
☐	☒	A statement on whether measurements were taken from distinct samples or whether the same sample was measured repeatedly
☐	☒	The statistical test(s) used AND whether they are one- or two-sided<br>*Only common tests should be described solely by name; describe more complex techniques in the Methods section.*
☐	☒	A description of all covariates tested
☐	☒	A description of any assumptions or corrections, such as tests of normality and adjustment for multiple comparisons
☐	☒	A full description of the statistical parameters including central tendency (e.g. means) or other basic estimates (e.g. regression coefficient) AND variation (e.g. standard deviation) or associated estimates of uncertainty (e.g. confidence intervals)
☐	☒	For null hypothesis testing, the test statistic (e.g. *F*, *t*, *r*) with confidence intervals, effect sizes, degrees of freedom and *P* value noted<br>*Give P values as exact values whenever suitable.*
☒	☐	For Bayesian analysis, information on the choice of priors and Markov chain Monte Carlo settings
☒	☐	For hierarchical and complex designs, identification of the appropriate level for tests and full reporting of outcomes
☒	☐	Estimates of effect sizes (e.g. Cohen's *d*, Pearson's *r*), indicating how they were calculated

Our web collection on statistics for biologists contains articles on many of the points above.

Software and code

Policy information about availability of computer code

Data collection	Comparative genomic analysis was carried out using MAGpy (https://github.com/WatsonLab/MAGpy); analysis of PUL was carried out using PULpy (https://github.com/WatsonLab/PULpy); analysis of indels in nanopore data was carried out using IDEEL (https://github.com/mw55309/ideel); other open source software used: idba_ud (v1.1.3), BWA MEM (v0.7.15), Samtools (v1.3.1), MetaBAT2 (v2.11.1), MEGAHIT (v1.1.1), dRep (v1.1.2), CheckM (v1.0.5), prodigal (v2.6.3), Pfam_Scan (v1.6), DIMAOND (v0.9.22.123), PhyloPhlAn (v0.99), Sourmash (v2.0.0), FigTree (v.1.4.3), iTol (v4.3.1) and GraPhlAn (v0.9.7), tRNAscan-SE (v2.0.0), barrnap (v0.9), MUMmer (v3.23), FastANI (v1.1), MASH (v2.0), Kraken (v0.10.5), DESeq2 (v1.22.2), gplots (v3.0.1.1), poRe (v0.24), Canu (v1.8), Nanopolish (v0.10.2), Racon (v1.3.1), Minimap2 (v2.12), using Prokka (v1.13.3), CD-HIT (v4.7), dbCAN (v2), HMMER (v3.1b2)
Data analysis	Comparative genomic analysis was carried out using MAGpy (https://github.com/WatsonLab/MAGpy); analysis of PUL was carried out using PULpy (https://github.com/WatsonLab/PULpy); analysis of indels in nanopore data was carried out using IDEEL (https://github.com/mw55309/ideel); other open source software used: idba_ud (v1.1.3), BWA MEM (v0.7.15), Samtools (v1.3.1), MetaBAT2 (v2.11.1), MEGAHIT (v1.1.1), dRep (v1.1.2), CheckM (v1.0.5), prodigal (v2.6.3), Pfam_Scan (v1.6), DIMAOND (v0.9.22.123), PhyloPhlAn (v0.99), Sourmash (v2.0.0), FigTree (v.1.4.3), iTol (v4.3.1) and GraPhlAn (v0.9.7), tRNAscan-SE (v2.0.0), barrnap (v0.9), MUMmer (v3.23), FastANI (v1.1), MASH (v2.0), Kraken (v0.10.5), DESeq2 (v1.22.2), gplots (v3.0.1.1), poRe (v0.24), Canu (v1.8), Nanopolish (v0.10.2), Racon (v1.3.1), Minimap2 (v2.12), using Prokka (v1.13.3), CD-HIT (v4.7), dbCAN (v2), HMMER (v3.1b2)

For manuscripts utilizing custom algorithms or software that are central to the research but not yet described in published literature, software must be made available to editors/reviewers. We strongly encourage code deposition in a community repository (e.g. GitHub). See the Nature Research guidelines for submitting code & software for further information.

Data

Policy information about availability of data

All manuscripts must include a data availability statement. This statement should provide the following information, where applicable:

- Accession codes, unique identifiers, or web links for publicly available datasets
- A list of figures that have associated raw data
- A description of any restrictions on data availability

Raw sequence reads for all samples are available under ENA project PRJEB31266, except for 10572 which are available under PRJEB21624. All metagenomic assemblies and RUGs are in the process of being deposited in ENA under accession PRJEB31266. All protein predictions, clusters and annotation are available at DOI: 10.7488/ds/2470.

Field-specific reporting

Please select the one below that is the best fit for your research. If you are not sure, read the appropriate sections before making your selection.

☒ Life sciences ☐ Behavioural & social sciences ☐ Ecological, evolutionary & environmental sciences

For a reference copy of the document with all sections, see nature.com/documents/nr-reporting-summary-flat.pdf

Life sciences study design

All studies must disclose on these points even when the disclosure is negative.

Sample size	As primarily a discovery project, sample size was not important, and we examined as many samples as possible.<br>Where we carried out statistical analysis, those sample sizes were determined by the authors of those studies (e.g. Shi et al)
Data exclusions	No data were excluded
Replication	As a discovery project, replication is not important
Randomization	As a discovery project, no randomization was required
Blinding	As a discovery project, no blinding was required

Reporting for specific materials, systems and methods

We require information from authors about some types of materials, experimental systems and methods used in many studies. Here, indicate whether each material, system or method listed is relevant to your study. If you are not sure if a list item applies to your research, read the appropriate section before selecting a response.

Materials & experimental systems

n/a	Involved in the study	
☒	☐	Antibodies
☒	☐	Eukaryotic cell lines
☒	☐	Palaeontology
☐	☒	Animals and other organisms
☒	☐	Human research participants
☒	☐	Clinical data

Methods

n/a	Involved in the study	
☒	☐	ChIP-seq
☒	☐	Flow cytometry
☒	☐	MRI-based neuroimaging

Animals and other organisms

Policy information about studies involving animals; ARRIVE guidelines recommended for reporting animal research

Laboratory animals	The data were obtained from three cross breeds: Aberdeen Angus, Limousin and Charolais and one pure breed: Luing; All animals were male and between 459 and 661 days old
Wild animals	the study did not involve wild animals.
Field-collected samples	the study did not involve field-collected samples
Ethics oversight	Animal experiments were conducted at the Beef and Sheep Research Centre of Scotland's Rural College (SRUC). The experiment was approved by the Animal Experiment Committee of SRUC and was conducted in accordance with the requirements of the UK

Animals (Scientific Procedures) Act 1986.

Note that full information on the approval of the study protocol must also be provided in the manuscript.
